# Supplementary material for: The influence of social power on neural responses to emotional conflict
Source: PeerJ. 2021 Apr 12;9:e11267. doi: 10.7717/peerj.11267 (PMC8048403; doi:10.7717/peerj.11267)
Supplement: Supplemental Information 2 [file peerj-09-11267-s002.doc]

**All the faces in RT**

NPAR TESTS

/M-W= s11fear congruent conditions s21happyincongruent conditions s31fearincongruent conditions s41happycongruent conditions BY power(1 2)

/STATISTICS=DESCRIPTIVES

/MISSING ANALYSIS.

**Mann-Whitney Test**

| **Ranks** | | | | |
| --- | --- | --- | --- | --- |
|  | power | N | Mean Rank | Sum of Ranks |
| s11fear congruent conditions | 1.00 | 19 | 20.63 | 392.00 |
|  | 2.00 | 19 | 18.37 | 349.00 |
|  | Total | 38 |  |  |
| s21happy incongruent conditions | 1.00 | 19 | 22.63 | 430.00 |
|  | 2.00 | 19 | 16.37 | 311.00 |
|  | Total | 38 |  |  |
| s31fear incongruent conditions | 1.00 | 19 | 21.21 | 403.00 |
|  | 2.00 | 19 | 17.79 | 338.00 |
|  | Total | 38 |  |  |
| s41happy congruent conditions | 1.00 | 19 | 21.53 | 409.00 |
|  | 2.00 | 19 | 17.47 | 332.00 |
|  | Total | 38 |  |  |

| **Test Statistics^a^** | | | | |
| --- | --- | --- | --- | --- |
|  | s11fearcongruent conditions | s21happyincongruent conditions | s31fearincongruent conditions | s41happycongruent conditions |
| Mann-Whitney U | 159.000 | 121.000 | 148.000 | 142.000 |
| Wilcoxon W | 349.000 | 311.000 | 338.000 | 332.000 |
| Z | -.628 | -1.737 | -.949 | -1.124 |
| Asymp. Sig. (2-tailed) | .530 | .082 | .343 | .261 |
| Exact Sig. [2*(1-tailed Sig.)] | .544^b^ | .085^b^ | .354^b^ | .271^b^ |
| a. Grouping Variable: power | | | | |
| b. Not corrected for ties. | | | | |

**All the faces in ACC**

**Mann-Whitney Test**

| **Ranks** | | | | |
| --- | --- | --- | --- | --- |
|  | power | N | Mean Rank | Sum of Ranks |
| ACC_fear_congruent | 1.00 | 19 | 19.58 | 372.00 |
|  | 2.00 | 19 | 19.42 | 369.00 |
|  | Total | 38 |  |  |
| ACC_happy_congruent | 1.00 | 19 | 18.53 | 352.00 |
|  | 2.00 | 19 | 20.47 | 389.00 |
|  | Total | 38 |  |  |
| ACC_fear_incongruent | 1.00 | 19 | 17.71 | 336.50 |
|  | 2.00 | 19 | 21.29 | 404.50 |
|  | Total | 38 |  |  |
| ACC_happy_incongruent | 1.00 | 19 | 18.89 | 359.00 |
|  | 2.00 | 19 | 20.11 | 382.00 |
|  | Total | 38 |  |  |

| **Test Statistics^a^** | | | | |
| --- | --- | --- | --- | --- |
|  | ACC_fear_congruent | ACC_happy_congruent | ACC_fear_incongruent | ACC_happy_incongruent |
| Mann-Whitney U | 179.000 | 162.000 | 146.500 | 169.000 |
| Wilcoxon W | 369.000 | 352.000 | 336.500 | 359.000 |
| Z | -.044 | -.542 | -.995 | -.336 |
| Asymp. Sig. (2-tailed) | .965 | .588 | .320 | .737 |
| Exact Sig. [2*(1-tailed Sig.)] | .977^b^ | .603^b^ | .325^b^ | .751^b^ |
| a. Grouping Variable: power | | | | |
| b. Not corrected for ties. | | | | |

**All the faces in P1**

NPAR TESTS

/M-W= P7congruent conditions P7incongruent conditions P8congruent conditions P8incongruent conditions BY power(1 2)

/MISSING ANALYSIS.

**Mann-Whitney Test**

| **Ranks** | | | | |
| --- | --- | --- | --- | --- |
|  | power | N | Mean Rank | Sum of Ranks |
| P7congruent conditions | 1.00 | 19 | 16.95 | 322.00 |
|  | 2.00 | 19 | 22.05 | 419.00 |
|  | Total | 38 |  |  |
| P7incongruent conditions | 1.00 | 19 | 17.42 | 331.00 |
|  | 2.00 | 19 | 21.58 | 410.00 |
|  | Total | 38 |  |  |
| P8congruent conditions | 1.00 | 19 | 16.26 | 309.00 |
|  | 2.00 | 19 | 22.74 | 432.00 |
|  | Total | 38 |  |  |
| P8incongruent conditions | 1.00 | 19 | 16.32 | 310.00 |
|  | 2.00 | 19 | 22.68 | 431.00 |
|  | Total | 38 |  |  |

| **Test Statistics^a^** | | | | |
| --- | --- | --- | --- | --- |
|  | P7congruent conditions | P7incongruent conditions | P8congruent conditions | P8incongruent conditions |
| Mann-Whitney U | 132.000 | 141.000 | 119.000 | 120.000 |
| Wilcoxon W | 322.000 | 331.000 | 309.000 | 310.000 |
| Z | -1.416 | -1.153 | -1.795 | -1.766 |
| Asymp. Sig. (2-tailed) | .157 | .249 | .073 | .077 |
| Exact Sig. [2*(1-tailed Sig.)] | .163^b^ | .258^b^ | .075^b^ | .080^b^ |
| a. Grouping Variable: power | | | | |
| b. Not corrected for ties. | | | | |

**All the faces in N170**

NPAR TESTS

/M-W= P7congruent conditions P7incongruent conditions P8congruent conditions P8incongruent conditions BY power(1 2)

/MISSING ANALYSIS.

**Mann-Whitney Test**

| **Ranks** | | | | |
| --- | --- | --- | --- | --- |
|  | power | N | Mean Rank | Sum of Ranks |
| P7congruent conditions | 1.00 | 19 | 17.26 | 328.00 |
|  | 2.00 | 19 | 21.74 | 413.00 |
|  | Total | 38 |  |  |
| P7incongruent conditions | 1.00 | 19 | 17.16 | 326.00 |
|  | 2.00 | 19 | 21.84 | 415.00 |
|  | Total | 38 |  |  |
| P8congruent conditions | 1.00 | 19 | 16.63 | 316.00 |
|  | 2.00 | 19 | 22.37 | 425.00 |
|  | Total | 38 |  |  |
| P8incongruent conditions | 1.00 | 19 | 16.63 | 316.00 |
|  | 2.00 | 19 | 22.37 | 425.00 |
|  | Total | 38 |  |  |

| **Test Statistics^a^** | | | | |
| --- | --- | --- | --- | --- |
|  | P7congruent conditions | P7incongruent conditions | P8congruent conditions | P8incongruent conditions |
| Mann-Whitney U | 138.000 | 136.000 | 126.000 | 126.000 |
| Wilcoxon W | 328.000 | 326.000 | 316.000 | 316.000 |
| Z | -1.241 | -1.299 | -1.591 | -1.591 |
| Asymp. Sig. (2-tailed) | .215 | .194 | .112 | .112 |
| Exact Sig. [2*(1-tailed Sig.)] | .223^b^ | .201^b^ | .116^b^ | .116^b^ |
| a. Grouping Variable: power | | | | |
| b. Not corrected for ties. | | | | |

**All the faces in N450**

NPAR TESTS

/M-W= F3congruent conditions F3incongruent conditions F4congruent conditions F4incongruent conditions FZcongruent conditions FZincongruent conditions C3congruent conditions C3incongruent conditions C4congruent conditions C4incongruent conditions CZcongruent conditions CZincongruent conditions CP1congruent conditions CP1incongruent conditions CP2congruent conditions CP2incongruent conditions BY power(1 2)

/MISSING ANALYSIS.

**Mann-Whitney Test**

| **Ranks** | | | | |
| --- | --- | --- | --- | --- |
|  | power | N | Mean Rank | Sum of Ranks |
| F3congruent conditions | 1.00 | 19 | 17.79 | 338.00 |
|  | 2.00 | 19 | 21.21 | 403.00 |
|  | Total | 38 |  |  |
| F3incongruent conditions | 1.00 | 19 | 18.32 | 348.00 |
|  | 2.00 | 19 | 20.68 | 393.00 |
|  | Total | 38 |  |  |
| F4congruent conditions | 1.00 | 19 | 19.89 | 378.00 |
|  | 2.00 | 19 | 19.11 | 363.00 |
|  | Total | 38 |  |  |
| F4incongruent conditions | 1.00 | 19 | 19.84 | 377.00 |
|  | 2.00 | 19 | 19.16 | 364.00 |
|  | Total | 38 |  |  |
| FZcongruent conditions | 1.00 | 19 | 18.63 | 354.00 |
|  | 2.00 | 19 | 20.37 | 387.00 |
|  | Total | 38 |  |  |
| FZincongruent conditions | 1.00 | 19 | 18.74 | 356.00 |
|  | 2.00 | 19 | 20.26 | 385.00 |
|  | Total | 38 |  |  |
| C3congruent conditions | 1.00 | 19 | 19.05 | 362.00 |
|  | 2.00 | 19 | 19.95 | 379.00 |
|  | Total | 38 |  |  |
| C3incongruent conditions | 1.00 | 19 | 20.11 | 382.00 |
|  | 2.00 | 19 | 18.89 | 359.00 |
|  | Total | 38 |  |  |
| C4congruent conditions | 1.00 | 19 | 19.95 | 379.00 |
|  | 2.00 | 19 | 19.05 | 362.00 |
|  | Total | 38 |  |  |
| C4incongruent conditions | 1.00 | 19 | 20.05 | 381.00 |
|  | 2.00 | 19 | 18.95 | 360.00 |
|  | Total | 38 |  |  |
| CZcongruent conditions | 1.00 | 19 | 19.68 | 374.00 |
|  | 2.00 | 19 | 19.32 | 367.00 |
|  | Total | 38 |  |  |
| CZincongruent conditions | 1.00 | 19 | 19.74 | 375.00 |
|  | 2.00 | 19 | 19.26 | 366.00 |
|  | Total | 38 |  |  |
| CP1congruent conditions | 1.00 | 19 | 18.37 | 349.00 |
|  | 2.00 | 19 | 20.63 | 392.00 |
|  | Total | 38 |  |  |
| CP1incongruent conditions | 1.00 | 19 | 19.32 | 367.00 |
|  | 2.00 | 19 | 19.68 | 374.00 |
|  | Total | 38 |  |  |
| CP2congruent conditions | 1.00 | 19 | 19.47 | 370.00 |
|  | 2.00 | 19 | 19.53 | 371.00 |
|  | Total | 38 |  |  |
| CP2incongruent conditions | 1.00 | 19 | 19.95 | 379.00 |
|  | 2.00 | 19 | 19.05 | 362.00 |
|  | Total | 38 |  |  |

| **Test Statistics^a^** | | | | | | | | | | | | | | | | |
| --- | --- | --- | --- | --- | --- | --- | --- | --- | --- | --- | --- | --- | --- | --- | --- | --- |
|  | F3congruent conditions | F3incongruent conditions | F4congruent conditions | F4incongruent conditions | FZcongruent conditions | FZincongruent conditions | C3congruent conditions | C3incongruent conditions | C4congruent conditions | C4incongruent conditions | CZcongruent conditions | CZincongruent conditions | CP1congruent conditions | CP1incongruent conditions | CP2congruent conditions | CP2incongruent conditions |
| Mann-Whitney U | 148.000 | 158.000 | 173.000 | 174.000 | 164.000 | 166.000 | 172.000 | 169.000 | 172.000 | 170.000 | 177.000 | 176.000 | 159.000 | 177.000 | 180.000 | 172.000 |
| Wilcoxon W | 338.000 | 348.000 | 363.000 | 364.000 | 354.000 | 356.000 | 362.000 | 359.000 | 362.000 | 360.000 | 367.000 | 366.000 | 349.000 | 367.000 | 370.000 | 362.000 |
| Z | -.949 | -.657 | -.219 | -.190 | -.482 | -.423 | -.248 | -.336 | -.248 | -.307 | -.102 | -.131 | -.628 | -.102 | -.015 | -.248 |
| Asymp. Sig. (2-tailed) | .343 | .511 | .827 | .849 | .630 | .672 | .804 | .737 | .804 | .759 | .919 | .895 | .530 | .919 | .988 | .804 |
| Exact Sig. [2*(1-tailed Sig.)] | .354^b^ | .525^b^ | .840^b^ | .863^b^ | .644^b^ | .686^b^ | .817^b^ | .751^b^ | .817^b^ | .773^b^ | .931^b^ | .908^b^ | .544^b^ | .931^b^ | 1.000^b^ | .817^b^ |
| a. Grouping Variable: power | | | | | | | | | | | | | | | | |
| b. Not corrected for ties. | | | | | | | | | | | | | | | | |

**Only fearful faces in P1**

NPAR TESTS

/M-W= P7congruent conditions P7incongruent conditions P8congruent conditions P8incongruent conditions BY power(1 2)

/MISSING ANALYSIS.

**Mann-Whitney Test**

| **Ranks** | | | | |
| --- | --- | --- | --- | --- |
|  | power | N | Mean Rank | Sum of Ranks |
| P7congruent conditions | 1.00 | 19 | 16.95 | 322.00 |
|  | 2.00 | 19 | 22.05 | 419.00 |
|  | Total | 38 |  |  |
| P7incongruent conditions | 1.00 | 19 | 18.00 | 342.00 |
|  | 2.00 | 19 | 21.00 | 399.00 |
|  | Total | 38 |  |  |
| P8congruent conditions | 1.00 | 19 | 15.84 | 301.00 |
|  | 2.00 | 19 | 23.16 | 440.00 |
|  | Total | 38 |  |  |
| P8incongruent conditions | 1.00 | 19 | 16.58 | 315.00 |
|  | 2.00 | 19 | 22.42 | 426.00 |
|  | Total | 38 |  |  |

| **Test Statistics^a^** | | | | |
| --- | --- | --- | --- | --- |
|  | P7congruent conditions | P7incongruent conditions | P8congruent conditions | P8incongruent conditions |
| Mann-Whitney U | 132.000 | 152.000 | 111.000 | 125.000 |
| Wilcoxon W | 322.000 | 342.000 | 301.000 | 315.000 |
| Z | -1.416 | -.832 | -2.029 | -1.620 |
| Asymp. Sig. (2-tailed) | .157 | .405 | .042 | .105 |
| Exact Sig. [2*(1-tailed Sig.)] | .163^b^ | .418^b^ | .043^b^ | .109^b^ |
| a. Grouping Variable: power | | | | |
| b. Not corrected for ties. | | | | |

**Only fearful faces in N170**

NPAR TESTS

/M-W= P7congruent conditions P7incongruent conditions P8congruent conditions P8incongruent conditions BY power(1 2)

/MISSING ANALYSIS.

**Mann-Whitney Test**

| **Ranks** | | | | |
| --- | --- | --- | --- | --- |
|  | power | N | Mean Rank | Sum of Ranks |
| P7congruent conditions | 1.00 | 19 | 16.68 | 317.00 |
|  | 2.00 | 19 | 22.32 | 424.00 |
|  | Total | 38 |  |  |
| P7incongruent conditions | 1.00 | 19 | 17.68 | 336.00 |
|  | 2.00 | 19 | 21.32 | 405.00 |
|  | Total | 38 |  |  |
| P8congruent conditions | 1.00 | 19 | 16.37 | 311.00 |
|  | 2.00 | 19 | 22.63 | 430.00 |
|  | Total | 38 |  |  |
| P8incongruent conditions | 1.00 | 19 | 16.47 | 313.00 |
|  | 2.00 | 19 | 22.53 | 428.00 |
|  | Total | 38 |  |  |

| **Test Statistics^a^** | | | | |
| --- | --- | --- | --- | --- |
|  | P7congruent conditions | P7incongruent conditions | P8congruent conditions | P8incongruent conditions |
| Mann-Whitney U | 127.000 | 146.000 | 121.000 | 123.000 |
| Wilcoxon W | 317.000 | 336.000 | 311.000 | 313.000 |
| Z | -1.562 | -1.007 | -1.737 | -1.679 |
| Asymp. Sig. (2-tailed) | .118 | .314 | .082 | .093 |
| Exact Sig. [2*(1-tailed Sig.)] | .123^b^ | .325^b^ | .085^b^ | .096^b^ |
| a. Grouping Variable: power | | | | |
| b. Not corrected for ties. | | | | |

**Only fearful faces in N450**

NPAR TESTS

/M-W= F3congruent conditions F3incongruent conditions F4congruent conditions F4incongruent conditions FZcongruent conditions FZincongruent conditions C3congruent conditions C3incongruent conditions C4congruent conditions C4incongruent conditions CZcongruent conditions CZincongruent conditions CP1congruent conditions CP1incongruent conditions CP2congruent conditions CP2incongruent conditions BY power(1 2)

/MISSING ANALYSIS.

**Mann-Whitney Test**

| **Ranks** | | | | |
| --- | --- | --- | --- | --- |
|  | power | N | Mean Rank | Sum of Ranks |
| F3congruent conditions | 1.00 | 19 | 18.68 | 355.00 |
|  | 2.00 | 19 | 20.32 | 386.00 |
|  | Total | 38 |  |  |
| F3incongruent conditions | 1.00 | 19 | 18.84 | 358.00 |
|  | 2.00 | 19 | 20.16 | 383.00 |
|  | Total | 38 |  |  |
| F4congruent conditions | 1.00 | 19 | 20.21 | 384.00 |
|  | 2.00 | 19 | 18.79 | 357.00 |
|  | Total | 38 |  |  |
| F4incongruent conditions | 1.00 | 19 | 20.37 | 387.00 |
|  | 2.00 | 19 | 18.63 | 354.00 |
|  | Total | 38 |  |  |
| FZcongruent conditions | 1.00 | 19 | 19.42 | 369.00 |
|  | 2.00 | 19 | 19.58 | 372.00 |
|  | Total | 38 |  |  |
| FZincongruent conditions | 1.00 | 19 | 19.58 | 372.00 |
|  | 2.00 | 19 | 19.42 | 369.00 |
|  | Total | 38 |  |  |
| C3congruent conditions | 1.00 | 19 | 19.79 | 376.00 |
|  | 2.00 | 19 | 19.21 | 365.00 |
|  | Total | 38 |  |  |
| C3incongruent conditions | 1.00 | 19 | 19.74 | 375.00 |
|  | 2.00 | 19 | 19.26 | 366.00 |
|  | Total | 38 |  |  |
| C4congruent conditions | 1.00 | 19 | 19.79 | 376.00 |
|  | 2.00 | 19 | 19.21 | 365.00 |
|  | Total | 38 |  |  |
| C4incongruent conditions | 1.00 | 19 | 20.63 | 392.00 |
|  | 2.00 | 19 | 18.37 | 349.00 |
|  | Total | 38 |  |  |
| CZcongruent conditions | 1.00 | 19 | 19.84 | 377.00 |
|  | 2.00 | 19 | 19.16 | 364.00 |
|  | Total | 38 |  |  |
| CZincongruent conditions | 1.00 | 19 | 20.05 | 381.00 |
|  | 2.00 | 19 | 18.95 | 360.00 |
|  | Total | 38 |  |  |
| CP1congruent conditions | 1.00 | 19 | 18.58 | 353.00 |
|  | 2.00 | 19 | 20.42 | 388.00 |
|  | Total | 38 |  |  |
| CP1incongruent conditions | 1.00 | 19 | 19.84 | 377.00 |
|  | 2.00 | 19 | 19.16 | 364.00 |
|  | Total | 38 |  |  |
| CP2congruent conditions | 1.00 | 19 | 19.53 | 371.00 |
|  | 2.00 | 19 | 19.47 | 370.00 |
|  | Total | 38 |  |  |
| CP2incongruent conditions | 1.00 | 19 | 19.95 | 379.00 |
|  | 2.00 | 19 | 19.05 | 362.00 |
|  | Total | 38 |  |  |

| **Test Statistics^a^** | | | | | | | | | | | | | | | | |
| --- | --- | --- | --- | --- | --- | --- | --- | --- | --- | --- | --- | --- | --- | --- | --- | --- |
|  | F3congruent conditions | F3incongruent conditions | F4congruent conditions | F4incongruent conditions | FZcongruent conditions | FZincongruent conditions | C3congruent conditions | C3incongruent conditions | C4congruent conditions | C4incongruent conditions | CZcongruent conditions | CZincongruent conditions | CP1congruent conditions | CP1incongruent conditions | CP2congruent conditions | CP2incongruent conditions |
| Mann-Whitney U | 165.000 | 168.000 | 167.000 | 164.000 | 179.000 | 179.000 | 175.000 | 176.000 | 175.000 | 159.000 | 174.000 | 170.000 | 163.000 | 174.000 | 180.000 | 172.000 |
| Wilcoxon W | 355.000 | 358.000 | 357.000 | 354.000 | 369.000 | 369.000 | 365.000 | 366.000 | 365.000 | 349.000 | 364.000 | 360.000 | 353.000 | 364.000 | 370.000 | 362.000 |
| Z | -.453 | -.365 | -.394 | -.482 | -.044 | -.044 | -.161 | -.131 | -.161 | -.628 | -.190 | -.307 | -.511 | -.190 | -.015 | -.248 |
| Asymp. Sig. (2-tailed) | .651 | .715 | .693 | .630 | .965 | .965 | .872 | .895 | .872 | .530 | .849 | .759 | .609 | .849 | .988 | .804 |
| Exact Sig. [2*(1-tailed Sig.)] | .665^b^ | .729^b^ | .708^b^ | .644^b^ | .977^b^ | .977^b^ | .885^b^ | .908^b^ | .885^b^ | .544^b^ | .863^b^ | .773^b^ | .624^b^ | .863^b^ | 1.000^b^ | .817^b^ |
| a. Grouping Variable: power | | | | | | | | | | | | | | | | |
| b. Not corrected for ties. | | | | | | | | | | | | | | | | |

**Only happy faces in P1**

NPAR TESTS

/M-W= P7congruent conditions P7incongruent conditions P8congruent conditions P8incongruent conditions BY power(1 2)

/MISSING ANALYSIS.

**Mann-Whitney Test**

| **Ranks** | | | | |
| --- | --- | --- | --- | --- |
|  | power | N | Mean Rank | Sum of Ranks |
| P7congruent conditions | 1.00 | 19 | 16.79 | 319.00 |
|  | 2.00 | 19 | 22.21 | 422.00 |
|  | Total | 38 |  |  |
| P7incongruent conditions | 1.00 | 19 | 16.32 | 310.00 |
|  | 2.00 | 19 | 22.68 | 431.00 |
|  | Total | 38 |  |  |
| P8congruent conditions | 1.00 | 19 | 16.47 | 313.00 |
|  | 2.00 | 19 | 22.53 | 428.00 |
|  | Total | 38 |  |  |
| P8incongruent conditions | 1.00 | 19 | 16.53 | 314.00 |
|  | 2.00 | 19 | 22.47 | 427.00 |
|  | Total | 38 |  |  |

| **Test Statistics^a^** | | | | |
| --- | --- | --- | --- | --- |
|  | P7congruent conditions | P7incongruent conditions | P8congruent conditions | P8incongruent conditions |
| Mann-Whitney U | 129.000 | 120.000 | 123.000 | 124.000 |
| Wilcoxon W | 319.000 | 310.000 | 313.000 | 314.000 |
| Z | -1.504 | -1.766 | -1.679 | -1.650 |
| Asymp. Sig. (2-tailed) | .133 | .077 | .093 | .099 |
| Exact Sig. [2*(1-tailed Sig.)] | .138^b^ | .080^b^ | .096^b^ | .103^b^ |
| a. Grouping Variable: power | | | | |
| b. Not corrected for ties. | | | | |

**Only happy faces in N170**

NPAR TESTS

/M-W= P7congruent conditions P7incongruent conditions P8congruent conditions P8incongruent conditions BY power(1 2)

/MISSING ANALYSIS.

**Mann-Whitney Test**

| **Ranks** | | | | |
| --- | --- | --- | --- | --- |
|  | power | N | Mean Rank | Sum of Ranks |
| P7congruent conditions | 1.00 | 19 | 17.79 | 338.00 |
|  | 2.00 | 19 | 21.21 | 403.00 |
|  | Total | 38 |  |  |
| P7incongruent conditions | 1.00 | 19 | 16.84 | 320.00 |
|  | 2.00 | 19 | 22.16 | 421.00 |
|  | Total | 38 |  |  |
| P8congruent conditions | 1.00 | 19 | 17.11 | 325.00 |
|  | 2.00 | 19 | 21.89 | 416.00 |
|  | Total | 38 |  |  |
| P8incongruent conditions | 1.00 | 19 | 16.84 | 320.00 |
|  | 2.00 | 19 | 22.16 | 421.00 |
|  | Total | 38 |  |  |

| **Test Statistics^a^** | | | | |
| --- | --- | --- | --- | --- |
|  | P7congruent conditions | P7incongruent conditions | P8congruent conditions | P8incongruent conditions |
| Mann-Whitney U | 148.000 | 130.000 | 135.000 | 130.000 |
| Wilcoxon W | 338.000 | 320.000 | 325.000 | 320.000 |
| Z | -.949 | -1.474 | -1.328 | -1.474 |
| Asymp. Sig. (2-tailed) | .343 | .140 | .184 | .140 |
| Exact Sig. [2*(1-tailed Sig.)] | .354^b^ | .146^b^ | .191^b^ | .146^b^ |
| a. Grouping Variable: power | | | | |
| b. Not corrected for ties. | | | | |

**Only happy faces in N450**

NPAR TESTS

/M-W= F3congruent conditions F3incongruent conditions F4congruent conditions F4incongruent conditions FZcongruent conditions FZincongruent conditions C3congruent conditions C3incongruent conditions C4congruent conditions C4incongruent conditions CZcongruent conditions CZincongruent conditions CP1congruent conditions CP1incongruent conditions CP2congruent conditions CP2incongruent conditions BY power(1 2)

/MISSING ANALYSIS.

**Mann-Whitney Test**

| **Ranks** | | | | |
| --- | --- | --- | --- | --- |
|  | power | N | Mean Rank | Sum of Ranks |
| F3congruent conditions | 1.00 | 19 | 16.74 | 318.00 |
|  | 2.00 | 19 | 22.26 | 423.00 |
|  | Total | 38 |  |  |
| F3incongruent conditions | 1.00 | 19 | 17.84 | 339.00 |
|  | 2.00 | 19 | 21.16 | 402.00 |
|  | Total | 38 |  |  |
| F4congruent conditions | 1.00 | 19 | 19.53 | 371.00 |
|  | 2.00 | 19 | 19.47 | 370.00 |
|  | Total | 38 |  |  |
| F4incongruent conditions | 1.00 | 19 | 19.32 | 367.00 |
|  | 2.00 | 19 | 19.68 | 374.00 |
|  | Total | 38 |  |  |
| FZcongruent conditions | 1.00 | 19 | 18.47 | 351.00 |
|  | 2.00 | 19 | 20.53 | 390.00 |
|  | Total | 38 |  |  |
| FZincongruent conditions | 1.00 | 19 | 18.32 | 348.00 |
|  | 2.00 | 19 | 20.68 | 393.00 |
|  | Total | 38 |  |  |
| C3congruent conditions | 1.00 | 19 | 18.68 | 355.00 |
|  | 2.00 | 19 | 20.32 | 386.00 |
|  | Total | 38 |  |  |
| C3incongruent conditions | 1.00 | 19 | 19.32 | 367.00 |
|  | 2.00 | 19 | 19.68 | 374.00 |
|  | Total | 38 |  |  |
| C4congruent conditions | 1.00 | 19 | 19.63 | 373.00 |
|  | 2.00 | 19 | 19.37 | 368.00 |
|  | Total | 38 |  |  |
| C4incongruent conditions | 1.00 | 19 | 19.42 | 369.00 |
|  | 2.00 | 19 | 19.58 | 372.00 |
|  | Total | 38 |  |  |
| CZcongruent conditions | 1.00 | 19 | 18.95 | 360.00 |
|  | 2.00 | 19 | 20.05 | 381.00 |
|  | Total | 38 |  |  |
| CZincongruent conditions | 1.00 | 19 | 19.32 | 367.00 |
|  | 2.00 | 19 | 19.68 | 374.00 |
|  | Total | 38 |  |  |
| CP1congruent conditions | 1.00 | 19 | 17.89 | 340.00 |
|  | 2.00 | 19 | 21.11 | 401.00 |
|  | Total | 38 |  |  |
| CP1incongruent conditions | 1.00 | 19 | 19.00 | 361.00 |
|  | 2.00 | 19 | 20.00 | 380.00 |
|  | Total | 38 |  |  |
| CP2congruent conditions | 1.00 | 19 | 18.79 | 357.00 |
|  | 2.00 | 19 | 20.21 | 384.00 |
|  | Total | 38 |  |  |
| CP2incongruent conditions | 1.00 | 19 | 19.79 | 376.00 |
|  | 2.00 | 19 | 19.21 | 365.00 |
|  | Total | 38 |  |  |

| **Test Statistics^a^** | | | | | | | | | | | | | | | | |
| --- | --- | --- | --- | --- | --- | --- | --- | --- | --- | --- | --- | --- | --- | --- | --- | --- |
|  | F3congruent conditions | F3 incongruent conditions | F4congruent conditions | F4 incongruent conditions | FZcongruent conditions | FZincongruent conditions | C3congruent conditions | C3incongruent conditions | C4congruent conditions | C4incongruent conditions | CZcongruent conditions | CZincongruent conditions | CP1congruent conditions | CP1incongruent conditions | CP2congruent conditions | CP2incongruent conditions |
| Mann-Whitney U | 128.000 | 149.000 | 180.000 | 177.000 | 161.000 | 158.000 | 165.000 | 177.000 | 178.000 | 179.000 | 170.000 | 177.000 | 150.000 | 171.000 | 167.000 | 175.000 |
| Wilcoxon W | 318.000 | 339.000 | 370.000 | 367.000 | 351.000 | 348.000 | 355.000 | 367.000 | 368.000 | 369.000 | 360.000 | 367.000 | 340.000 | 361.000 | 357.000 | 365.000 |
| Z | -1.533 | -.920 | -.015 | -.102 | -.569 | -.657 | -.453 | -.102 | -.073 | -.044 | -.307 | -.102 | -.890 | -.277 | -.394 | -.161 |
| Asymp. Sig. (2-tailed) | .125 | .358 | .988 | .919 | .569 | .511 | .651 | .919 | .942 | .965 | .759 | .919 | .373 | .782 | .693 | .872 |
| Exact Sig. [2*(1-tailed Sig.)] | .130^b^ | .370^b^ | 1.000^b^ | .931^b^ | .583^b^ | .525^b^ | .665^b^ | .931^b^ | .954^b^ | .977^b^ | .773^b^ | .931^b^ | .385^b^ | .795^b^ | .708^b^ | .885^b^ |
| a. Grouping Variable: power | | | | | | | | | | | | | | | | |
| b. Not corrected for ties. | | | | | | | | | | | | | | | | |
