# Supplementary material for: The influence of social power on neural responses to emotional conflict
Source: PeerJ. 2021 Apr 12;9:e11267. doi: 10.7717/peerj.11267 (PMC8048403; doi:10.7717/peerj.11267)
Supplement: Supplemental Information 3 [file peerj-09-11267-s003.doc]

**Power manipulation checks**

T-TEST GROUPS=power(1 2)

/MISSING=ANALYSIS

/VARIABLES=power_manipulation_score

/CRITERIA=CI(.95).

**T-Test**

| **Notes** | | |
| --- | --- | --- |
| Output Created | | 02-JAN-2021 18:46:27 |
| Comments | |  |
| Input | Data | E:\11.17 \Power manipulation.sav |
|  | Active Dataset | Dataset |
|  | Filter | <none> |
|  | Weight | <none> |
|  | Split File | <none> |
|  | N of Rows in Working Data File | 41 |
| Missing Value Handling | Definition of Missing | User defined missing values are treated as missing. |
|  | Cases Used | Statistics for each analysis are based on the cases with no missing or out-of-range data for any variable in the analysis. |
| Syntax | | T-TEST GROUPS=power(1 2)  /MISSING=ANALYSIS  /VARIABLES=power_manipulation_score  /CRITERIA=CI(.95). |
| Resources | Processor Time | 00:00:00.02 |
|  | Elapsed Time | 00:00:00.03 |

| **Group Statistics** | | | | | |
| --- | --- | --- | --- | --- | --- |
|  | power | N | Mean | Std. Deviation | Std. Error Mean |
| power_manipulation_score | 1.00 | 19 | 4.0263 | .93502 | .21451 |
|  | 2.00 | 19 | 4.7368 | .97708 | .22416 |

| **Independent Samples Test** | | | | | | | | | | |
| --- | --- | --- | --- | --- | --- | --- | --- | --- | --- | --- |
|  | | Levene's Test for Equality of Variances | | t-test for Equality of Means | | | | | | |
|  |  | F | Sig. | t | df | Sig. (2-tailed) | Mean Difference | Std. Error Difference | 95% Confidence Interval of the Difference | |
|  |  |  |  |  |  |  |  |  | Lower | Upper |
| power_manipulation_score | Equal variances assumed | .134 | .716 | -2.290 | 36 | .028 | -.71053 | .31026 | -1.33976 | -.08129 |
|  | Equal variances not assumed |  |  | -2.290 | 35.931 | .028 | -.71053 | .31026 | -1.33980 | -.08125 |

**Self-control ability**

T-TEST GROUPS=power(1 2)

/MISSING=ANALYSIS

/VARIABLES=cognitive_emotion_regulation_score

/CRITERIA=CI(.95).

**T-Test**

| **Notes** | | |
| --- | --- | --- |
| Output Created | | 02-JAN-2021 01:19:48 |
| Comments | |  |
| Input | Data | E:\11.17 \raw_data.sav |
|  | Active Dataset | Dataset |
|  | Filter | <none> |
|  | Weight | <none> |
|  | Split File | <none> |
|  | N of Rows in Working Data File | 41 |
| Missing Value Handling | Definition of Missing | User defined missing values are treated as missing. |
|  | Cases Used | Statistics for each analysis are based on the cases with no missing or out-of-range data for any variable in the analysis. |
| Syntax | | T-TEST GROUPS=power(1 2)  /MISSING=ANALYSIS  /VARIABLES=cognitive_emotion_regulation_score  /CRITERIA=CI(.95). |
| Resources | Processor Time | 00:00:00.02 |
|  | Elapsed Time | 00:00:00.02 |

| **Group Statistics** | | | | | |
| --- | --- | --- | --- | --- | --- |
|  | power | N | Mean | Std. Deviation | Std. Error Mean |
| cognitive_emotion_regulation_score | 1.00 | 19 | 3.0965 | .21879 | .05019 |
|  | 2.00 | 19 | 3.1579 | .15440 | .03542 |

| **Independent Samples Test** | | | | | | | | | | |
| --- | --- | --- | --- | --- | --- | --- | --- | --- | --- | --- |
|  | | Levene's Test for Equality of Variances | | t-test for Equality of Means | | | | | | |
|  |  | F | Sig. | t | df | Sig. (2-tailed) | Mean Difference | Std. Error Difference | 95% Confidence Interval of the Difference | |
|  |  |  |  |  |  |  |  |  | Lower | Upper |
| cognitive_emotion_regulation_score | Equal variances assumed | 1.375 | .249 | -.999 | 36 | .324 | -.06140 | .06143 | -.18600 | .06319 |
|  | Equal variances not assumed |  |  | -.999 | 32.365 | .325 | -.06140 | .06143 | -.18649 | .06368 |

**All the faces in RT**

GLM Fear congruent Fear incongruent Happy congruent Happy incongruent BY power

/WSFACTOR=emotion 2 Polynomial conflict 2 Polynomial

/METHOD=SSTYPE(3)

/EMMEANS=TABLES(power) COMPARE ADJ(LSD)

/EMMEANS=TABLES(emotion) COMPARE ADJ(LSD)

/EMMEANS=TABLES(conflict) COMPARE ADJ(LSD)

/EMMEANS=TABLES(power*emotion)

/EMMEANS=TABLES(power*conflict)

/EMMEANS=TABLES(emotion*conflict)

/EMMEANS=TABLES(power*emotion*conflict)

/PRINT=DESCRIPTIVE ETASQ OPOWER HOMOGENEITY

/CRITERIA=ALPHA(.05)

/WSDESIGN=emotion conflict emotion*conflict

/DESIGN=power.

**General Linear Model**

| **Notes** | | |
| --- | --- | --- |
| Output Created | | 02-JAN-2021 16:17:06 |
| Comments | |  |
| Input | Data | E:\11.17 \ data\RT data.sav |
|  | Active Dataset | Dataset |
|  | Filter | <none> |
|  | Weight | <none> |
|  | Split File | <none> |
|  | N of Rows in Working Data File | 41 |
| Missing Value Handling | Definition of Missing | User-defined missing values are treated as missing. |
|  | Cases Used | Statistics are based on all cases with valid data for all variables in the model. |
| Syntax | | GLM Fear congruent Fear incongruent Happy congruent Happy incongruent BY power  /WSFACTOR=emotion 2 Polynomial conflict 2 Polynomial  /METHOD=SSTYPE(3)  /EMMEANS=TABLES(power) COMPARE ADJ(LSD)  /EMMEANS=TABLES(emotion) COMPARE ADJ(LSD)  /EMMEANS=TABLES(conflict) COMPARE ADJ(LSD)  /EMMEANS=TABLES(power*emotion)  /EMMEANS=TABLES(power*conflict)  /EMMEANS=TABLES(emotion*conflict)  /EMMEANS=TABLES(power*emotion*conflict)  /PRINT=DESCRIPTIVE ETASQ OPOWER HOMOGENEITY  /CRITERIA=ALPHA(.05)  /WSDESIGN=emotion conflict emotion*conflict  /DESIGN=power. |
| Resources | Processor Time | 00:00:00.08 |
|  | Elapsed Time | 00:00:00.08 |

| **Within-Subjects Factors** | | |
| --- | --- | --- |
| Measure: MEASURE_1 | | |
| emotion | conflict | Dependent Variable |
| 1 | 1 | Fear congruent |
|  | 2 | Fear incongruent |
| 2 | 1 | Happy congruent |
|  | 2 | Happy incongruent |

| **Between-Subjects Factors** | | |
| --- | --- | --- |
|  | | N |
| power | 1.00 | 19 |
|  | 2.00 | 19 |

| **Descriptive Statistics** | | | | |
| --- | --- | --- | --- | --- |
|  | power | Mean | Std. Deviation | N |
| Fear congruent | 1.00 | 677.8798 | 55.55548 | 19 |
|  | 2.00 | 673.0344 | 51.97556 | 19 |
|  | Total | 675.4571 | 53.12014 | 38 |
| Fear incongruent | 1.00 | 710.8455 | 67.63623 | 19 |
|  | 2.00 | 703.0406 | 51.94977 | 19 |
|  | Total | 706.9430 | 59.61602 | 38 |
| Happy congruent | 1.00 | 667.6963 | 60.70666 | 19 |
|  | 2.00 | 650.0428 | 57.08268 | 19 |
|  | Total | 658.8695 | 58.80518 | 38 |
| Happy incongruent | 1.00 | 705.9247 | 72.63114 | 19 |
|  | 2.00 | 671.8950 | 74.49370 | 19 |
|  | Total | 688.9098 | 74.58786 | 38 |

| **Box's Test of Equality of Covariance Matrices^a^** | |
| --- | --- |
| Box's M | 18.235 |
| F | 1.603 |
| df1 | 10 |
| df2 | 6196.016 |
| Sig. | .099 |
| Tests the null hypothesis that the observed covariance matrices of the dependent variables are equal across groups. | |
| a. Design: + power  Within Subjects Design: emotion + conflict + emotion * conflict | |

| **Multivariate Tests^a^** | | | | | | | | | |
| --- | --- | --- | --- | --- | --- | --- | --- | --- | --- |
| Effect | | Value | F | Hypothesis df | Error df | Sig. | Partial Eta Squared | Noncent. Parameter | Observed Power^c^ |
| emotion | Pillai's Trace | .111 | 4.473^b^ | 1.000 | 36.000 | .041 | .111 | 4.473 | .539 |
|  | Wilks' Lambda | .889 | 4.473^b^ | 1.000 | 36.000 | .041 | .111 | 4.473 | .539 |
|  | Hotelling's Trace | .124 | 4.473^b^ | 1.000 | 36.000 | .041 | .111 | 4.473 | .539 |
|  | Roy's Largest Root | .124 | 4.473^b^ | 1.000 | 36.000 | .041 | .111 | 4.473 | .539 |
| emotion * power | Pillai's Trace | .038 | 1.421^b^ | 1.000 | 36.000 | .241 | .038 | 1.421 | .213 |
|  | Wilks' Lambda | .962 | 1.421^b^ | 1.000 | 36.000 | .241 | .038 | 1.421 | .213 |
|  | Hotelling's Trace | .039 | 1.421^b^ | 1.000 | 36.000 | .241 | .038 | 1.421 | .213 |
|  | Roy's Largest Root | .039 | 1.421^b^ | 1.000 | 36.000 | .241 | .038 | 1.421 | .213 |
| conflict | Pillai's Trace | .601 | 54.238^b^ | 1.000 | 36.000 | .000 | .601 | 54.238 | 1.000 |
|  | Wilks' Lambda | .399 | 54.238^b^ | 1.000 | 36.000 | .000 | .601 | 54.238 | 1.000 |
|  | Hotelling's Trace | 1.507 | 54.238^b^ | 1.000 | 36.000 | .000 | .601 | 54.238 | 1.000 |
|  | Roy's Largest Root | 1.507 | 54.238^b^ | 1.000 | 36.000 | .000 | .601 | 54.238 | 1.000 |
| conflict * power | Pillai's Trace | .036 | 1.339^b^ | 1.000 | 36.000 | .255 | .036 | 1.339 | .203 |
|  | Wilks' Lambda | .964 | 1.339^b^ | 1.000 | 36.000 | .255 | .036 | 1.339 | .203 |
|  | Hotelling's Trace | .037 | 1.339^b^ | 1.000 | 36.000 | .255 | .036 | 1.339 | .203 |
|  | Roy's Largest Root | .037 | 1.339^b^ | 1.000 | 36.000 | .255 | .036 | 1.339 | .203 |
| emotion * conflict | Pillai's Trace | .003 | .103^b^ | 1.000 | 36.000 | .750 | .003 | .103 | .061 |
|  | Wilks' Lambda | .997 | .103^b^ | 1.000 | 36.000 | .750 | .003 | .103 | .061 |
|  | Hotelling's Trace | .003 | .103^b^ | 1.000 | 36.000 | .750 | .003 | .103 | .061 |
|  | Roy's Largest Root | .003 | .103^b^ | 1.000 | 36.000 | .750 | .003 | .103 | .061 |
| emotion * conflict * power | Pillai's Trace | .058 | 2.219^b^ | 1.000 | 36.000 | .145 | .058 | 2.219 | .305 |
|  | Wilks' Lambda | .942 | 2.219^b^ | 1.000 | 36.000 | .145 | .058 | 2.219 | .305 |
|  | Hotelling's Trace | .062 | 2.219^b^ | 1.000 | 36.000 | .145 | .058 | 2.219 | .305 |
|  | Roy's Largest Root | .062 | 2.219^b^ | 1.000 | 36.000 | .145 | .058 | 2.219 | .305 |
| a. Design: + power  Within Subjects Design: emotion + conflict + emotion * conflict | | | | | | | | | |
| b. Exact statistic | | | | | | | | | |
| c. Computed using alpha = .05 | | | | | | | | | |

| **Mauchly's Test of Sphericity^a^** | | | | | | | |
| --- | --- | --- | --- | --- | --- | --- | --- |
| Measure: MEASURE_1 | | | | | | | |
| Within Subjects Effect | Mauchly's W | Approx. Chi-Square | df | Sig. | Epsilon^b^ | | |
|  |  |  |  |  | Greenhouse-Geisser | Huynh-Feldt | Lower-bound |
| emotion | 1.000 | .000 | 0 | . | 1.000 | 1.000 | 1.000 |
| conflict | 1.000 | .000 | 0 | . | 1.000 | 1.000 | 1.000 |
| emotion * conflict | 1.000 | .000 | 0 | . | 1.000 | 1.000 | 1.000 |
| Tests the null hypothesis that the error covariance matrix of the orthonormalized transformed dependent variables is proportional to an identity matrix. | | | | | | | |
| a. Design: + power  Within Subjects Design: emotion + conflict + emotion * conflict | | | | | | | |
| b. May be used to adjust the degrees of freedom for the averaged tests of significance. Corrected tests are displayed in the Tests of Within-Subjects Effects table. | | | | | | | |

| **Tests of Within-Subjects Effects** | | | | | | | | | |
| --- | --- | --- | --- | --- | --- | --- | --- | --- | --- |
| Measure: MEASURE_1 | | | | | | | | | |
| Source | | Type III Sum of Squares | df | Mean Square | F | Sig. | Partial Eta Squared | Noncent. Parameter | Observed Power^a^ |
| emotion | Sphericity Assumed | 11386.653 | 1 | 11386.653 | 4.473 | .041 | .111 | 4.473 | .539 |
|  | Greenhouse-Geisser | 11386.653 | 1.000 | 11386.653 | 4.473 | .041 | .111 | 4.473 | .539 |
|  | Huynh-Feldt | 11386.653 | 1.000 | 11386.653 | 4.473 | .041 | .111 | 4.473 | .539 |
|  | Lower-bound | 11386.653 | 1.000 | 11386.653 | 4.473 | .041 | .111 | 4.473 | .539 |
| emotion * power | Sphericity Assumed | 3618.482 | 1 | 3618.482 | 1.421 | .241 | .038 | 1.421 | .213 |
|  | Greenhouse-Geisser | 3618.482 | 1.000 | 3618.482 | 1.421 | .241 | .038 | 1.421 | .213 |
|  | Huynh-Feldt | 3618.482 | 1.000 | 3618.482 | 1.421 | .241 | .038 | 1.421 | .213 |
|  | Lower-bound | 3618.482 | 1.000 | 3618.482 | 1.421 | .241 | .038 | 1.421 | .213 |
| Error(emotion) | Sphericity Assumed | 91644.802 | 36 | 2545.689 |  |  |  |  |  |
|  | Greenhouse-Geisser | 91644.802 | 36.000 | 2545.689 |  |  |  |  |  |
|  | Huynh-Feldt | 91644.802 | 36.000 | 2545.689 |  |  |  |  |  |
|  | Lower-bound | 91644.802 | 36.000 | 2545.689 |  |  |  |  |  |
| conflict | Sphericity Assumed | 35961.997 | 1 | 35961.997 | 54.238 | .000 | .601 | 54.238 | 1.000 |
|  | Greenhouse-Geisser | 35961.997 | 1.000 | 35961.997 | 54.238 | .000 | .601 | 54.238 | 1.000 |
|  | Huynh-Feldt | 35961.997 | 1.000 | 35961.997 | 54.238 | .000 | .601 | 54.238 | 1.000 |
|  | Lower-bound | 35961.997 | 1.000 | 35961.997 | 54.238 | .000 | .601 | 54.238 | 1.000 |
| conflict * power | Sphericity Assumed | 887.937 | 1 | 887.937 | 1.339 | .255 | .036 | 1.339 | .203 |
|  | Greenhouse-Geisser | 887.937 | 1.000 | 887.937 | 1.339 | .255 | .036 | 1.339 | .203 |
|  | Huynh-Feldt | 887.937 | 1.000 | 887.937 | 1.339 | .255 | .036 | 1.339 | .203 |
|  | Lower-bound | 887.937 | 1.000 | 887.937 | 1.339 | .255 | .036 | 1.339 | .203 |
| Error(conflict) | Sphericity Assumed | 23869.649 | 36 | 663.046 |  |  |  |  |  |
|  | Greenhouse-Geisser | 23869.649 | 36.000 | 663.046 |  |  |  |  |  |
|  | Huynh-Feldt | 23869.649 | 36.000 | 663.046 |  |  |  |  |  |
|  | Lower-bound | 23869.649 | 36.000 | 663.046 |  |  |  |  |  |
| emotion * conflict | Sphericity Assumed | 19.854 | 1 | 19.854 | .103 | .750 | .003 | .103 | .061 |
|  | Greenhouse-Geisser | 19.854 | 1.000 | 19.854 | .103 | .750 | .003 | .103 | .061 |
|  | Huynh-Feldt | 19.854 | 1.000 | 19.854 | .103 | .750 | .003 | .103 | .061 |
|  | Lower-bound | 19.854 | 1.000 | 19.854 | .103 | .750 | .003 | .103 | .061 |
| emotion * conflict * power | Sphericity Assumed | 427.520 | 1 | 427.520 | 2.219 | .145 | .058 | 2.219 | .305 |
|  | Greenhouse-Geisser | 427.520 | 1.000 | 427.520 | 2.219 | .145 | .058 | 2.219 | .305 |
|  | Huynh-Feldt | 427.520 | 1.000 | 427.520 | 2.219 | .145 | .058 | 2.219 | .305 |
|  | Lower-bound | 427.520 | 1.000 | 427.520 | 2.219 | .145 | .058 | 2.219 | .305 |
| Error(emotion*conflict) | Sphericity Assumed | 6935.360 | 36 | 192.649 |  |  |  |  |  |
|  | Greenhouse-Geisser | 6935.360 | 36.000 | 192.649 |  |  |  |  |  |
|  | Huynh-Feldt | 6935.360 | 36.000 | 192.649 |  |  |  |  |  |
|  | Lower-bound | 6935.360 | 36.000 | 192.649 |  |  |  |  |  |
| a. Computed using alpha = .05 | | | | | | | | | |

| **Tests of Within-Subjects Contrasts** | | | | | | | | | | |
| --- | --- | --- | --- | --- | --- | --- | --- | --- | --- | --- |
| Measure: MEASURE_1 | | | | | | | | | | |
| Source | emotion | conflict | Type III Sum of Squares | df | Mean Square | F | Sig. | Partial Eta Squared | Noncent. Parameter | Observed Power^a^ |
| emotion | Linear |  | 11386.653 | 1 | 11386.653 | 4.473 | .041 | .111 | 4.473 | .539 |
| emotion * power | Linear |  | 3618.482 | 1 | 3618.482 | 1.421 | .241 | .038 | 1.421 | .213 |
| Error(emotion) | Linear |  | 91644.802 | 36 | 2545.689 |  |  |  |  |  |
| conflict |  | Linear | 35961.997 | 1 | 35961.997 | 54.238 | .000 | .601 | 54.238 | 1.000 |
| conflict * power |  | Linear | 887.937 | 1 | 887.937 | 1.339 | .255 | .036 | 1.339 | .203 |
| Error(conflict) |  | Linear | 23869.649 | 36 | 663.046 |  |  |  |  |  |
| emotion * conflict | Linear | Linear | 19.854 | 1 | 19.854 | .103 | .750 | .003 | .103 | .061 |
| emotion * conflict * power | Linear | Linear | 427.520 | 1 | 427.520 | 2.219 | .145 | .058 | 2.219 | .305 |
| Error(emotion*conflict) | Linear | Linear | 6935.360 | 36 | 192.649 |  |  |  |  |  |
| a. Computed using alpha = .05 | | | | | | | | | | |

| **Levene's Test of Equality of Error Variances^a^** | | | | |
| --- | --- | --- | --- | --- |
|  | F | df1 | df2 | Sig. |
| Fear congruent | .004 | 1 | 36 | .947 |
| Fear incongruent | 1.469 | 1 | 36 | .233 |
| Happy congruent | .073 | 1 | 36 | .789 |
| Happy incongruent | .000 | 1 | 36 | .987 |
| Tests the null hypothesis that the error variance of the dependent variable is equal across groups. | | | | |
| a. Design: + power  Within Subjects Design: emotion + conflict + emotion * conflict | | | | |

| **Tests of Between-Subjects Effects** | | | | | | | | |
| --- | --- | --- | --- | --- | --- | --- | --- | --- |
| Measure: MEASURE_1 | | | | | | | | |
| Transformed Variable: Average | | | | | | | | |
| Source | Type III Sum of Squares | df | Mean Square | F | Sig. | Partial Eta Squared | Noncent. Parameter | Observed Power^a^ |
|  | 70811860.197 | 1 | 70811860.197 | 5894.390 | .000 | .994 | 5894.390 | 1.000 |
| power | 9829.677 | 1 | 9829.677 | .818 | .372 | .022 | .818 | .142 |
| Error | 432483.584 | 36 | 12013.433 |  |  |  |  |  |
| a. Computed using alpha = .05 | | | | | | | | |

**Estimated Marginal Means**

**1. power**

| **Estimates** | | | | |
| --- | --- | --- | --- | --- |
| Measure: MEASURE_1 | | | | |
| power | Mean | Std. Error | 95% Confidence Interval | |
|  |  |  | Lower Bound | Upper Bound |
| 1.00 | 690.587 | 12.573 | 665.088 | 716.085 |
| 2.00 | 674.503 | 12.573 | 649.005 | 700.002 |

| **Pairwise Comparisons** | | | | | | |
| --- | --- | --- | --- | --- | --- | --- |
| Measure: MEASURE_1 | | | | | | |
| (I) power | (J) power | Mean Difference (I-J) | Std. Error | Sig.^a^ | 95% Confidence Interval for Difference^a^ | |
|  |  |  |  |  | Lower Bound | Upper Bound |
| 1.00 | 2.00 | 16.083 | 17.780 | .372 | -19.977 | 52.144 |
| 2.00 | 1.00 | -16.083 | 17.780 | .372 | -52.144 | 19.977 |
| Based on estimated marginal means | | | | | | |
| a. Adjustment for multiple comparisons: Least Significant Difference (equivalent to no adjustments). | | | | | | |

| **Univariate Tests** | | | | | | | | |
| --- | --- | --- | --- | --- | --- | --- | --- | --- |
| Measure: MEASURE_1 | | | | | | | | |
|  | Sum of Squares | df | Mean Square | F | Sig. | Partial Eta Squared | Noncent. Parameter | Observed Power^a^ |
| Contrast | 2457.419 | 1 | 2457.419 | .818 | .372 | .022 | .818 | .142 |
| Error | 108120.896 | 36 | 3003.358 |  |  |  |  |  |
| The F tests the effect of power. This test is based on the linearly independent pairwise comparisons among the estimated marginal means. | | | | | | | | |
| a. Computed using alpha = .05 | | | | | | | | |

**2. emotion**

| **Estimates** | | | | |
| --- | --- | --- | --- | --- |
| Measure: MEASURE_1 | | | | |
| emotion | Mean | Std. Error | 95% Confidence Interval | |
|  |  |  | Lower Bound | Upper Bound |
| 1 | 691.200 | 9.024 | 672.899 | 709.501 |
| 2 | 673.890 | 10.495 | 652.606 | 695.174 |

| **Pairwise Comparisons** | | | | | | |
| --- | --- | --- | --- | --- | --- | --- |
| Measure: MEASURE_1 | | | | | | |
| (I) emotion | (J) emotion | Mean Difference (I-J) | Std. Error | Sig.^b^ | 95% Confidence Interval for Difference^b^ | |
|  |  |  |  |  | Lower Bound | Upper Bound |
| 1 | 2 | 17.310^*^ | 8.185 | .041 | .711 | 33.910 |
| 2 | 1 | -17.310^*^ | 8.185 | .041 | -33.910 | -.711 |
| Based on estimated marginal means | | | | | | |
| *. The mean difference is significant at the .05 level. | | | | | | |
| b. Adjustment for multiple comparisons: Least Significant Difference (equivalent to no adjustments). | | | | | | |

| **Multivariate Tests** | | | | | | | | |
| --- | --- | --- | --- | --- | --- | --- | --- | --- |
|  | Value | F | Hypothesis df | Error df | Sig. | Partial Eta Squared | Noncent. Parameter | Observed Power^b^ |
| Pillai's trace | .111 | 4.473^a^ | 1.000 | 36.000 | .041 | .111 | 4.473 | .539 |
| Wilks' lambda | .889 | 4.473^a^ | 1.000 | 36.000 | .041 | .111 | 4.473 | .539 |
| Hotelling's trace | .124 | 4.473^a^ | 1.000 | 36.000 | .041 | .111 | 4.473 | .539 |
| Roy's largest root | .124 | 4.473^a^ | 1.000 | 36.000 | .041 | .111 | 4.473 | .539 |
| Each F tests the multivariate effect of emotion. These tests are based on the linearly independent pairwise comparisons among the estimated marginal means. | | | | | | | | |
| a. Exact statistic | | | | | | | | |
| b. Computed using alpha = .05 | | | | | | | | |

**3. conflict**

| **Estimates** | | | | |
| --- | --- | --- | --- | --- |
| Measure: MEASURE_1 | | | | |
| conflict | Mean | Std. Error | 95% Confidence Interval | |
|  |  |  | Lower Bound | Upper Bound |
| 1 | 667.163 | 8.317 | 650.296 | 684.031 |
| 2 | 697.926 | 9.881 | 677.888 | 717.965 |

| **Pairwise Comparisons** | | | | | | |
| --- | --- | --- | --- | --- | --- | --- |
| Measure: MEASURE_1 | | | | | | |
| (I) conflict | (J) conflict | Mean Difference (I-J) | Std. Error | Sig.^b^ | 95% Confidence Interval for Difference^b^ | |
|  |  |  |  |  | Lower Bound | Upper Bound |
| 1 | 2 | -30.763^*^ | 4.177 | .000 | -39.235 | -22.291 |
| 2 | 1 | 30.763^*^ | 4.177 | .000 | 22.291 | 39.235 |
| Based on estimated marginal means | | | | | | |
| *. The mean difference is significant at the .05 level. | | | | | | |
| b. Adjustment for multiple comparisons: Least Significant Difference (equivalent to no adjustments). | | | | | | |

| **Multivariate Tests** | | | | | | | | |
| --- | --- | --- | --- | --- | --- | --- | --- | --- |
|  | Value | F | Hypothesis df | Error df | Sig. | Partial Eta Squared | Noncent. Parameter | Observed Power^b^ |
| Pillai's trace | .601 | 54.238^a^ | 1.000 | 36.000 | .000 | .601 | 54.238 | 1.000 |
| Wilks' lambda | .399 | 54.238^a^ | 1.000 | 36.000 | .000 | .601 | 54.238 | 1.000 |
| Hotelling's trace | 1.507 | 54.238^a^ | 1.000 | 36.000 | .000 | .601 | 54.238 | 1.000 |
| Roy's largest root | 1.507 | 54.238^a^ | 1.000 | 36.000 | .000 | .601 | 54.238 | 1.000 |
| Each F tests the multivariate effect of conflict. These tests are based on the linearly independent pairwise comparisons among the estimated marginal means. | | | | | | | | |
| a. Exact statistic | | | | | | | | |
| b. Computed using alpha = .05 | | | | | | | | |

| **4. power * emotion** | | | | | |
| --- | --- | --- | --- | --- | --- |
| Measure: MEASURE_1 | | | | | |
| power | emotion | Mean | Std. Error | 95% Confidence Interval | |
|  |  |  |  | Lower Bound | Upper Bound |
| 1.00 | 1 | 694.363 | 12.762 | 668.481 | 720.245 |
|  | 2 | 686.811 | 14.842 | 656.710 | 716.911 |
| 2.00 | 1 | 688.037 | 12.762 | 662.155 | 713.920 |
|  | 2 | 660.969 | 14.842 | 630.869 | 691.069 |

| **5. power * conflict** | | | | | |
| --- | --- | --- | --- | --- | --- |
| Measure: MEASURE_1 | | | | | |
| power | conflict | Mean | Std. Error | 95% Confidence Interval | |
|  |  |  |  | Lower Bound | Upper Bound |
| 1.00 | 1 | 672.788 | 11.762 | 648.934 | 696.642 |
|  | 2 | 708.385 | 13.973 | 680.046 | 736.724 |
| 2.00 | 1 | 661.539 | 11.762 | 637.684 | 685.393 |
|  | 2 | 687.468 | 13.973 | 659.129 | 715.807 |

| **6. emotion * conflict** | | | | | |
| --- | --- | --- | --- | --- | --- |
| Measure: MEASURE_1 | | | | | |
| emotion | conflict | Mean | Std. Error | 95% Confidence Interval | |
|  |  |  |  | Lower Bound | Upper Bound |
| 1 | 1 | 675.457 | 8.727 | 657.758 | 693.156 |
|  | 2 | 706.943 | 9.783 | 687.103 | 726.783 |
| 2 | 1 | 658.870 | 9.558 | 639.484 | 678.255 |
|  | 2 | 688.910 | 11.934 | 664.706 | 713.114 |

| **7. power * emotion * conflict** | | | | | | |
| --- | --- | --- | --- | --- | --- | --- |
| Measure: MEASURE_1 | | | | | | |
| power | emotion | conflict | Mean | Std. Error | 95% Confidence Interval | |
|  |  |  |  |  | Lower Bound | Upper Bound |
| 1.00 | 1 | 1 | 677.880 | 12.341 | 652.850 | 702.910 |
|  |  | 2 | 710.845 | 13.835 | 682.787 | 738.904 |
|  | 2 | 1 | 667.696 | 13.518 | 640.281 | 695.112 |
|  |  | 2 | 705.925 | 16.878 | 671.695 | 740.154 |
| 2.00 | 1 | 1 | 673.034 | 12.341 | 648.005 | 698.064 |
|  |  | 2 | 703.041 | 13.835 | 674.982 | 731.099 |
|  | 2 | 1 | 650.043 | 13.518 | 622.627 | 677.458 |
|  |  | 2 | 671.895 | 16.878 | 637.665 | 706.125 |

**All the faces in ACC**

GLM Fear congruent Fear incongruent Happy congruent Happy incongruent BY power

/WSFACTOR=emotion 2 Polynomial conflict 2 Polynomial

/METHOD=SSTYPE(3)

/EMMEANS=TABLES(power) COMPARE ADJ(LSD)

/EMMEANS=TABLES(emotion) COMPARE ADJ(LSD)

/EMMEANS=TABLES(conflict) COMPARE ADJ(LSD)

/EMMEANS=TABLES(power*emotion)

/EMMEANS=TABLES(power*conflict)

/EMMEANS=TABLES(emotion*conflict)

/EMMEANS=TABLES(power*emotion*conflict)

/PRINT=DESCRIPTIVE ETASQ OPOWER HOMOGENEITY

/CRITERIA=ALPHA(.05)

/WSDESIGN=emotion conflict emotion*conflict

/DESIGN=power.

**General Linear Model**

| **Notes** | | |
| --- | --- | --- |
| Output Created | | 02-JAN-2021 16:18:35 |
| Comments | |  |
| Input | Data | E:\11.17 \ data\ACC data.sav |
|  | Active Dataset | Dataset |
|  | Filter | <none> |
|  | Weight | <none> |
|  | Split File | <none> |
|  | N of Rows in Working Data File | 41 |
| Missing Value Handling | Definition of Missing | User-defined missing values are treated as missing. |
|  | Cases Used | Statistics are based on all cases with valid data for all variables in the model. |
| Syntax | | GLM Fear congruent Fear incongruent Happy congruent Happy incongruent BY power  /WSFACTOR=emotion 2 Polynomial conflict 2 Polynomial  /METHOD=SSTYPE(3)  /EMMEANS=TABLES(power) COMPARE ADJ(LSD)  /EMMEANS=TABLES(emotion) COMPARE ADJ(LSD)  /EMMEANS=TABLES(conflict) COMPARE ADJ(LSD)  /EMMEANS=TABLES(power*emotion)  /EMMEANS=TABLES(power*conflict)  /EMMEANS=TABLES(emotion*conflict)  /EMMEANS=TABLES(power*emotion*conflict)  /PRINT=DESCRIPTIVE ETASQ OPOWER HOMOGENEITY  /CRITERIA=ALPHA(.05)  /WSDESIGN=emotion conflict emotion*conflict  /DESIGN=power. |
| Resources | Processor Time | 00:00:00.08 |
|  | Elapsed Time | 00:00:00.05 |

| **Within-Subjects Factors** | | |
| --- | --- | --- |
| Measure: MEASURE_1 | | |
| emotion | conflict | Dependent Variable |
| 1 | 1 | Fear congruent |
|  | 2 | Fear incongruent |
| 2 | 1 | Happy congruent |
|  | 2 | Happy incongruent |

| **Between-Subjects Factors** | | |
| --- | --- | --- |
|  | | N |
| power | 1.00 | 19 |
|  | 2.00 | 19 |

| **Descriptive Statistics** | | | | |
| --- | --- | --- | --- | --- |
|  | power | Mean | Std. Deviation | N |
| Fear congruent | 1.00 | .9289 | .05953 | 19 |
|  | 2.00 | .9168 | .09505 | 19 |
|  | Total | .9229 | .07846 | 38 |
| Fear incongruent | 1.00 | .8332 | .10451 | 19 |
|  | 2.00 | .8579 | .12090 | 19 |
|  | Total | .8455 | .11217 | 38 |
| Happy congruent | 1.00 | .9058 | .07515 | 19 |
|  | 2.00 | .8968 | .11294 | 19 |
|  | Total | .9013 | .09473 | 38 |
| Happy incongruent | 1.00 | .7942 | .15893 | 19 |
|  | 2.00 | .7995 | .15963 | 19 |
|  | Total | .7968 | .15714 | 38 |

| **Box's Test of Equality of Covariance Matrices^a^** | |
| --- | --- |
| Box's M | 18.242 |
| F | 1.603 |
| df1 | 10 |
| df2 | 6196.016 |
| Sig. | .099 |
| Tests the null hypothesis that the observed covariance matrices of the dependent variables are equal across groups. | |
| a. Design: + power  Within Subjects Design: emotion + conflict + emotion * conflict | |

| **Multivariate Tests^a^** | | | | | | | | | |
| --- | --- | --- | --- | --- | --- | --- | --- | --- | --- |
| Effect | | Value | F | Hypothesis df | Error df | Sig. | Partial Eta Squared | Noncent. Parameter | Observed Power^c^ |
| emotion | Pillai's Trace | .204 | 9.222^b^ | 1.000 | 36.000 | .004 | .204 | 9.222 | .840 |
|  | Wilks' Lambda | .796 | 9.222^b^ | 1.000 | 36.000 | .004 | .204 | 9.222 | .840 |
|  | Hotelling's Trace | .256 | 9.222^b^ | 1.000 | 36.000 | .004 | .204 | 9.222 | .840 |
|  | Roy's Largest Root | .256 | 9.222^b^ | 1.000 | 36.000 | .004 | .204 | 9.222 | .840 |
| emotion * power | Pillai's Trace | .003 | .124^b^ | 1.000 | 36.000 | .726 | .003 | .124 | .064 |
|  | Wilks' Lambda | .997 | .124^b^ | 1.000 | 36.000 | .726 | .003 | .124 | .064 |
|  | Hotelling's Trace | .003 | .124^b^ | 1.000 | 36.000 | .726 | .003 | .124 | .064 |
|  | Roy's Largest Root | .003 | .124^b^ | 1.000 | 36.000 | .726 | .003 | .124 | .064 |
| conflict | Pillai's Trace | .565 | 46.807^b^ | 1.000 | 36.000 | .000 | .565 | 46.807 | 1.000 |
|  | Wilks' Lambda | .435 | 46.807^b^ | 1.000 | 36.000 | .000 | .565 | 46.807 | 1.000 |
|  | Hotelling's Trace | 1.300 | 46.807^b^ | 1.000 | 36.000 | .000 | .565 | 46.807 | 1.000 |
|  | Roy's Largest Root | 1.300 | 46.807^b^ | 1.000 | 36.000 | .000 | .565 | 46.807 | 1.000 |
| conflict * power | Pillai's Trace | .025 | .922^b^ | 1.000 | 36.000 | .343 | .025 | .922 | .155 |
|  | Wilks' Lambda | .975 | .922^b^ | 1.000 | 36.000 | .343 | .025 | .922 | .155 |
|  | Hotelling's Trace | .026 | .922^b^ | 1.000 | 36.000 | .343 | .025 | .922 | .155 |
|  | Roy's Largest Root | .026 | .922^b^ | 1.000 | 36.000 | .343 | .025 | .922 | .155 |
| emotion * conflict | Pillai's Trace | .133 | 5.507^b^ | 1.000 | 36.000 | .025 | .133 | 5.507 | .627 |
|  | Wilks' Lambda | .867 | 5.507^b^ | 1.000 | 36.000 | .025 | .133 | 5.507 | .627 |
|  | Hotelling's Trace | .153 | 5.507^b^ | 1.000 | 36.000 | .025 | .133 | 5.507 | .627 |
|  | Roy's Largest Root | .153 | 5.507^b^ | 1.000 | 36.000 | .025 | .133 | 5.507 | .627 |
| emotion * conflict * power | Pillai's Trace | .026 | .960^b^ | 1.000 | 36.000 | .334 | .026 | .960 | .159 |
|  | Wilks' Lambda | .974 | .960^b^ | 1.000 | 36.000 | .334 | .026 | .960 | .159 |
|  | Hotelling's Trace | .027 | .960^b^ | 1.000 | 36.000 | .334 | .026 | .960 | .159 |
|  | Roy's Largest Root | .027 | .960^b^ | 1.000 | 36.000 | .334 | .026 | .960 | .159 |
| a. Design: + power  Within Subjects Design: emotion + conflict + emotion * conflict | | | | | | | | | |
| b. Exact statistic | | | | | | | | | |
| c. Computed using alpha = .05 | | | | | | | | | |

| **Mauchly's Test of Sphericity^a^** | | | | | | | |
| --- | --- | --- | --- | --- | --- | --- | --- |
| Measure: MEASURE_1 | | | | | | | |
| Within Subjects Effect | Mauchly's W | Approx. Chi-Square | df | Sig. | Epsilon^b^ | | |
|  |  |  |  |  | Greenhouse-Geisser | Huynh-Feldt | Lower-bound |
| emotion | 1.000 | .000 | 0 | . | 1.000 | 1.000 | 1.000 |
| conflict | 1.000 | .000 | 0 | . | 1.000 | 1.000 | 1.000 |
| emotion * conflict | 1.000 | .000 | 0 | . | 1.000 | 1.000 | 1.000 |
| Tests the null hypothesis that the error covariance matrix of the orthonormalized transformed dependent variables is proportional to an identity matrix. | | | | | | | |
| a. Design: + power  Within Subjects Design: emotion + conflict + emotion * conflict | | | | | | | |
| b. May be used to adjust the degrees of freedom for the averaged tests of significance. Corrected tests are displayed in the Tests of Within-Subjects Effects table. | | | | | | | |

| **Tests of Within-Subjects Effects** | | | | | | | | | |
| --- | --- | --- | --- | --- | --- | --- | --- | --- | --- |
| Measure: MEASURE_1 | | | | | | | | | |
| Source | | Type III Sum of Squares | df | Mean Square | F | Sig. | Partial Eta Squared | Noncent. Parameter | Observed Power^a^ |
| emotion | Sphericity Assumed | .047 | 1 | .047 | 9.222 | .004 | .204 | 9.222 | .840 |
|  | Greenhouse-Geisser | .047 | 1.000 | .047 | 9.222 | .004 | .204 | 9.222 | .840 |
|  | Huynh-Feldt | .047 | 1.000 | .047 | 9.222 | .004 | .204 | 9.222 | .840 |
|  | Lower-bound | .047 | 1.000 | .047 | 9.222 | .004 | .204 | 9.222 | .840 |
| emotion * power | Sphericity Assumed | .001 | 1 | .001 | .124 | .726 | .003 | .124 | .064 |
|  | Greenhouse-Geisser | .001 | 1.000 | .001 | .124 | .726 | .003 | .124 | .064 |
|  | Huynh-Feldt | .001 | 1.000 | .001 | .124 | .726 | .003 | .124 | .064 |
|  | Lower-bound | .001 | 1.000 | .001 | .124 | .726 | .003 | .124 | .064 |
| Error(emotion) | Sphericity Assumed | .183 | 36 | .005 |  |  |  |  |  |
|  | Greenhouse-Geisser | .183 | 36.000 | .005 |  |  |  |  |  |
|  | Huynh-Feldt | .183 | 36.000 | .005 |  |  |  |  |  |
|  | Lower-bound | .183 | 36.000 | .005 |  |  |  |  |  |
| conflict | Sphericity Assumed | .314 | 1 | .314 | 46.807 | .000 | .565 | 46.807 | 1.000 |
|  | Greenhouse-Geisser | .314 | 1.000 | .314 | 46.807 | .000 | .565 | 46.807 | 1.000 |
|  | Huynh-Feldt | .314 | 1.000 | .314 | 46.807 | .000 | .565 | 46.807 | 1.000 |
|  | Lower-bound | .314 | 1.000 | .314 | 46.807 | .000 | .565 | 46.807 | 1.000 |
| conflict * power | Sphericity Assumed | .006 | 1 | .006 | .922 | .343 | .025 | .922 | .155 |
|  | Greenhouse-Geisser | .006 | 1.000 | .006 | .922 | .343 | .025 | .922 | .155 |
|  | Huynh-Feldt | .006 | 1.000 | .006 | .922 | .343 | .025 | .922 | .155 |
|  | Lower-bound | .006 | 1.000 | .006 | .922 | .343 | .025 | .922 | .155 |
| Error(conflict) | Sphericity Assumed | .242 | 36 | .007 |  |  |  |  |  |
|  | Greenhouse-Geisser | .242 | 36.000 | .007 |  |  |  |  |  |
|  | Huynh-Feldt | .242 | 36.000 | .007 |  |  |  |  |  |
|  | Lower-bound | .242 | 36.000 | .007 |  |  |  |  |  |
| emotion * conflict | Sphericity Assumed | .007 | 1 | .007 | 5.507 | .025 | .133 | 5.507 | .627 |
|  | Greenhouse-Geisser | .007 | 1.000 | .007 | 5.507 | .025 | .133 | 5.507 | .627 |
|  | Huynh-Feldt | .007 | 1.000 | .007 | 5.507 | .025 | .133 | 5.507 | .627 |
|  | Lower-bound | .007 | 1.000 | .007 | 5.507 | .025 | .133 | 5.507 | .627 |
| emotion * conflict * power | Sphericity Assumed | .001 | 1 | .001 | .960 | .334 | .026 | .960 | .159 |
|  | Greenhouse-Geisser | .001 | 1.000 | .001 | .960 | .334 | .026 | .960 | .159 |
|  | Huynh-Feldt | .001 | 1.000 | .001 | .960 | .334 | .026 | .960 | .159 |
|  | Lower-bound | .001 | 1.000 | .001 | .960 | .334 | .026 | .960 | .159 |
| Error(emotion*conflict) | Sphericity Assumed | .046 | 36 | .001 |  |  |  |  |  |
|  | Greenhouse-Geisser | .046 | 36.000 | .001 |  |  |  |  |  |
|  | Huynh-Feldt | .046 | 36.000 | .001 |  |  |  |  |  |
|  | Lower-bound | .046 | 36.000 | .001 |  |  |  |  |  |
| a. Computed using alpha = .05 | | | | | | | | | |

| **Tests of Within-Subjects Contrasts** | | | | | | | | | | |
| --- | --- | --- | --- | --- | --- | --- | --- | --- | --- | --- |
| Measure: MEASURE_1 | | | | | | | | | | |
| Source | emotion | conflict | Type III Sum of Squares | df | Mean Square | F | Sig. | Partial Eta Squared | Noncent. Parameter | Observed Power^a^ |
| emotion | Linear |  | .047 | 1 | .047 | 9.222 | .004 | .204 | 9.222 | .840 |
| emotion * power | Linear |  | .001 | 1 | .001 | .124 | .726 | .003 | .124 | .064 |
| Error(emotion) | Linear |  | .183 | 36 | .005 |  |  |  |  |  |
| conflict |  | Linear | .314 | 1 | .314 | 46.807 | .000 | .565 | 46.807 | 1.000 |
| conflict * power |  | Linear | .006 | 1 | .006 | .922 | .343 | .025 | .922 | .155 |
| Error(conflict) |  | Linear | .242 | 36 | .007 |  |  |  |  |  |
| emotion * conflict | Linear | Linear | .007 | 1 | .007 | 5.507 | .025 | .133 | 5.507 | .627 |
| emotion * conflict * power | Linear | Linear | .001 | 1 | .001 | .960 | .334 | .026 | .960 | .159 |
| Error(emotion*conflict) | Linear | Linear | .046 | 36 | .001 |  |  |  |  |  |
| a. Computed using alpha = .05 | | | | | | | | | | |

| **Levene's Test of Equality of Error Variances^a^** | | | | |
| --- | --- | --- | --- | --- |
|  | F | df1 | df2 | Sig. |
| Fear congruent | .720 | 1 | 36 | .402 |
| Fear incongruent | .334 | 1 | 36 | .567 |
| Happy congruent | 2.421 | 1 | 36 | .128 |
| Happy incongruent | .057 | 1 | 36 | .813 |
| Tests the null hypothesis that the error variance of the dependent variable is equal across groups. | | | | |
| a. Design: + power  Within Subjects Design: emotion + conflict + emotion * conflict | | | | |

| **Tests of Between-Subjects Effects** | | | | | | | | |
| --- | --- | --- | --- | --- | --- | --- | --- | --- |
| Measure: MEASURE_1 | | | | | | | | |
| Transformed Variable: Average | | | | | | | | |
| Source | Type III Sum of Squares | df | Mean Square | F | Sig. | Partial Eta Squared | Noncent. Parameter | Observed Power^a^ |
|  | 114.163 | 1 | 114.163 | 2814.164 | .000 | .987 | 2814.164 | 1.000 |
| power | .000 | 1 | .000 | .005 | .946 | .000 | .005 | .051 |
| Error | 1.460 | 36 | .041 |  |  |  |  |  |
| a. Computed using alpha = .05 | | | | | | | | |

**Estimated Marginal Means**

**1. power**

| **Estimates** | | | | |
| --- | --- | --- | --- | --- |
| Measure: MEASURE_1 | | | | |
| power | Mean | Std. Error | 95% Confidence Interval | |
|  |  |  | Lower Bound | Upper Bound |
| 1.00 | .866 | .023 | .819 | .912 |
| 2.00 | .868 | .023 | .821 | .915 |

| **Pairwise Comparisons** | | | | | | |
| --- | --- | --- | --- | --- | --- | --- |
| Measure: MEASURE_1 | | | | | | |
| (I) power | (J) power | Mean Difference (I-J) | Std. Error | Sig.^a^ | 95% Confidence Interval for Difference^a^ | |
|  |  |  |  |  | Lower Bound | Upper Bound |
| 1.00 | 2.00 | -.002 | .033 | .946 | -.069 | .064 |
| 2.00 | 1.00 | .002 | .033 | .946 | -.064 | .069 |
| Based on estimated marginal means | | | | | | |
| a. Adjustment for multiple comparisons: Least Significant Difference (equivalent to no adjustments). | | | | | | |

| **Univariate Tests** | | | | | | | | |
| --- | --- | --- | --- | --- | --- | --- | --- | --- |
| Measure: MEASURE_1 | | | | | | | | |
|  | Sum of Squares | df | Mean Square | F | Sig. | Partial Eta Squared | Noncent. Parameter | Observed Power^a^ |
| Contrast | 4.753E-005 | 1 | 4.753E-005 | .005 | .946 | .000 | .005 | .051 |
| Error | .365 | 36 | .010 |  |  |  |  |  |
| The F tests the effect of power. This test is based on the linearly independent pairwise comparisons among the estimated marginal means. | | | | | | | | |
| a. Computed using alpha = .05 | | | | | | | | |

**2. emotion**

| **Estimates** | | | | |
| --- | --- | --- | --- | --- |
| Measure: MEASURE_1 | | | | |
| emotion | Mean | Std. Error | 95% Confidence Interval | |
|  |  |  | Lower Bound | Upper Bound |
| 1 | .884 | .014 | .856 | .913 |
| 2 | .849 | .020 | .808 | .890 |

| **Pairwise Comparisons** | | | | | | |
| --- | --- | --- | --- | --- | --- | --- |
| Measure: MEASURE_1 | | | | | | |
| (I) emotion | (J) emotion | Mean Difference (I-J) | Std. Error | Sig.^b^ | 95% Confidence Interval for Difference^b^ | |
|  |  |  |  |  | Lower Bound | Upper Bound |
| 1 | 2 | .035^*^ | .012 | .004 | .012 | .059 |
| 2 | 1 | -.035^*^ | .012 | .004 | -.059 | -.012 |
| Based on estimated marginal means | | | | | | |
| *. The mean difference is significant at the .05 level. | | | | | | |
| b. Adjustment for multiple comparisons: Least Significant Difference (equivalent to no adjustments). | | | | | | |

| **Multivariate Tests** | | | | | | | | |
| --- | --- | --- | --- | --- | --- | --- | --- | --- |
|  | Value | F | Hypothesis df | Error df | Sig. | Partial Eta Squared | Noncent. Parameter | Observed Power^b^ |
| Pillai's trace | .204 | 9.222^a^ | 1.000 | 36.000 | .004 | .204 | 9.222 | .840 |
| Wilks' lambda | .796 | 9.222^a^ | 1.000 | 36.000 | .004 | .204 | 9.222 | .840 |
| Hotelling's trace | .256 | 9.222^a^ | 1.000 | 36.000 | .004 | .204 | 9.222 | .840 |
| Roy's largest root | .256 | 9.222^a^ | 1.000 | 36.000 | .004 | .204 | 9.222 | .840 |
| Each F tests the multivariate effect of emotion. These tests are based on the linearly independent pairwise comparisons among the estimated marginal means. | | | | | | | | |
| a. Exact statistic | | | | | | | | |
| b. Computed using alpha = .05 | | | | | | | | |

**3. conflict**

| **Estimates** | | | | |
| --- | --- | --- | --- | --- |
| Measure: MEASURE_1 | | | | |
| conflict | Mean | Std. Error | 95% Confidence Interval | |
|  |  |  | Lower Bound | Upper Bound |
| 1 | .912 | .013 | .886 | .938 |
| 2 | .821 | .021 | .778 | .864 |

| **Pairwise Comparisons** | | | | | | |
| --- | --- | --- | --- | --- | --- | --- |
| Measure: MEASURE_1 | | | | | | |
| (I) conflict | (J) conflict | Mean Difference (I-J) | Std. Error | Sig.^b^ | 95% Confidence Interval for Difference^b^ | |
|  |  |  |  |  | Lower Bound | Upper Bound |
| 1 | 2 | .091^*^ | .013 | .000 | .064 | .118 |
| 2 | 1 | -.091^*^ | .013 | .000 | -.118 | -.064 |
| Based on estimated marginal means | | | | | | |
| *. The mean difference is significant at the .05 level. | | | | | | |
| b. Adjustment for multiple comparisons: Least Significant Difference (equivalent to no adjustments). | | | | | | |

| **Multivariate Tests** | | | | | | | | |
| --- | --- | --- | --- | --- | --- | --- | --- | --- |
|  | Value | F | Hypothesis df | Error df | Sig. | Partial Eta Squared | Noncent. Parameter | Observed Power^b^ |
| Pillai's trace | .565 | 46.807^a^ | 1.000 | 36.000 | .000 | .565 | 46.807 | 1.000 |
| Wilks' lambda | .435 | 46.807^a^ | 1.000 | 36.000 | .000 | .565 | 46.807 | 1.000 |
| Hotelling's trace | 1.300 | 46.807^a^ | 1.000 | 36.000 | .000 | .565 | 46.807 | 1.000 |
| Roy's largest root | 1.300 | 46.807^a^ | 1.000 | 36.000 | .000 | .565 | 46.807 | 1.000 |
| Each F tests the multivariate effect of conflict. These tests are based on the linearly independent pairwise comparisons among the estimated marginal means. | | | | | | | | |
| a. Exact statistic | | | | | | | | |
| b. Computed using alpha = .05 | | | | | | | | |

| **4. power * emotion** | | | | | |
| --- | --- | --- | --- | --- | --- |
| Measure: MEASURE_1 | | | | | |
| power | emotion | Mean | Std. Error | 95% Confidence Interval | |
|  |  |  |  | Lower Bound | Upper Bound |
| 1.00 | 1 | .881 | .020 | .841 | .921 |
|  | 2 | .850 | .028 | .792 | .908 |
| 2.00 | 1 | .887 | .020 | .847 | .928 |
|  | 2 | .848 | .028 | .791 | .906 |

| **5. power * conflict** | | | | | |
| --- | --- | --- | --- | --- | --- |
| Measure: MEASURE_1 | | | | | |
| power | conflict | Mean | Std. Error | 95% Confidence Interval | |
|  |  |  |  | Lower Bound | Upper Bound |
| 1.00 | 1 | .917 | .018 | .880 | .954 |
|  | 2 | .814 | .030 | .752 | .875 |
| 2.00 | 1 | .907 | .018 | .870 | .944 |
|  | 2 | .829 | .030 | .767 | .890 |

| **6. emotion * conflict** | | | | | |
| --- | --- | --- | --- | --- | --- |
| Measure: MEASURE_1 | | | | | |
| emotion | conflict | Mean | Std. Error | 95% Confidence Interval | |
|  |  |  |  | Lower Bound | Upper Bound |
| 1 | 1 | .923 | .013 | .897 | .949 |
|  | 2 | .846 | .018 | .808 | .883 |
| 2 | 1 | .901 | .016 | .870 | .933 |
|  | 2 | .797 | .026 | .744 | .849 |

| **7. power * emotion * conflict** | | | | | | |
| --- | --- | --- | --- | --- | --- | --- |
| Measure: MEASURE_1 | | | | | | |
| power | emotion | conflict | Mean | Std. Error | 95% Confidence Interval | |
|  |  |  |  |  | Lower Bound | Upper Bound |
| 1.00 | 1 | 1 | .929 | .018 | .892 | .966 |
|  |  | 2 | .833 | .026 | .781 | .886 |
|  | 2 | 1 | .906 | .022 | .861 | .950 |
|  |  | 2 | .794 | .037 | .720 | .868 |
| 2.00 | 1 | 1 | .917 | .018 | .880 | .954 |
|  |  | 2 | .858 | .026 | .805 | .910 |
|  | 2 | 1 | .897 | .022 | .852 | .941 |
|  |  | 2 | .799 | .037 | .725 | .874 |

**All the faces in P1**

GLM P7 congruent P7incongruent P8 congruent P8incongruent BY power

/WSFACTOR=electrode 2 Polynomial conflict 2 Polynomial

/METHOD=SSTYPE(3)

/EMMEANS=TABLES(power) COMPARE ADJ(LSD)

/EMMEANS=TABLES(electrode) COMPARE ADJ(LSD)

/EMMEANS=TABLES(conflict) COMPARE ADJ(LSD)

/EMMEANS=TABLES(power*electrode)

/EMMEANS=TABLES(power*conflict)

/EMMEANS=TABLES(electrode*conflict)

/EMMEANS=TABLES(power*electrode*conflict)

/PRINT=DESCRIPTIVE ETASQ OPOWER HOMOGENEITY

/CRITERIA=ALPHA(.05)

/WSDESIGN=electrode conflict electrode*conflict

/DESIGN=power.

**General Linear Model**

| **Notes** | | |
| --- | --- | --- |
| Output Created | | 26-DEC-2020 10:23:20 |
| Comments | |  |
| Input | Data | C:\Users\lenovo\Desktop\p1 120-170.sav |
|  | Active Dataset | Dataset |
|  | Filter | <none> |
|  | Weight | <none> |
|  | Split File | <none> |
|  | N of Rows in Working Data File | 41 |
| Missing Value Handling | Definition of Missing | User-defined missing values are treated as missing. |
|  | Cases Used | Statistics are based on all cases with valid data for all variables in the model. |
| Syntax | | GLM P7 congruent P7incongruent P8 congruent P8incongruent BY power  /WSFACTOR=electrode 2 Polynomial conflict 2 Polynomial  /METHOD=SSTYPE(3)  /EMMEANS=TABLES(power) COMPARE ADJ(LSD)  /EMMEANS=TABLES(electrode) COMPARE ADJ(LSD)  /EMMEANS=TABLES(conflict) COMPARE ADJ(LSD)  /EMMEANS=TABLES(power*electrode)  /EMMEANS=TABLES(power*conflict)  /EMMEANS=TABLES(electrode*conflict  /EMMEANS=TABLES(power*electrode*conflict)  /PRINT=DESCRIPTIVE ETASQ OPOWER HOMOGENEITY  /CRITERIA=ALPHA(.05)  /WSDESIGN=electrode conflict electrode*conflict  /DESIGN=power. |
| Resources | Processor Time | 00:00:00.05 |
|  | Elapsed Time | 00:00:00.06 |

| **Within-Subjects Factors** | | |
| --- | --- | --- |
| Measure: MEASURE_1 | | |
| electrode | conflict | Dependent Variable |
| 1 | 1 | P7congruent |
|  | 2 | P7incongruent |
| 2 | 1 | P8 congruent |
|  | 2 | P8incongruent |

| **Between-Subjects Factors** | | |
| --- | --- | --- |
|  | | N |
| power | 1.00 | 19 |
|  | 2.00 | 19 |

| **Descriptive Statistics** | | | | |
| --- | --- | --- | --- | --- |
|  | power | Mean | Std. Deviation | N |
| P7 congruent | 1.00 | -1.5278 | 3.33437 | 19 |
|  | 2.00 | -.1053 | 2.12191 | 19 |
|  | Total | -.8165 | 2.84933 | 38 |
| P7incongruent | 1.00 | -1.4174 | 3.14275 | 19 |
|  | 2.00 | -.0599 | 2.26729 | 19 |
|  | Total | -.7386 | 2.78908 | 38 |
| P8 congruent | 1.00 | -1.6953 | 4.27562 | 19 |
|  | 2.00 | .4800 | 3.53475 | 19 |
|  | Total | -.6077 | 4.02327 | 38 |
| P8incongruent | 1.00 | -1.5339 | 4.15954 | 19 |
|  | 2.00 | .2337 | 3.81371 | 19 |
|  | Total | -.6501 | 4.03670 | 38 |

| **Box's Test of Equality of Covariance Matrices^a^** | |
| --- | --- |
| Box's M | 23.476 |
| F | 2.063 |
| df1 | 10 |
| df2 | 6196.016 |
| Sig. | .024 |
| Tests the null hypothesis that the observed covariance matrices of the dependent variables are equal across groups. | |
| a. Design: + power  Within Subjects Design: electrode + conflict + electrode * conflict | |

| **Multivariate Tests^a^** | | | | | | | | | |
| --- | --- | --- | --- | --- | --- | --- | --- | --- | --- |
| Effect | | Value | F | Hypothesis df | Error df | Sig. | Partial Eta Squared | Noncent. Parameter | Observed Power^c^ |
| electrode | Pillai's Trace | .002 | .087^b^ | 1.000 | 36.000 | .769 | .002 | .087 | .060 |
|  | Wilks' Lambda | .998 | .087^b^ | 1.000 | 36.000 | .769 | .002 | .087 | .060 |
|  | Hotelling's Trace | .002 | .087^b^ | 1.000 | 36.000 | .769 | .002 | .087 | .060 |
|  | Roy's Largest Root | .002 | .087^b^ | 1.000 | 36.000 | .769 | .002 | .087 | .060 |
| electrode * power | Pillai's Trace | .009 | .333^b^ | 1.000 | 36.000 | .567 | .009 | .333 | .087 |
|  | Wilks' Lambda | .991 | .333^b^ | 1.000 | 36.000 | .567 | .009 | .333 | .087 |
|  | Hotelling's Trace | .009 | .333^b^ | 1.000 | 36.000 | .567 | .009 | .333 | .087 |
|  | Roy's Largest Root | .009 | .333^b^ | 1.000 | 36.000 | .567 | .009 | .333 | .087 |
| conflict | Pillai's Trace | .001 | .042^b^ | 1.000 | 36.000 | .838 | .001 | .042 | .055 |
|  | Wilks' Lambda | .999 | .042^b^ | 1.000 | 36.000 | .838 | .001 | .042 | .055 |
|  | Hotelling's Trace | .001 | .042^b^ | 1.000 | 36.000 | .838 | .001 | .042 | .055 |
|  | Roy's Largest Root | .001 | .042^b^ | 1.000 | 36.000 | .838 | .001 | .042 | .055 |
| conflict * power | Pillai's Trace | .050 | 1.884^b^ | 1.000 | 36.000 | .178 | .050 | 1.884 | .267 |
|  | Wilks' Lambda | .950 | 1.884^b^ | 1.000 | 36.000 | .178 | .050 | 1.884 | .267 |
|  | Hotelling's Trace | .052 | 1.884^b^ | 1.000 | 36.000 | .178 | .050 | 1.884 | .267 |
|  | Roy's Largest Root | .052 | 1.884^b^ | 1.000 | 36.000 | .178 | .050 | 1.884 | .267 |
| electrode * conflict | Pillai's Trace | .023 | .850^b^ | 1.000 | 36.000 | .363 | .023 | .850 | .146 |
|  | Wilks' Lambda | .977 | .850^b^ | 1.000 | 36.000 | .363 | .023 | .850 | .146 |
|  | Hotelling's Trace | .024 | .850^b^ | 1.000 | 36.000 | .363 | .023 | .850 | .146 |
|  | Roy's Largest Root | .024 | .850^b^ | 1.000 | 36.000 | .363 | .023 | .850 | .146 |
| electrode * conflict * power | Pillai's Trace | .046 | 1.724^b^ | 1.000 | 36.000 | .198 | .046 | 1.724 | .248 |
|  | Wilks' Lambda | .954 | 1.724^b^ | 1.000 | 36.000 | .198 | .046 | 1.724 | .248 |
|  | Hotelling's Trace | .048 | 1.724^b^ | 1.000 | 36.000 | .198 | .046 | 1.724 | .248 |
|  | Roy's Largest Root | .048 | 1.724^b^ | 1.000 | 36.000 | .198 | .046 | 1.724 | .248 |
| a. Design: + power  Within Subjects Design: electrode + conflict + electrode * conflict | | | | | | | | | |
| b. Exact statistic | | | | | | | | | |
| c. Computed using alpha = .05 | | | | | | | | | |

| **Mauchly's Test of Sphericity^a^** | | | | | | | |
| --- | --- | --- | --- | --- | --- | --- | --- |
| Measure: MEASURE_1 | | | | | | | |
| Within Subjects Effect | Mauchly's W | Approx. Chi-Square | df | Sig. | Epsilon^b^ | | |
|  |  |  |  |  | Greenhouse-Geisser | Huynh-Feldt | Lower-bound |
| electrode | 1.000 | .000 | 0 | . | 1.000 | 1.000 | 1.000 |
| conflict | 1.000 | .000 | 0 | . | 1.000 | 1.000 | 1.000 |
| electrode * conflict | 1.000 | .000 | 0 | . | 1.000 | 1.000 | 1.000 |
| Tests the null hypothesis that the error covariance matrix of the orthonormalized transformed dependent variables is proportional to an identity matrix. | | | | | | | |
| a. Design: + power  Within Subjects Design: electrode + conflict + electrode * conflict | | | | | | | |
| b. May be used to adjust the degrees of freedom for the averaged tests of significance. Corrected tests are displayed in the Tests of Within-Subjects Effects table. | | | | | | | |

| **Tests of Within-Subjects Effects** | | | | | | | | | |
| --- | --- | --- | --- | --- | --- | --- | --- | --- | --- |
| Measure: MEASURE_1 | | | | | | | | | |
| Source | | Type III Sum of Squares | df | Mean Square | F | Sig. | Partial Eta Squared | Noncent. Parameter | Observed Power^a^ |
| electrode | Sphericity Assumed | .840 | 1 | .840 | .087 | .769 | .002 | .087 | .060 |
|  | Greenhouse-Geisser | .840 | 1.000 | .840 | .087 | .769 | .002 | .087 | .060 |
|  | Huynh-Feldt | .840 | 1.000 | .840 | .087 | .769 | .002 | .087 | .060 |
|  | Lower-bound | .840 | 1.000 | .840 | .087 | .769 | .002 | .087 | .060 |
| electrode * power | Sphericity Assumed | 3.212 | 1 | 3.212 | .333 | .567 | .009 | .333 | .087 |
|  | Greenhouse-Geisser | 3.212 | 1.000 | 3.212 | .333 | .567 | .009 | .333 | .087 |
|  | Huynh-Feldt | 3.212 | 1.000 | 3.212 | .333 | .567 | .009 | .333 | .087 |
|  | Lower-bound | 3.212 | 1.000 | 3.212 | .333 | .567 | .009 | .333 | .087 |
| Error(electrode) | Sphericity Assumed | 346.904 | 36 | 9.636 |  |  |  |  |  |
|  | Greenhouse-Geisser | 346.904 | 36.000 | 9.636 |  |  |  |  |  |
|  | Huynh-Feldt | 346.904 | 36.000 | 9.636 |  |  |  |  |  |
|  | Lower-bound | 346.904 | 36.000 | 9.636 |  |  |  |  |  |
| conflict | Sphericity Assumed | .012 | 1 | .012 | .042 | .838 | .001 | .042 | .055 |
|  | Greenhouse-Geisser | .012 | 1.000 | .012 | .042 | .838 | .001 | .042 | .055 |
|  | Huynh-Feldt | .012 | 1.000 | .012 | .042 | .838 | .001 | .042 | .055 |
|  | Lower-bound | .012 | 1.000 | .012 | .042 | .838 | .001 | .042 | .055 |
| conflict * power | Sphericity Assumed | .531 | 1 | .531 | 1.884 | .178 | .050 | 1.884 | .267 |
|  | Greenhouse-Geisser | .531 | 1.000 | .531 | 1.884 | .178 | .050 | 1.884 | .267 |
|  | Huynh-Feldt | .531 | 1.000 | .531 | 1.884 | .178 | .050 | 1.884 | .267 |
|  | Lower-bound | .531 | 1.000 | .531 | 1.884 | .178 | .050 | 1.884 | .267 |
| Error(conflict) | Sphericity Assumed | 10.140 | 36 | .282 |  |  |  |  |  |
|  | Greenhouse-Geisser | 10.140 | 36.000 | .282 |  |  |  |  |  |
|  | Huynh-Feldt | 10.140 | 36.000 | .282 |  |  |  |  |  |
|  | Lower-bound | 10.140 | 36.000 | .282 |  |  |  |  |  |
| electrode * conflict | Sphericity Assumed | .138 | 1 | .138 | .850 | .363 | .023 | .850 | .146 |
|  | Greenhouse-Geisser | .138 | 1.000 | .138 | .850 | .363 | .023 | .850 | .146 |
|  | Huynh-Feldt | .138 | 1.000 | .138 | .850 | .363 | .023 | .850 | .146 |
|  | Lower-bound | .138 | 1.000 | .138 | .850 | .363 | .023 | .850 | .146 |
| electrode * conflict * power | Sphericity Assumed | .279 | 1 | .279 | 1.724 | .198 | .046 | 1.724 | .248 |
|  | Greenhouse-Geisser | .279 | 1.000 | .279 | 1.724 | .198 | .046 | 1.724 | .248 |
|  | Huynh-Feldt | .279 | 1.000 | .279 | 1.724 | .198 | .046 | 1.724 | .248 |
|  | Lower-bound | .279 | 1.000 | .279 | 1.724 | .198 | .046 | 1.724 | .248 |
| Error(electrode*conflict) | Sphericity Assumed | 5.827 | 36 | .162 |  |  |  |  |  |
|  | Greenhouse-Geisser | 5.827 | 36.000 | .162 |  |  |  |  |  |
|  | Huynh-Feldt | 5.827 | 36.000 | .162 |  |  |  |  |  |
|  | Lower-bound | 5.827 | 36.000 | .162 |  |  |  |  |  |
| a. Computed using alpha = .05 | | | | | | | | | |

| **Tests of Within-Subjects Contrasts** | | | | | | | | | | |
| --- | --- | --- | --- | --- | --- | --- | --- | --- | --- | --- |
| Measure: MEASURE_1 | | | | | | | | | | |
| Source | electrode | conflict | Type III Sum of Squares | df | Mean Square | F | Sig. | Partial Eta Squared | Noncent. Parameter | Observed Power^a^ |
| electrode | Linear |  | .840 | 1 | .840 | .087 | .769 | .002 | .087 | .060 |
| electrode * power | Linear |  | 3.212 | 1 | 3.212 | .333 | .567 | .009 | .333 | .087 |
| Error(electrode) | Linear |  | 346.904 | 36 | 9.636 |  |  |  |  |  |
| conflict |  | Linear | .012 | 1 | .012 | .042 | .838 | .001 | .042 | .055 |
| conflict * power |  | Linear | .531 | 1 | .531 | 1.884 | .178 | .050 | 1.884 | .267 |
| Error(conflict) |  | Linear | 10.140 | 36 | .282 |  |  |  |  |  |
| electrode * conflict | Linear | Linear | .138 | 1 | .138 | .850 | .363 | .023 | .850 | .146 |
| electrode * conflict * power | Linear | Linear | .279 | 1 | .279 | 1.724 | .198 | .046 | 1.724 | .248 |
| Error(electrode* conflict) | Linear | Linear | 5.827 | 36 | .162 |  |  |  |  |  |
| a. Computed using alpha = .05 | | | | | | | | | | |

| **Levene's Test of Equality of Error Variances^a^** | | | | |
| --- | --- | --- | --- | --- |
|  | F | df1 | df2 | Sig. |
| P7congruent | 5.768 | 1 | 36 | .022 |
| P7incongruent | 2.587 | 1 | 36 | .116 |
| P8congruent | .690 | 1 | 36 | .412 |
| P8incongruent | .078 | 1 | 36 | .782 |
| Tests the null hypothesis that the error variance of the dependent variable is equal across groups. | | | | |
| a. Design: + power  Within Subjects Design: electrode + conflict + electrode * conflict | | | | |

| **Tests of Between-Subjects Effects** | | | | | | | | |
| --- | --- | --- | --- | --- | --- | --- | --- | --- |
| Measure: MEASURE_1 | | | | | | | | |
| Transformed Variable: Average | | | | | | | | |
| Source | Type III Sum of Squares | df | Mean Square | F | Sig. | Partial Eta Squared | Noncent. Parameter | Observed Power^a^ |
|  | 75.170 | 1 | 75.170 | 2.057 | .160 | .054 | 2.057 | .287 |
| power | 107.339 | 1 | 107.339 | 2.937 | .095 | .075 | 2.937 | .385 |
| Error | 1315.801 | 36 | 36.550 |  |  |  |  |  |
| a. Computed using alpha = .05 | | | | | | | | |

**Estimated Marginal Means**

**1. power**

| **Estimates** | | | | |
| --- | --- | --- | --- | --- |
| Measure: MEASURE_1 | | | | |
| power | Mean | Std. Error | 95% Confidence Interval | |
|  |  |  | Lower Bound | Upper Bound |
| 1.00 | -1.544 | .693 | -2.950 | -.137 |
| 2.00 | .137 | .693 | -1.269 | 1.544 |

| **Pairwise Comparisons** | | | | | | |
| --- | --- | --- | --- | --- | --- | --- |
| Measure: MEASURE_1 | | | | | | |
| (I) power | (J) power | Mean Difference (I-J) | Std. Error | Sig.^a^ | 95% Confidence Interval for Difference^a^ | |
|  |  |  |  |  | Lower Bound | Upper Bound |
| 1.00 | 2.00 | -1.681 | .981 | .095 | -3.670 | .308 |
| 2.00 | 1.00 | 1.681 | .981 | .095 | -.308 | 3.670 |
| Based on estimated marginal means | | | | | | |
| a. Adjustment for multiple comparisons: Least Significant Difference (equivalent to no adjustments). | | | | | | |

| **Univariate Tests** | | | | | | | | |
| --- | --- | --- | --- | --- | --- | --- | --- | --- |
| Measure: MEASURE_1 | | | | | | | | |
|  | Sum of Squares | df | Mean Square | F | Sig. | Partial Eta Squared | Noncent. Parameter | Observed Power^a^ |
| Contrast | 26.835 | 1 | 26.835 | 2.937 | .095 | .075 | 2.937 | .385 |
| Error | 328.950 | 36 | 9.138 |  |  |  |  |  |
| The F tests the effect of power. This test is based on the linearly independent pairwise comparisons among the estimated marginal means. | | | | | | | | |
| a. Computed using alpha = .05 | | | | | | | | |

**2. electrode**

| **Estimates** | | | | |
| --- | --- | --- | --- | --- |
| Measure: MEASURE_1 | | | | |
| electrode | Mean | Std. Error | 95% Confidence Interval | |
|  |  |  | Lower Bound | Upper Bound |
| 1 | -.778 | .446 | -1.683 | .127 |
| 2 | -.629 | .639 | -1.925 | .668 |

| **Pairwise Comparisons** | | | | | | |
| --- | --- | --- | --- | --- | --- | --- |
| Measure: MEASURE_1 | | | | | | |
| (I) electrode | (J) electrode | Mean Difference (I-J) | Std. Error | Sig.^a^ | 95% Confidence Interval for Difference^a^ | |
|  |  |  |  |  | Lower Bound | Upper Bound |
| 1 | 2 | -.149 | .504 | .769 | -1.170 | .873 |
| 2 | 1 | .149 | .504 | .769 | -.873 | 1.170 |
| Based on estimated marginal means | | | | | | |
| a. Adjustment for multiple comparisons: Least Significant Difference (equivalent to no adjustments). | | | | | | |

| **Multivariate Tests** | | | | | | | | |
| --- | --- | --- | --- | --- | --- | --- | --- | --- |
|  | Value | F | Hypothesis df | Error df | Sig. | Partial Eta Squared | Noncent. Parameter | Observed Power^b^ |
| Pillai's trace | .002 | .087^a^ | 1.000 | 36.000 | .769 | .002 | .087 | .060 |
| Wilks' lambda | .998 | .087^a^ | 1.000 | 36.000 | .769 | .002 | .087 | .060 |
| Hotelling's trace | .002 | .087^a^ | 1.000 | 36.000 | .769 | .002 | .087 | .060 |
| Roy's largest root | .002 | .087^a^ | 1.000 | 36.000 | .769 | .002 | .087 | .060 |
| Each F tests the multivariate effect of electrode. These tests are based on the linearly independent pairwise comparisons among the estimated marginal means. | | | | | | | | |
| a. Exact statistic | | | | | | | | |
| b. Computed using alpha = .05 | | | | | | | | |

**3. conflict**

| **Estimates** | | | | |
| --- | --- | --- | --- | --- |
| Measure: MEASURE_1 | | | | |
| conflict | Mean | Std. Error | 95% Confidence Interval | |
|  |  |  | Lower Bound | Upper Bound |
| 1 | -.712 | .492 | -1.711 | .286 |
| 2 | -.694 | .492 | -1.693 | .304 |

| **Pairwise Comparisons** | | | | | | |
| --- | --- | --- | --- | --- | --- | --- |
| Measure: MEASURE_1 | | | | | | |
| (I) conflict | (J) conflict | Mean Difference (I-J) | Std. Error | Sig.^a^ | 95% Confidence Interval for Difference^a^ | |
|  |  |  |  |  | Lower Bound | Upper Bound |
| 1 | 2 | -.018 | .086 | .838 | -.192 | .157 |
| 2 | 1 | .018 | .086 | .838 | -.157 | .192 |
| Based on estimated marginal means | | | | | | |
| a. Adjustment for multiple comparisons: Least Significant Difference (equivalent to no adjustments). | | | | | | |

| **Multivariate Tests** | | | | | | | | |
| --- | --- | --- | --- | --- | --- | --- | --- | --- |
|  | Value | F | Hypothesis df | Error df | Sig. | Partial Eta Squared | Noncent. Parameter | Observed Power^b^ |
| Pillai's trace | .001 | .042^a^ | 1.000 | 36.000 | .838 | .001 | .042 | .055 |
| Wilks' lambda | .999 | .042^a^ | 1.000 | 36.000 | .838 | .001 | .042 | .055 |
| Hotelling's trace | .001 | .042^a^ | 1.000 | 36.000 | .838 | .001 | .042 | .055 |
| Roy's largest root | .001 | .042^a^ | 1.000 | 36.000 | .838 | .001 | .042 | .055 |
| Each F tests the multivariate effect of conflict. These tests are based on the linearly independent pairwise comparisons among the estimated marginal means. | | | | | | | | |
| a. Exact statistic | | | | | | | | |
| b. Computed using alpha = .05 | | | | | | | | |

| **4. power * electrode** | | | | | |
| --- | --- | --- | --- | --- | --- |
| Measure: MEASURE_1 | | | | | |
| power | electrode | Mean | Std. Error | 95% Confidence Interval | |
|  |  |  |  | Lower Bound | Upper Bound |
| 1.00 | 1 | -1.473 | .631 | -2.752 | -.193 |
|  | 2 | -1.615 | .904 | -3.448 | .219 |
| 2.00 | 1 | -.083 | .631 | -1.362 | 1.197 |
|  | 2 | .357 | .904 | -1.477 | 2.190 |

| **5. power * conflict** | | | | | |
| --- | --- | --- | --- | --- | --- |
| Measure: MEASURE_1 | | | | | |
| power | conflict | Mean | Std. Error | 95% Confidence Interval | |
|  |  |  |  | Lower Bound | Upper Bound |
| 1.00 | 1 | -1.612 | .696 | -3.024 | -.200 |
|  | 2 | -1.476 | .696 | -2.887 | -.064 |
| 2.00 | 1 | .187 | .696 | -1.225 | 1.599 |
|  | 2 | .087 | .696 | -1.325 | 1.499 |

| **6. electrode *** **conflict** | | | | | |
| --- | --- | --- | --- | --- | --- |
| Measure: MEASURE_1 | | | | | |
| electrode | conflict | Mean | Std. Error | 95% Confidence Interval | |
|  |  |  |  | Lower Bound | Upper Bound |
| 1 | 1 | -.817 | .453 | -1.736 | .103 |
|  | 2 | -.739 | .445 | -1.640 | .163 |
| 2 | 1 | -.608 | .636 | -1.898 | .683 |
|  | 2 | -.650 | .647 | -1.963 | .663 |

| **7. power * electrode * conflict** | | | | | | |
| --- | --- | --- | --- | --- | --- | --- |
| Measure: MEASURE_1 | | | | | | |
| power | electrode | conflict | Mean | Std. Error | 95% Confidence Interval | |
|  |  |  |  |  | Lower Bound | Upper Bound |
| 1.00 | 1 | 1 | -1.528 | .641 | -2.828 | -.227 |
|  |  | 2 | -1.417 | .629 | -2.692 | -.142 |
|  | 2 | 1 | -1.695 | .900 | -3.520 | .130 |
|  |  | 2 | -1.534 | .915 | -3.391 | .323 |
| 2.00 | 1 | 1 | -.105 | .641 | -1.406 | 1.195 |
|  |  | 2 | -.060 | .629 | -1.335 | 1.215 |
|  | 2 | 1 | .480 | .900 | -1.345 | 2.305 |
|  |  | 2 | .234 | .915 | -1.623 | 2.090 |

**All the faces in N170**

GLM P7 congruent P7incongruent P8 congruent P8incongruent BY power

/WSFACTOR=electrode 2 Polynomial conflict 2 Polynomial

/METHOD=SSTYPE(3)

/EMMEANS=TABLES(power) COMPARE ADJ(LSD)

/EMMEANS=TABLES(electrode) COMPARE ADJ(LSD)

/EMMEANS=TABLES(conflict) COMPARE ADJ(LSD)

/EMMEANS=TABLES(power*electrode)

/EMMEANS=TABLES(power* conflict)

/EMMEANS=TABLES(electrode* conflict)

/EMMEANS=TABLES(power*electrode* conflict)

/PRINT=DESCRIPTIVE ETASQ OPOWER HOMOGENEITY

/CRITERIA=ALPHA(.05)

/WSDESIGN=electrode conflict electrode* conflict

/DESIGN=power.

**General Linear Model**

| **Notes** | | |
| --- | --- | --- |
| Output Created | | 26-DEC-2020 10:21:24 |
| Comments | |  |
| Input | Data | C:\Users\lenovo\Desktop \ERP data\N170 150-180.sav |
|  | Active Dataset | Dataset |
|  | Filter | <none> |
|  | Weight | <none> |
|  | Split File | <none> |
|  | N of Rows in Working Data File | 41 |
| Missing Value Handling | Definition of Missing | User-defined missing values are treated as missing. |
|  | Cases Used | Statistics are based on all cases with valid data for all variables in the model. |
| Syntax | | GLM P7 congruent P7incongruent P8 congruent P8incongruent BY power  /WSFACTOR=electrode 2 Polynomial conflict 2 Polynomial  /METHOD=SSTYPE(3)  /EMMEANS=TABLES(power) COMPARE ADJ(LSD)  /EMMEANS=TABLES(electrode) COMPARE ADJ(LSD)  /EMMEANS=TABLES(conflict) COMPARE ADJ(LSD)  /EMMEANS=TABLES(power*electrode)  /EMMEANS=TABLES(power* conflict)  /EMMEANS=TABLES(electrode* conflict)  /EMMEANS=TABLES(power*electrode* conflict)  /PRINT=DESCRIPTIVE ETASQ OPOWER HOMOGENEITY  /CRITERIA=ALPHA(.05)  /WSDESIGN=electrode conflict electrode* conflict  /DESIGN=power. |
| Resources | Processor Time | 00:00:00.06 |
|  | Elapsed Time | 00:00:00.06 |

| **Within-Subjects Factors** | | |
| --- | --- | --- |
| Measure: MEASURE_1 | | |
| electrode | conflict | Dependent Variable |
| 1 | 1 | P7 congruent |
|  | 2 | P7incongruent |
| 2 | 1 | P8 congruent |
|  | 2 | P8incongruent |

| **Between-Subjects Factors** | | |
| --- | --- | --- |
|  | | N |
| power | 1.00 | 19 |
|  | 2.00 | 19 |

| **Descriptive Statistics** | | | | |
| --- | --- | --- | --- | --- |
|  | power | Mean | Std. Deviation | N |
| P7 congruent | 1.00 | -4.7921 | 4.84918 | 19 |
|  | 2.00 | -2.4988 | 2.50176 | 19 |
|  | Total | -3.6455 | 3.97927 | 38 |
| P7 incongruent | 1.00 | -4.7606 | 4.81401 | 19 |
|  | 2.00 | -2.4700 | 2.57960 | 19 |
|  | Total | -3.6153 | 3.98228 | 38 |
| P8 congruent | 1.00 | -6.2484 | 6.53531 | 19 |
|  | 2.00 | -3.3256 | 4.13407 | 19 |
|  | Total | -4.7870 | 5.59336 | 38 |
| P8 incongruent | 1.00 | -6.0620 | 6.47332 | 19 |
|  | 2.00 | -3.3925 | 4.46223 | 19 |
|  | Total | -4.7272 | 5.64820 | 38 |

| **Box's Test of Equality of Covariance Matrices^a^** | |
| --- | --- |
| Box's M | 26.200 |
| F | 2.303 |
| df1 | 10 |
| df2 | 6196.016 |
| Sig. | .011 |
| Tests the null hypothesis that the observed covariance matrices of the dependent variables are equal across groups. | |
| a. Design: + power  Within Subjects Design: electrode + conflict + electrode * conflict | |

| **Multivariate Tests^a^** | | | | | | | | | |
| --- | --- | --- | --- | --- | --- | --- | --- | --- | --- |
| Effect | | Value | F | Hypothesis df | Error df | Sig. | Partial Eta Squared | Noncent. Parameter | Observed Power^c^ |
| electrode | Pillai's Trace | .081 | 3.164^b^ | 1.000 | 36.000 | .084 | .081 | 3.164 | .410 |
|  | Wilks' Lambda | .919 | 3.164^b^ | 1.000 | 36.000 | .084 | .081 | 3.164 | .410 |
|  | Hotelling's Trace | .088 | 3.164^b^ | 1.000 | 36.000 | .084 | .081 | 3.164 | .410 |
|  | Roy's Largest Root | .088 | 3.164^b^ | 1.000 | 36.000 | .084 | .081 | 3.164 | .410 |
| electrode * power | Pillai's Trace | .004 | .158^b^ | 1.000 | 36.000 | .693 | .004 | .158 | .067 |
|  | Wilks' Lambda | .996 | .158^b^ | 1.000 | 36.000 | .693 | .004 | .158 | .067 |
|  | Hotelling's Trace | .004 | .158^b^ | 1.000 | 36.000 | .693 | .004 | .158 | .067 |
|  | Roy's Largest Root | .004 | .158^b^ | 1.000 | 36.000 | .693 | .004 | .158 | .067 |
| conflict | Pillai's Trace | .005 | .191^b^ | 1.000 | 36.000 | .665 | .005 | .191 | .071 |
|  | Wilks' Lambda | .995 | .191^b^ | 1.000 | 36.000 | .665 | .005 | .191 | .071 |
|  | Hotelling's Trace | .005 | .191^b^ | 1.000 | 36.000 | .665 | .005 | .191 | .071 |
|  | Roy's Largest Root | .005 | .191^b^ | 1.000 | 36.000 | .665 | .005 | .191 | .071 |
| conflict * power | Pillai's Trace | .011 | .386^b^ | 1.000 | 36.000 | .538 | .011 | .386 | .093 |
|  | Wilks' Lambda | .989 | .386^b^ | 1.000 | 36.000 | .538 | .011 | .386 | .093 |
|  | Hotelling's Trace | .011 | .386^b^ | 1.000 | 36.000 | .538 | .011 | .386 | .093 |
|  | Roy's Largest Root | .011 | .386^b^ | 1.000 | 36.000 | .538 | .011 | .386 | .093 |
| electrode * conflict | Pillai's Trace | .001 | .036^b^ | 1.000 | 36.000 | .851 | .001 | .036 | .054 |
|  | Wilks' Lambda | .999 | .036^b^ | 1.000 | 36.000 | .851 | .001 | .036 | .054 |
|  | Hotelling's Trace | .001 | .036^b^ | 1.000 | 36.000 | .851 | .001 | .036 | .054 |
|  | Roy's Largest Root | .001 | .036^b^ | 1.000 | 36.000 | .851 | .001 | .036 | .054 |
| electrode * conflict * power | Pillai's Trace | .018 | .643^b^ | 1.000 | 36.000 | .428 | .018 | .643 | .122 |
|  | Wilks' Lambda | .982 | .643^b^ | 1.000 | 36.000 | .428 | .018 | .643 | .122 |
|  | Hotelling's Trace | .018 | .643^b^ | 1.000 | 36.000 | .428 | .018 | .643 | .122 |
|  | Roy's Largest Root | .018 | .643^b^ | 1.000 | 36.000 | .428 | .018 | .643 | .122 |
| a. Design: + power  Within Subjects Design: electrode + conflict + electrode * conflict | | | | | | | | | |
| b. Exact statistic | | | | | | | | | |
| c. Computed using alpha = .05 | | | | | | | | | |

| **Mauchly's Test of Sphericity^a^** | | | | | | | |
| --- | --- | --- | --- | --- | --- | --- | --- |
| Measure: MEASURE_1 | | | | | | | |
| Within Subjects Effect | Mauchly's W | Approx. Chi-Square | df | Sig. | Epsilon^b^ | | |
|  |  |  |  |  | Greenhouse-Geisser | Huynh-Feldt | Lower-bound |
| electrode | 1.000 | .000 | 0 | . | 1.000 | 1.000 | 1.000 |
| conflict | 1.000 | .000 | 0 | . | 1.000 | 1.000 | 1.000 |
| electrode * conflict | 1.000 | .000 | 0 | . | 1.000 | 1.000 | 1.000 |
| Tests the null hypothesis that the error covariance matrix of the orthonormalized transformed dependent variables is proportional to an identity matrix. | | | | | | | |
| a. Design: + power  Within Subjects Design: electrode + conflict + electrode * conflict | | | | | | | |
| b. May be used to adjust the degrees of freedom for the averaged tests of significance. Corrected tests are displayed in the Tests of Within-Subjects Effects table. | | | | | | | |

| **Tests of Within-Subjects Effects** | | | | | | | | | |
| --- | --- | --- | --- | --- | --- | --- | --- | --- | --- |
| Measure: MEASURE_1 | | | | | | | | | |
| Source | | Type III Sum of Squares | df | Mean Square | F | Sig. | Partial Eta Squared | Noncent. Parameter | Observed Power^a^ |
| electrode | Sphericity Assumed | 48.243 | 1 | 48.243 | 3.164 | .084 | .081 | 3.164 | .410 |
|  | Greenhouse-Geisser | 48.243 | 1.000 | 48.243 | 3.164 | .084 | .081 | 3.164 | .410 |
|  | Huynh-Feldt | 48.243 | 1.000 | 48.243 | 3.164 | .084 | .081 | 3.164 | .410 |
|  | Lower-bound | 48.243 | 1.000 | 48.243 | 3.164 | .084 | .081 | 3.164 | .410 |
| electrode * power | Sphericity Assumed | 2.416 | 1 | 2.416 | .158 | .693 | .004 | .158 | .067 |
|  | Greenhouse-Geisser | 2.416 | 1.000 | 2.416 | .158 | .693 | .004 | .158 | .067 |
|  | Huynh-Feldt | 2.416 | 1.000 | 2.416 | .158 | .693 | .004 | .158 | .067 |
|  | Lower-bound | 2.416 | 1.000 | 2.416 | .158 | .693 | .004 | .158 | .067 |
| Error(electrode) | Sphericity Assumed | 548.849 | 36 | 15.246 |  |  |  |  |  |
|  | Greenhouse-Geisser | 548.849 | 36.000 | 15.246 |  |  |  |  |  |
|  | Huynh-Feldt | 548.849 | 36.000 | 15.246 |  |  |  |  |  |
|  | Lower-bound | 548.849 | 36.000 | 15.246 |  |  |  |  |  |
| conflict | Sphericity Assumed | .077 | 1 | .077 | .191 | .665 | .005 | .191 | .071 |
|  | Greenhouse-Geisser | .077 | 1.000 | .077 | .191 | .665 | .005 | .191 | .071 |
|  | Huynh-Feldt | .077 | 1.000 | .077 | .191 | .665 | .005 | .191 | .071 |
|  | Lower-bound | .077 | 1.000 | .077 | .191 | .665 | .005 | .191 | .071 |
| conflict * power | Sphericity Assumed | .156 | 1 | .156 | .386 | .538 | .011 | .386 | .093 |
|  | Greenhouse-Geisser | .156 | 1.000 | .156 | .386 | .538 | .011 | .386 | .093 |
|  | Huynh-Feldt | .156 | 1.000 | .156 | .386 | .538 | .011 | .386 | .093 |
|  | Lower-bound | .156 | 1.000 | .156 | .386 | .538 | .011 | .386 | .093 |
| Error(conflict) | Sphericity Assumed | 14.510 | 36 | .403 |  |  |  |  |  |
|  | Greenhouse-Geisser | 14.510 | 36.000 | .403 |  |  |  |  |  |
|  | Huynh-Feldt | 14.510 | 36.000 | .403 |  |  |  |  |  |
|  | Lower-bound | 14.510 | 36.000 | .403 |  |  |  |  |  |
| electrode * conflict | Sphericity Assumed | .008 | 1 | .008 | .036 | .851 | .001 | .036 | .054 |
|  | Greenhouse-Geisser | .008 | 1.000 | .008 | .036 | .851 | .001 | .036 | .054 |
|  | Huynh-Feldt | .008 | 1.000 | .008 | .036 | .851 | .001 | .036 | .054 |
|  | Lower-bound | .008 | 1.000 | .008 | .036 | .851 | .001 | .036 | .054 |
| electrode * conflict * power | Sphericity Assumed | .149 | 1 | .149 | .643 | .428 | .018 | .643 | .122 |
|  | Greenhouse-Geisser | .149 | 1.000 | .149 | .643 | .428 | .018 | .643 | .122 |
|  | Huynh-Feldt | .149 | 1.000 | .149 | .643 | .428 | .018 | .643 | .122 |
|  | Lower-bound | .149 | 1.000 | .149 | .643 | .428 | .018 | .643 | .122 |
| Error(electrode*conflict) | Sphericity Assumed | 8.345 | 36 | .232 |  |  |  |  |  |
|  | Greenhouse-Geisser | 8.345 | 36.000 | .232 |  |  |  |  |  |
|  | Huynh-Feldt | 8.345 | 36.000 | .232 |  |  |  |  |  |
|  | Lower-bound | 8.345 | 36.000 | .232 |  |  |  |  |  |
| a. Computed using alpha = .05 | | | | | | | | | |

| **Tests of Within-Subjects Contrasts** | | | | | | | | | | |
| --- | --- | --- | --- | --- | --- | --- | --- | --- | --- | --- |
| Measure: MEASURE_1 | | | | | | | | | | |
| Source | electrode | conflict | Type III Sum of Squares | df | Mean Square | F | Sig. | Partial Eta Squared | Noncent. Parameter | Observed Power^a^ |
| electrode | Linear |  | 48.243 | 1 | 48.243 | 3.164 | .084 | .081 | 3.164 | .410 |
| electrode * power | Linear |  | 2.416 | 1 | 2.416 | .158 | .693 | .004 | .158 | .067 |
| Error(electrode) | Linear |  | 548.849 | 36 | 15.246 |  |  |  |  |  |
| conflict |  | Linear | .077 | 1 | .077 | .191 | .665 | .005 | .191 | .071 |
| conflict * power |  | Linear | .156 | 1 | .156 | .386 | .538 | .011 | .386 | .093 |
| Error(conflict) |  | Linear | 14.510 | 36 | .403 |  |  |  |  |  |
| electrode * conflict | Linear | Linear | .008 | 1 | .008 | .036 | .851 | .001 | .036 | .054 |
| electrode * conflict * power | Linear | Linear | .149 | 1 | .149 | .643 | .428 | .018 | .643 | .122 |
| Error(electrode*conflict) | Linear | Linear | 8.345 | 36 | .232 |  |  |  |  |  |
| a. Computed using alpha = .05 | | | | | | | | | | |

| **Levene's Test of Equality of Error Variances^a^** | | | | |
| --- | --- | --- | --- | --- |
|  | F | df1 | df2 | Sig. |
| P7congruent | 8.766 | 1 | 36 | .005 |
| P7incongruent | 6.804 | 1 | 36 | .013 |
| P8congruent | 3.752 | 1 | 36 | .061 |
| P8incongruent | 2.344 | 1 | 36 | .134 |
| Tests the null hypothesis that the error variance of the dependent variable is equal across groups. | | | | |
| a. Design: + power  Within Subjects Design: electrode + conflict + electrode * conflict | | | | |

| **Tests of Between-Subjects Effects** | | | | | | | | |
| --- | --- | --- | --- | --- | --- | --- | --- | --- |
| Measure: MEASURE_1 | | | | | | | | |
| Transformed Variable: Average | | | | | | | | |
| Source | Type III Sum of Squares | df | Mean Square | F | Sig. | Partial Eta Squared | Noncent. Parameter | Observed Power^a^ |
|  | 2673.290 | 1 | 2673.290 | 35.773 | .000 | .498 | 35.773 | 1.000 |
| power | 245.942 | 1 | 245.942 | 3.291 | .078 | .084 | 3.291 | .423 |
| Error | 2690.228 | 36 | 74.729 |  |  |  |  |  |
| a. Computed using alpha = .05 | | | | | | | | |

**Estimated Marginal Means**

**1. power**

| **Estimates** | | | | |
| --- | --- | --- | --- | --- |
| Measure: MEASURE_1 | | | | |
| power | Mean | Std. Error | 95% Confidence Interval | |
|  |  |  | Lower Bound | Upper Bound |
| 1.00 | -5.466 | .992 | -7.477 | -3.455 |
| 2.00 | -2.922 | .992 | -4.933 | -.911 |

| **Pairwise Comparisons** | | | | | | |
| --- | --- | --- | --- | --- | --- | --- |
| Measure: MEASURE_1 | | | | | | |
| (I) power | (J) power | Mean Difference (I-J) | Std. Error | Sig.^a^ | 95% Confidence Interval for Difference^a^ | |
|  |  |  |  |  | Lower Bound | Upper Bound |
| 1.00 | 2.00 | -2.544 | 1.402 | .078 | -5.388 | .300 |
| 2.00 | 1.00 | 2.544 | 1.402 | .078 | -.300 | 5.388 |
| Based on estimated marginal means | | | | | | |
| a. Adjustment for multiple comparisons: Least Significant Difference (equivalent to no adjustments). | | | | | | |

| **Univariate Tests** | | | | | | | | |
| --- | --- | --- | --- | --- | --- | --- | --- | --- |
| Measure: MEASURE_1 | | | | | | | | |
|  | Sum of Squares | df | Mean Square | F | Sig. | Partial Eta Squared | Noncent. Parameter | Observed Power^a^ |
| Contrast | 61.486 | 1 | 61.486 | 3.291 | .078 | .084 | 3.291 | .423 |
| Error | 672.557 | 36 | 18.682 |  |  |  |  |  |
| The F tests the effect of power. This test is based on the linearly independent pairwise comparisons among the estimated marginal means. | | | | | | | | |
| a. Computed using alpha = .05 | | | | | | | | |

**2. electrode**

| **Estimates** | | | | |
| --- | --- | --- | --- | --- |
| Measure: MEASURE_1 | | | | |
| electrode | Mean | Std. Error | 95% Confidence Interval | |
|  |  |  | Lower Bound | Upper Bound |
| 1 | -3.630 | .624 | -4.896 | -2.365 |
| 2 | -4.757 | .891 | -6.565 | -2.949 |

| **Pairwise Comparisons** | | | | | | |
| --- | --- | --- | --- | --- | --- | --- |
| Measure: MEASURE_1 | | | | | | |
| (I) electrode | (J) electrode | Mean Difference (I-J) | Std. Error | Sig.^a^ | 95% Confidence Interval for Difference^a^ | |
|  |  |  |  |  | Lower Bound | Upper Bound |
| 1 | 2 | 1.127 | .633 | .084 | -.158 | 2.411 |
| 2 | 1 | -1.127 | .633 | .084 | -2.411 | .158 |
| Based on estimated marginal means | | | | | | |
| a. Adjustment for multiple comparisons: Least Significant Difference (equivalent to no adjustments). | | | | | | |

| **Multivariate Tests** | | | | | | | | |
| --- | --- | --- | --- | --- | --- | --- | --- | --- |
|  | Value | F | Hypothesis df | Error df | Sig. | Partial Eta Squared | Noncent. Parameter | Observed Power^b^ |
| Pillai's trace | .081 | 3.164^a^ | 1.000 | 36.000 | .084 | .081 | 3.164 | .410 |
| Wilks' lambda | .919 | 3.164^a^ | 1.000 | 36.000 | .084 | .081 | 3.164 | .410 |
| Hotelling's trace | .088 | 3.164^a^ | 1.000 | 36.000 | .084 | .081 | 3.164 | .410 |
| Roy's largest root | .088 | 3.164^a^ | 1.000 | 36.000 | .084 | .081 | 3.164 | .410 |
| Each F tests the multivariate effect of electrode. These tests are based on the linearly independent pairwise comparisons among the estimated marginal means. | | | | | | | | |
| a. Exact statistic | | | | | | | | |
| b. Computed using alpha = .05 | | | | | | | | |

**3. conflict**

| **Estimates** | | | | |
| --- | --- | --- | --- | --- |
| Measure: MEASURE_1 | | | | |
| conflict | Mean | Std. Error | 95% Confidence Interval | |
|  |  |  | Lower Bound | Upper Bound |
| 1 | -4.216 | .700 | -5.636 | -2.796 |
| 2 | -4.171 | .706 | -5.603 | -2.739 |

| **Pairwise Comparisons** | | | | | | |
| --- | --- | --- | --- | --- | --- | --- |
| Measure: MEASURE_1 | | | | | | |
| (I) conflict | (J) conflict | Mean Difference (I-J) | Std. Error | Sig.^a^ | 95% Confidence Interval for Difference^a^ | |
|  |  |  |  |  | Lower Bound | Upper Bound |
| 1 | 2 | -.045 | .103 | .665 | -.254 | .164 |
| 2 | 1 | .045 | .103 | .665 | -.164 | .254 |
| Based on estimated marginal means | | | | | | |
| a. Adjustment for multiple comparisons: Least Significant Difference (equivalent to no adjustments). | | | | | | |

| **Multivariate Tests** | | | | | | | | |
| --- | --- | --- | --- | --- | --- | --- | --- | --- |
|  | Value | F | Hypothesis df | Error df | Sig. | Partial Eta Squared | Noncent. Parameter | Observed Power^b^ |
| Pillai's trace | .005 | .191^a^ | 1.000 | 36.000 | .665 | .005 | .191 | .071 |
| Wilks' lambda | .995 | .191^a^ | 1.000 | 36.000 | .665 | .005 | .191 | .071 |
| Hotelling's trace | .005 | .191^a^ | 1.000 | 36.000 | .665 | .005 | .191 | .071 |
| Roy's largest root | .005 | .191^a^ | 1.000 | 36.000 | .665 | .005 | .191 | .071 |
| Each F tests the multivariate effect of conflict. These tests are based on the linearly independent pairwise comparisons among the estimated marginal means. | | | | | | | | |
| a. Exact statistic | | | | | | | | |
| b. Computed using alpha = .05 | | | | | | | | |

| **4. power * electrode** | | | | | |
| --- | --- | --- | --- | --- | --- |
| Measure: MEASURE_1 | | | | | |
| power | electrode | Mean | Std. Error | 95% Confidence Interval | |
|  |  |  |  | Lower Bound | Upper Bound |
| 1.00 | 1 | -4.776 | .882 | -6.565 | -2.987 |
|  | 2 | -6.155 | 1.261 | -8.712 | -3.598 |
| 2.00 | 1 | -2.484 | .882 | -4.274 | -.695 |
|  | 2 | -3.359 | 1.261 | -5.916 | -.802 |

| **5. power * conflict** | | | | | |
| --- | --- | --- | --- | --- | --- |
| Measure: MEASURE_1 | | | | | |
| power | conflict | Mean | Std. Error | 95% Confidence Interval | |
|  |  |  |  | Lower Bound | Upper Bound |
| 1.00 | 1 | -5.520 | .990 | -7.528 | -3.512 |
|  | 2 | -5.411 | .998 | -7.436 | -3.386 |
| 2.00 | 1 | -2.912 | .990 | -4.920 | -.904 |
|  | 2 | -2.931 | .998 | -4.956 | -.906 |

| **6. electrode * conflict** | | | | | |
| --- | --- | --- | --- | --- | --- |
| Measure: MEASURE_1 | | | | | |
| electrode | conflict | Mean | Std. Error | 95% Confidence Interval | |
|  |  |  |  | Lower Bound | Upper Bound |
| 1 | 1 | -3.645 | .626 | -4.915 | -2.376 |
|  | 2 | -3.615 | .626 | -4.886 | -2.345 |
| 2 | 1 | -4.787 | .887 | -6.586 | -2.988 |
|  | 2 | -4.727 | .902 | -6.556 | -2.898 |

| **7. power * electrode * conflict** | | | | | | |
| --- | --- | --- | --- | --- | --- | --- |
| Measure: MEASURE_1 | | | | | | |
| power | electrode | conflict | Mean | Std. Error | 95% Confidence Interval | |
|  |  |  |  |  | Lower Bound | Upper Bound |
| 1.00 | 1 | 1 | -4.792 | .885 | -6.587 | -2.997 |
|  |  | 2 | -4.761 | .886 | -6.557 | -2.964 |
|  | 2 | 1 | -6.248 | 1.254 | -8.793 | -3.704 |
|  |  | 2 | -6.062 | 1.275 | -8.649 | -3.475 |
| 2.00 | 1 | 1 | -2.499 | .885 | -4.294 | -.704 |
|  |  | 2 | -2.470 | .886 | -4.267 | -.673 |
|  | 2 | 1 | -3.326 | 1.254 | -5.870 | -.781 |
|  |  | 2 | -3.392 | 1.275 | -5.979 | -.806 |

**All the faces in N450**

GLM F3 congruent F3incongruent F4 congruent F4incongruent FZ congruent FZ incongruent C3 congruent C3incongruent C4 congruent C4incongruent CZ congruent CZ incongruent CP1 congruent CP1incongruent CP2 congruent CP2incongruent BY power

/WSFACTOR=electrode 8 Polynomial conflict 2 Polynomial

/METHOD=SSTYPE(3)

/EMMEANS=TABLES(power) COMPARE ADJ(LSD)

/EMMEANS=TABLES(electrode) COMPARE ADJ(LSD)

/EMMEANS=TABLES(conflict) COMPARE ADJ(LSD)

/EMMEANS=TABLES(power*electrode)

/EMMEANS=TABLES(power*conflict)

/EMMEANS=TABLES(electrode*conflict)

/EMMEANS=TABLES(power*electrode*conflict)

/PRINT=DESCRIPTIVE ETASQ OPOWER HOMOGENEITY

/CRITERIA=ALPHA(.05)

/WSDESIGN=electrode conflict electrode*conflict

/DESIGN=power.

**General Linear Model**

| **Notes** | | |
| --- | --- | --- |
| Output Created | | 26-DEC-2020 10:22:09 |
| Comments | |  |
| Input | Data | C:\Users\lenovo\Desktop\ERP data\N450 350-450.sav |
|  | Active Dataset | Dataset |
|  | Filter | <none> |
|  | Weight | <none> |
|  | Split File | <none> |
|  | N of Rows in Working Data File | 41 |
| Missing Value Handling | Definition of Missing | User-defined missing values are treated as missing. |
|  | Cases Used | Statistics are based on all cases with valid data for all variables in the model. |
| Syntax | | GLM F3 congruent F3incongruent F4 congruent F4incongruent FZ congruent FZ incongruent C3 congruent C3incongruent C4 congruent C4incongruent CZ congruent CZ incongruent CP1 congruent CP1incongruent CP2 congruent CP2incongruent BY power  /WSFACTOR=electrode 8 Polynomial conflict 2 Polynomial  /METHOD=SSTYPE(3)  /EMMEANS=TABLES(power) COMPARE ADJ(LSD)  /EMMEANS=TABLES(electrode) COMPARE ADJ(LSD)  /EMMEANS=TABLES(conflict) COMPARE ADJ(LSD)  /EMMEANS=TABLES(power*electrode)  /EMMEANS=TABLES(power*conflict)  /EMMEANS=TABLES(electrode*conflict)  /EMMEANS=TABLES(power*electrode*conflict)  /PRINT=DESCRIPTIVE ETASQ OPOWER HOMOGENEITY  /CRITERIA=ALPHA(.05)  /WSDESIGN=electrode conflict electrode*conflict  /DESIGN=power. |
| Resources | Processor Time | 00:00:00.06 |
|  | Elapsed Time | 00:00:00.07 |

| **Within-Subjects Factors** | | |
| --- | --- | --- |
| Measure: MEASURE_1 | | |
| electrode | conflict | Dependent Variable |
| 1 | 1 | F3congruent |
|  | 2 | F3incongruent |
| 2 | 1 | F4congruent |
|  | 2 | F4incongruent |
| 3 | 1 | FZ congruent |
|  | 2 | FZ incongruent |
| 4 | 1 | C3congruent |
|  | 2 | C3incongruent |
| 5 | 1 | C4congruent |
|  | 2 | C4incongruent |
| 6 | 1 | CZ congruent |
|  | 2 | CZ incongruent |
| 7 | 1 | CP1congruent |
|  | 2 | CP1incongruent |
| 8 | 1 | CP2congruent |
|  | 2 | CP2incongruent |

| **Between-Subjects Factors** | | |
| --- | --- | --- |
|  | | N |
| power | 1.00 | 19 |
|  | 2.00 | 19 |

| **Descriptive Statistics** | | | | |
| --- | --- | --- | --- | --- |
|  | power | Mean | Std. Deviation | N |
| F3congruent | 1.00 | 1.1241 | 4.43022 | 19 |
|  | 2.00 | 1.7813 | 3.20004 | 19 |
|  | Total | 1.4527 | 3.82633 | 38 |
| F3incongruent | 1.00 | .9839 | 4.76434 | 19 |
|  | 2.00 | .8932 | 2.79447 | 19 |
|  | Total | .9385 | 3.85277 | 38 |
| F4congruent | 1.00 | 2.6366 | 5.09201 | 19 |
|  | 2.00 | 2.0605 | 3.11588 | 19 |
|  | Total | 2.3485 | 4.17400 | 38 |
| F4incongruent | 1.00 | 2.1761 | 5.40926 | 19 |
|  | 2.00 | 1.5428 | 2.87978 | 19 |
|  | Total | 1.8594 | 4.28627 | 38 |
| FZ congruent | 1.00 | 1.7994 | 5.12743 | 19 |
|  | 2.00 | 1.5776 | 3.16534 | 19 |
|  | Total | 1.6885 | 4.20439 | 38 |
| FZ incongruent | 1.00 | 1.4039 | 5.56883 | 19 |
|  | 2.00 | .8152 | 2.79597 | 19 |
|  | Total | 1.1096 | 4.35648 | 38 |
| C3congruent | 1.00 | 3.0009 | 2.31086 | 19 |
|  | 2.00 | 3.4184 | 3.20490 | 19 |
|  | Total | 3.2097 | 2.76397 | 38 |
| C3incongruent | 1.00 | 2.8297 | 2.68097 | 19 |
|  | 2.00 | 2.6436 | 2.59058 | 19 |
|  | Total | 2.7366 | 2.60200 | 38 |
| C4congruent | 1.00 | 4.9238 | 3.39283 | 19 |
|  | 2.00 | 4.6019 | 2.46885 | 19 |
|  | Total | 4.7629 | 2.93120 | 38 |
| C4incongruent | 1.00 | 4.4081 | 3.71080 | 19 |
|  | 2.00 | 3.7172 | 2.28335 | 19 |
|  | Total | 4.0627 | 3.05906 | 38 |
| CZ congruent | 1.00 | 4.3775 | 3.59840 | 19 |
|  | 2.00 | 4.1266 | 3.47152 | 19 |
|  | Total | 4.2520 | 3.48974 | 38 |
| CZ incongruent | 1.00 | 3.7990 | 3.66078 | 19 |
|  | 2.00 | 3.4237 | 3.63388 | 19 |
|  | Total | 3.6113 | 3.60275 | 38 |
| CP1congruent | 1.00 | 5.6638 | 3.14717 | 19 |
|  | 2.00 | 6.1359 | 3.25863 | 19 |
|  | Total | 5.8999 | 3.16884 | 38 |
| CP1incongruent | 1.00 | 5.5454 | 3.28313 | 19 |
|  | 2.00 | 5.4335 | 3.23593 | 19 |
|  | Total | 5.4894 | 3.21577 | 38 |
| CP2congruent | 1.00 | 6.9287 | 3.62285 | 19 |
|  | 2.00 | 6.7323 | 3.04865 | 19 |
|  | Total | 6.8305 | 3.30403 | 38 |
| CP2incongruent | 1.00 | 6.5501 | 3.97514 | 19 |
|  | 2.00 | 6.0303 | 3.17422 | 19 |
|  | Total | 6.2902 | 3.55786 | 38 |

| **Box's Test of Equality of Covariance Matrices^a^** | |
| --- | --- |
| Box's M | 357.649 |
| F | 1.339 |
| df1 | 136 |
| df2 | 4002.174 |
| Sig. | .006 |
| Tests the null hypothesis that the observed covariance matrices of the dependent variables are equal across groups. | |
| a. Design: + power  Within Subjects Design: electrode + conflict + electrode * conflict | |

| **Multivariate Tests^a^** | | | | | | | | | |
| --- | --- | --- | --- | --- | --- | --- | --- | --- | --- |
| Effect | | Value | F | Hypothesis df | Error df | Sig. | Partial Eta Squared | Noncent. Parameter | Observed Power^c^ |
| electrode | Pillai's Trace | .784 | 15.547^b^ | 7.000 | 30.000 | .000 | .784 | 108.828 | 1.000 |
|  | Wilks' Lambda | .216 | 15.547^b^ | 7.000 | 30.000 | .000 | .784 | 108.828 | 1.000 |
|  | Hotelling's Trace | 3.628 | 15.547^b^ | 7.000 | 30.000 | .000 | .784 | 108.828 | 1.000 |
|  | Roy's Largest Root | 3.628 | 15.547^b^ | 7.000 | 30.000 | .000 | .784 | 108.828 | 1.000 |
| electrode * power | Pillai's Trace | .063 | .289^b^ | 7.000 | 30.000 | .953 | .063 | 2.026 | .119 |
|  | Wilks' Lambda | .937 | .289^b^ | 7.000 | 30.000 | .953 | .063 | 2.026 | .119 |
|  | Hotelling's Trace | .068 | .289^b^ | 7.000 | 30.000 | .953 | .063 | 2.026 | .119 |
|  | Roy's Largest Root | .068 | .289^b^ | 7.000 | 30.000 | .953 | .063 | 2.026 | .119 |
| conflict | Pillai's Trace | .141 | 5.915^b^ | 1.000 | 36.000 | .020 | .141 | 5.915 | .658 |
|  | Wilks' Lambda | .859 | 5.915^b^ | 1.000 | 36.000 | .020 | .141 | 5.915 | .658 |
|  | Hotelling's Trace | .164 | 5.915^b^ | 1.000 | 36.000 | .020 | .141 | 5.915 | .658 |
|  | Roy's Largest Root | .164 | 5.915^b^ | 1.000 | 36.000 | .020 | .141 | 5.915 | .658 |
| conflict * power | Pillai's Trace | .021 | .790^b^ | 1.000 | 36.000 | .380 | .021 | .790 | .139 |
|  | Wilks' Lambda | .979 | .790^b^ | 1.000 | 36.000 | .380 | .021 | .790 | .139 |
|  | Hotelling's Trace | .022 | .790^b^ | 1.000 | 36.000 | .380 | .021 | .790 | .139 |
|  | Roy's Largest Root | .022 | .790^b^ | 1.000 | 36.000 | .380 | .021 | .790 | .139 |
| electrode * conflict | Pillai's Trace | .401 | 2.864^b^ | 7.000 | 30.000 | .021 | .401 | 20.045 | .849 |
|  | Wilks' Lambda | .599 | 2.864^b^ | 7.000 | 30.000 | .021 | .401 | 20.045 | .849 |
|  | Hotelling's Trace | .668 | 2.864^b^ | 7.000 | 30.000 | .021 | .401 | 20.045 | .849 |
|  | Roy's Largest Root | .668 | 2.864^b^ | 7.000 | 30.000 | .021 | .401 | 20.045 | .849 |
| electrode * conflict * power | Pillai's Trace | .282 | 1.681^b^ | 7.000 | 30.000 | .152 | .282 | 11.766 | .584 |
|  | Wilks' Lambda | .718 | 1.681^b^ | 7.000 | 30.000 | .152 | .282 | 11.766 | .584 |
|  | Hotelling's Trace | .392 | 1.681^b^ | 7.000 | 30.000 | .152 | .282 | 11.766 | .584 |
|  | Roy's Largest Root | .392 | 1.681^b^ | 7.000 | 30.000 | .152 | .282 | 11.766 | .584 |
| a. Design: + power  Within Subjects Design: electrode + conflict + electrode * conflict | | | | | | | | | |
| b. Exact statistic | | | | | | | | | |
| c. Computed using alpha = .05 | | | | | | | | | |

| **Mauchly's Test of Sphericity^a^** | | | | | | | |
| --- | --- | --- | --- | --- | --- | --- | --- |
| Measure: MEASURE_1 | | | | | | | |
| Within Subjects Effect | Mauchly's W | Approx. Chi-Square | df | Sig. | Epsilon^b^ | | |
|  |  |  |  |  | Greenhouse-Geisser | Huynh-Feldt | Lower-bound |
| electrode | .000 | 275.876 | 27 | .000 | .291 | .317 | .143 |
| conflict | 1.000 | .000 | 0 | . | 1.000 | 1.000 | 1.000 |
| electrode * conflict | .052 | 99.191 | 27 | .000 | .494 | .569 | .143 |
| Tests the null hypothesis that the error covariance matrix of the orthonormalized transformed dependent variables is proportional to an identity matrix. | | | | | | | |
| a. Design: + power  Within Subjects Design: electrode + conflict + electrode * conflict | | | | | | | |
| b. May be used to adjust the degrees of freedom for the averaged tests of significance. Corrected tests are displayed in the Tests of Within-Subjects Effects table. | | | | | | | |

| **Tests of Within-Subjects Effects** | | | | | | | | | |
| --- | --- | --- | --- | --- | --- | --- | --- | --- | --- |
| Measure: MEASURE_1 | | | | | | | | | |
| Source | | Type III Sum of Squares | df | Mean Square | F | Sig. | Partial Eta Squared | Noncent. Parameter | Observed Power^a^ |
| electrode | Sphericity Assumed | 2062.884 | 7 | 294.698 | 29.809 | .000 | .453 | 208.663 | 1.000 |
|  | Greenhouse-Geisser | 2062.884 | 2.037 | 1012.867 | 29.809 | .000 | .453 | 60.711 | 1.000 |
|  | Huynh-Feldt | 2062.884 | 2.220 | 929.287 | 29.809 | .000 | .453 | 66.172 | 1.000 |
|  | Lower-bound | 2062.884 | 1.000 | 2062.884 | 29.809 | .000 | .453 | 29.809 | 1.000 |
| electrode * power | Sphericity Assumed | 15.490 | 7 | 2.213 | .224 | .980 | .006 | 1.567 | .114 |
|  | Greenhouse-Geisser | 15.490 | 2.037 | 7.606 | .224 | .804 | .006 | .456 | .084 |
|  | Huynh-Feldt | 15.490 | 2.220 | 6.978 | .224 | .822 | .006 | .497 | .085 |
|  | Lower-bound | 15.490 | 1.000 | 15.490 | .224 | .639 | .006 | .224 | .075 |
| Error(electrode) | Sphericity Assumed | 2491.326 | 252 | 9.886 |  |  |  |  |  |
|  | Greenhouse-Geisser | 2491.326 | 73.320 | 33.979 |  |  |  |  |  |
|  | Huynh-Feldt | 2491.326 | 79.915 | 31.175 |  |  |  |  |  |
|  | Lower-bound | 2491.326 | 36.000 | 69.203 |  |  |  |  |  |
| conflict | Sphericity Assumed | 44.877 | 1 | 44.877 | 5.915 | .020 | .141 | 5.915 | .658 |
|  | Greenhouse-Geisser | 44.877 | 1.000 | 44.877 | 5.915 | .020 | .141 | 5.915 | .658 |
|  | Huynh-Feldt | 44.877 | 1.000 | 44.877 | 5.915 | .020 | .141 | 5.915 | .658 |
|  | Lower-bound | 44.877 | 1.000 | 44.877 | 5.915 | .020 | .141 | 5.915 | .658 |
| conflict * power | Sphericity Assumed | 5.990 | 1 | 5.990 | .790 | .380 | .021 | .790 | .139 |
|  | Greenhouse-Geisser | 5.990 | 1.000 | 5.990 | .790 | .380 | .021 | .790 | .139 |
|  | Huynh-Feldt | 5.990 | 1.000 | 5.990 | .790 | .380 | .021 | .790 | .139 |
|  | Lower-bound | 5.990 | 1.000 | 5.990 | .790 | .380 | .021 | .790 | .139 |
| Error(conflict) | Sphericity Assumed | 273.110 | 36 | 7.586 |  |  |  |  |  |
|  | Greenhouse-Geisser | 273.110 | 36.000 | 7.586 |  |  |  |  |  |
|  | Huynh-Feldt | 273.110 | 36.000 | 7.586 |  |  |  |  |  |
|  | Lower-bound | 273.110 | 36.000 | 7.586 |  |  |  |  |  |
| electrode * conflict | Sphericity Assumed | 1.173 | 7 | .168 | 1.046 | .400 | .028 | 7.321 | .448 |
|  | Greenhouse-Geisser | 1.173 | 3.461 | .339 | 1.046 | .381 | .028 | 3.620 | .299 |
|  | Huynh-Feldt | 1.173 | 3.980 | .295 | 1.046 | .386 | .028 | 4.163 | .323 |
|  | Lower-bound | 1.173 | 1.000 | 1.173 | 1.046 | .313 | .028 | 1.046 | .169 |
| electrode * conflict * power | Sphericity Assumed | 1.889 | 7 | .270 | 1.683 | .113 | .045 | 11.784 | .686 |
|  | Greenhouse-Geisser | 1.889 | 3.461 | .546 | 1.683 | .166 | .045 | 5.826 | .467 |
|  | Huynh-Feldt | 1.889 | 3.980 | .474 | 1.683 | .157 | .045 | 6.701 | .505 |
|  | Lower-bound | 1.889 | 1.000 | 1.889 | 1.683 | .203 | .045 | 1.683 | .244 |
| Error(electrode*conflict) | Sphericity Assumed | 40.390 | 252 | .160 |  |  |  |  |  |
|  | Greenhouse-Geisser | 40.390 | 124.598 | .324 |  |  |  |  |  |
|  | Huynh-Feldt | 40.390 | 143.297 | .282 |  |  |  |  |  |
|  | Lower-bound | 40.390 | 36.000 | 1.122 |  |  |  |  |  |
| a. Computed using alpha = .05 | | | | | | | | | |

| **Tests of Within-Subjects Contrasts** | | | | | | | | | | |
| --- | --- | --- | --- | --- | --- | --- | --- | --- | --- | --- |
| Measure: MEASURE_1 | | | | | | | | | | |
| Source | electrode | conflict | Type III Sum of Squares | df | Mean Square | F | Sig. | Partial Eta Squared | Noncent. Parameter | Observed Power^a^ |
| electrode | Linear |  | 1884.586 | 1 | 1884.586 | 45.535 | .000 | .558 | 45.535 | 1.000 |
|  | Quadratic |  | 38.030 | 1 | 38.030 | 40.961 | .000 | .532 | 40.961 | 1.000 |
|  | Cubic |  | 1.725 | 1 | 1.725 | .411 | .526 | .011 | .411 | .096 |
|  | Order 4 |  | 1.418 | 1 | 1.418 | .359 | .553 | .010 | .359 | .090 |
|  | Order 5 |  | 13.391 | 1 | 13.391 | 1.586 | .216 | .042 | 1.586 | .232 |
|  | Order 6 |  | 117.356 | 1 | 117.356 | 19.176 | .000 | .348 | 19.176 | .989 |
|  | Order 7 |  | 6.378 | 1 | 6.378 | 1.525 | .225 | .041 | 1.525 | .225 |
| electrode * power | Linear |  | .094 | 1 | .094 | .002 | .962 | .000 | .002 | .050 |
|  | Quadratic |  | 1.129 | 1 | 1.129 | 1.216 | .277 | .033 | 1.216 | .189 |
|  | Cubic |  | 3.723 | 1 | 3.723 | .887 | .353 | .024 | .887 | .150 |
|  | Order 4 |  | .408 | 1 | .408 | .103 | .750 | .003 | .103 | .061 |
|  | Order 5 |  | 7.989 | 1 | 7.989 | .946 | .337 | .026 | .946 | .157 |
|  | Order 6 |  | .437 | 1 | .437 | .071 | .791 | .002 | .071 | .058 |
|  | Order 7 |  | 1.710 | 1 | 1.710 | .409 | .527 | .011 | .409 | .095 |
| Error(electrode) | Linear |  | 1489.942 | 36 | 41.387 |  |  |  |  |  |
|  | Quadratic |  | 33.424 | 36 | .928 |  |  |  |  |  |
|  | Cubic |  | 151.109 | 36 | 4.197 |  |  |  |  |  |
|  | Order 4 |  | 142.102 | 36 | 3.947 |  |  |  |  |  |
|  | Order 5 |  | 303.914 | 36 | 8.442 |  |  |  |  |  |
|  | Order 6 |  | 220.312 | 36 | 6.120 |  |  |  |  |  |
|  | Order 7 |  | 150.522 | 36 | 4.181 |  |  |  |  |  |
| conflict |  | Linear | 44.877 | 1 | 44.877 | 5.915 | .020 | .141 | 5.915 | .658 |
| conflict * power |  | Linear | 5.990 | 1 | 5.990 | .790 | .380 | .021 | .790 | .139 |
| Error(conflict) |  | Linear | 273.110 | 36 | 7.586 |  |  |  |  |  |
| electrode * conflict | Linear | Linear | .005 | 1 | .005 | .010 | .920 | .000 | .010 | .051 |
|  | Quadratic | Linear | .175 | 1 | .175 | 1.839 | .184 | .049 | 1.839 | .262 |
|  | Cubic | Linear | .021 | 1 | .021 | .219 | .643 | .006 | .219 | .074 |
|  | Order 4 | Linear | .207 | 1 | .207 | 1.999 | .166 | .053 | 1.999 | .280 |
|  | Order 5 | Linear | .362 | 1 | .362 | 1.915 | .175 | .051 | 1.915 | .270 |
|  | Order 6 | Linear | .200 | 1 | .200 | 1.900 | .177 | .050 | 1.900 | .269 |
|  | Order 7 | Linear | .204 | 1 | .204 | 2.399 | .130 | .062 | 2.399 | .326 |
| electrode * conflict * power | Linear | Linear | .048 | 1 | .048 | .106 | .747 | .003 | .106 | .062 |
|  | Quadratic | Linear | .092 | 1 | .092 | .965 | .332 | .026 | .965 | .160 |
|  | Cubic | Linear | .184 | 1 | .184 | 1.942 | .172 | .051 | 1.942 | .274 |
|  | Order 4 | Linear | .320 | 1 | .320 | 3.101 | .087 | .079 | 3.101 | .403 |
|  | Order 5 | Linear | 1.124 | 1 | 1.124 | 5.944 | .020 | .142 | 5.944 | .660 |
|  | Order 6 | Linear | .119 | 1 | .119 | 1.134 | .294 | .031 | 1.134 | .179 |
|  | Order 7 | Linear | .001 | 1 | .001 | .016 | .900 | .000 | .016 | .052 |
| Error(electrode*conflict) | Linear | Linear | 16.161 | 36 | .449 |  |  |  |  |  |
|  | Quadratic | Linear | 3.429 | 36 | .095 |  |  |  |  |  |
|  | Cubic | Linear | 3.419 | 36 | .095 |  |  |  |  |  |
|  | Order 4 | Linear | 3.719 | 36 | .103 |  |  |  |  |  |
|  | Order 5 | Linear | 6.805 | 36 | .189 |  |  |  |  |  |
|  | Order 6 | Linear | 3.789 | 36 | .105 |  |  |  |  |  |
|  | Order 7 | Linear | 3.068 | 36 | .085 |  |  |  |  |  |
| a. Computed using alpha = .05 | | | | | | | | | | |

| **Levene's Test of Equality of Error Variances^a^** | | | | |
| --- | --- | --- | --- | --- |
|  | F | df1 | df2 | Sig. |
| F3congruent | 2.088 | 1 | 36 | .157 |
| F3incongruent | 4.900 | 1 | 36 | .033 |
| F4congruent | 4.004 | 1 | 36 | .053 |
| F4incongruent | 7.878 | 1 | 36 | .008 |
| FZ congruent | 5.757 | 1 | 36 | .022 |
| FZ incongruent | 11.279 | 1 | 36 | .002 |
| C3congruent | 3.372 | 1 | 36 | .075 |
| C3incongruent | .128 | 1 | 36 | .723 |
| C4congruent | 2.304 | 1 | 36 | .138 |
| C4incongruent | 6.970 | 1 | 36 | .012 |
| CZcongruent | .212 | 1 | 36 | .648 |
| CZincongruent | .463 | 1 | 36 | .501 |
| CP1congruent | .531 | 1 | 36 | .471 |
| CP1incongruent | .215 | 1 | 36 | .645 |
| CP2congruent | .017 | 1 | 36 | .898 |
| CP2incongruent | .633 | 1 | 36 | .431 |
| Tests the null hypothesis that the error variance of the dependent variable is equal across groups. | | | | |
| a. Design: + power  Within Subjects Design: electrode + conflict + electrode * conflict | | | | |

| **Tests of Between-Subjects Effects** | | | | | | | | |
| --- | --- | --- | --- | --- | --- | --- | --- | --- |
| Measure: MEASURE_1 | | | | | | | | |
| Transformed Variable: Average | | | | | | | | |
| Source | Type III Sum of Squares | df | Mean Square | F | Sig. | Partial Eta Squared | Noncent. Parameter | Observed Power^a^ |
|  | 7592.998 | 1 | 7592.998 | 58.260 | .000 | .618 | 58.260 | 1.000 |
| power | 6.144 | 1 | 6.144 | .047 | .829 | .001 | .047 | .055 |
| Error | 4691.846 | 36 | 130.329 |  |  |  |  |  |
| a. Computed using alpha = .05 | | | | | | | | |

**Estimated Marginal Means**

**1. power**

| **Estimates** | | | | |
| --- | --- | --- | --- | --- |
| Measure: MEASURE_1 | | | | |
| power | Mean | Std. Error | 95% Confidence Interval | |
|  |  |  | Lower Bound | Upper Bound |
| 1.00 | 3.634 | .655 | 2.307 | 4.962 |
| 2.00 | 3.433 | .655 | 2.105 | 4.761 |

| **Pairwise Comparisons** | | | | | | |
| --- | --- | --- | --- | --- | --- | --- |
| Measure: MEASURE_1 | | | | | | |
| (I) power | (J) power | Mean Difference (I-J) | Std. Error | Sig.^a^ | 95% Confidence Interval for Difference^a^ | |
|  |  |  |  |  | Lower Bound | Upper Bound |
| 1.00 | 2.00 | .201 | .926 | .829 | -1.677 | 2.079 |
| 2.00 | 1.00 | -.201 | .926 | .829 | -2.079 | 1.677 |
| Based on estimated marginal means | | | | | | |
| a. Adjustment for multiple comparisons: Least Significant Difference (equivalent to no adjustments). | | | | | | |

| **Univariate Tests** | | | | | | | | |
| --- | --- | --- | --- | --- | --- | --- | --- | --- |
| Measure: MEASURE_1 | | | | | | | | |
|  | Sum of Squares | df | Mean Square | F | Sig. | Partial Eta Squared | Noncent. Parameter | Observed Power^a^ |
| Contrast | .384 | 1 | .384 | .047 | .829 | .001 | .047 | .055 |
| Error | 293.240 | 36 | 8.146 |  |  |  |  |  |
| The F tests the effect of power. This test is based on the linearly independent pairwise comparisons among the estimated marginal means. | | | | | | | | |
| a. Computed using alpha = .05 | | | | | | | | |

**2. electrode**

| **Estimates** | | | | |
| --- | --- | --- | --- | --- |
| Measure: MEASURE_1 | | | | |
| electrode | Mean | Std. Error | 95% Confidence Interval | |
|  |  |  | Lower Bound | Upper Bound |
| 1 | 1.196 | .619 | -.060 | 2.451 |
| 2 | 2.104 | .684 | .717 | 3.491 |
| 3 | 1.399 | .692 | -.005 | 2.803 |
| 4 | 2.973 | .422 | 2.116 | 3.830 |
| 5 | 4.413 | .478 | 3.444 | 5.382 |
| 6 | 3.932 | .573 | 2.770 | 5.093 |
| 7 | 5.695 | .508 | 4.664 | 6.725 |
| 8 | 6.560 | .550 | 5.445 | 7.676 |

| **Pairwise Comparisons** | | | | | | |
| --- | --- | --- | --- | --- | --- | --- |
| Measure: MEASURE_1 | | | | | | |
| (I) electrode | (J) electrode | Mean Difference (I-J) | Std. Error | Sig.^b^ | 95% Confidence Interval for Difference^b^ | |
|  |  |  |  |  | Lower Bound | Upper Bound |
| 1 | 2 | -.908 | .453 | .052 | -1.826 | .010 |
|  | 3 | -.203 | .301 | .503 | -.814 | .407 |
|  | 4 | -1.778^*^ | .436 | .000 | -2.662 | -.893 |
|  | 5 | -3.217^*^ | .549 | .000 | -4.330 | -2.104 |
|  | 6 | -2.736^*^ | .579 | .000 | -3.910 | -1.562 |
|  | 7 | -4.499^*^ | .688 | .000 | -5.893 | -3.105 |
|  | 8 | -5.365^*^ | .734 | .000 | -6.853 | -3.876 |
| 2 | 1 | .908 | .453 | .052 | -.010 | 1.826 |
|  | 3 | .705^*^ | .289 | .020 | .118 | 1.292 |
|  | 4 | -.869 | .608 | .161 | -2.101 | .363 |
|  | 5 | -2.309^*^ | .449 | .000 | -3.220 | -1.398 |
|  | 6 | -1.828^*^ | .571 | .003 | -2.986 | -.670 |
|  | 7 | -3.591^*^ | .742 | .000 | -5.095 | -2.086 |
|  | 8 | -4.456^*^ | .694 | .000 | -5.864 | -3.049 |
| 3 | 1 | .203 | .301 | .503 | -.407 | .814 |
|  | 2 | -.705^*^ | .289 | .020 | -1.292 | -.118 |
|  | 4 | -1.574^*^ | .546 | .007 | -2.681 | -.467 |
|  | 5 | -3.014^*^ | .518 | .000 | -4.063 | -1.964 |
|  | 6 | -2.533^*^ | .542 | .000 | -3.632 | -1.433 |
|  | 7 | -4.296^*^ | .726 | .000 | -5.768 | -2.823 |
|  | 8 | -5.161^*^ | .724 | .000 | -6.629 | -3.694 |
| 4 | 1 | 1.778^*^ | .436 | .000 | .893 | 2.662 |
|  | 2 | .869 | .608 | .161 | -.363 | 2.101 |
|  | 3 | 1.574^*^ | .546 | .007 | .467 | 2.681 |
|  | 5 | -1.440^*^ | .386 | .001 | -2.223 | -.656 |
|  | 6 | -.959^*^ | .381 | .017 | -1.732 | -.185 |
|  | 7 | -2.722^*^ | .333 | .000 | -3.397 | -2.046 |
|  | 8 | -3.587^*^ | .428 | .000 | -4.454 | -2.720 |
| 5 | 1 | 3.217^*^ | .549 | .000 | 2.104 | 4.330 |
|  | 2 | 2.309^*^ | .449 | .000 | 1.398 | 3.220 |
|  | 3 | 3.014^*^ | .518 | .000 | 1.964 | 4.063 |
|  | 4 | 1.440^*^ | .386 | .001 | .656 | 2.223 |
|  | 6 | .481 | .342 | .168 | -.212 | 1.174 |
|  | 7 | -1.282^*^ | .415 | .004 | -2.124 | -.440 |
|  | 8 | -2.148^*^ | .305 | .000 | -2.766 | -1.529 |
| 6 | 1 | 2.736^*^ | .579 | .000 | 1.562 | 3.910 |
|  | 2 | 1.828^*^ | .571 | .003 | .670 | 2.986 |
|  | 3 | 2.533^*^ | .542 | .000 | 1.433 | 3.632 |
|  | 4 | .959^*^ | .381 | .017 | .185 | 1.732 |
|  | 5 | -.481 | .342 | .168 | -1.174 | .212 |
|  | 7 | -1.763^*^ | .320 | .000 | -2.411 | -1.115 |
|  | 8 | -2.629^*^ | .317 | .000 | -3.272 | -1.985 |
| 7 | 1 | 4.499^*^ | .688 | .000 | 3.105 | 5.893 |
|  | 2 | 3.591^*^ | .742 | .000 | 2.086 | 5.095 |
|  | 3 | 4.296^*^ | .726 | .000 | 2.823 | 5.768 |
|  | 4 | 2.722^*^ | .333 | .000 | 2.046 | 3.397 |
|  | 5 | 1.282^*^ | .415 | .004 | .440 | 2.124 |
|  | 6 | 1.763^*^ | .320 | .000 | 1.115 | 2.411 |
|  | 8 | -.866^*^ | .236 | .001 | -1.345 | -.386 |
| 8 | 1 | 5.365^*^ | .734 | .000 | 3.876 | 6.853 |
|  | 2 | 4.456^*^ | .694 | .000 | 3.049 | 5.864 |
|  | 3 | 5.161^*^ | .724 | .000 | 3.694 | 6.629 |
|  | 4 | 3.587^*^ | .428 | .000 | 2.720 | 4.454 |
|  | 5 | 2.148^*^ | .305 | .000 | 1.529 | 2.766 |
|  | 6 | 2.629^*^ | .317 | .000 | 1.985 | 3.272 |
|  | 7 | .866^*^ | .236 | .001 | .386 | 1.345 |
| Based on estimated marginal means | | | | | | |
| *. The mean difference is significant at the .05 level. | | | | | | |
| b. Adjustment for multiple comparisons: Least Significant Difference (equivalent to no adjustments). | | | | | | |

| **Multivariate Tests** | | | | | | | | |
| --- | --- | --- | --- | --- | --- | --- | --- | --- |
|  | Value | F | Hypothesis df | Error df | Sig. | Partial Eta Squared | Noncent. Parameter | Observed Power^b^ |
| Pillai's trace | .784 | 15.547^a^ | 7.000 | 30.000 | .000 | .784 | 108.828 | 1.000 |
| Wilks' lambda | .216 | 15.547^a^ | 7.000 | 30.000 | .000 | .784 | 108.828 | 1.000 |
| Hotelling's trace | 3.628 | 15.547^a^ | 7.000 | 30.000 | .000 | .784 | 108.828 | 1.000 |
| Roy's largest root | 3.628 | 15.547^a^ | 7.000 | 30.000 | .000 | .784 | 108.828 | 1.000 |
| Each F tests the multivariate effect of electrode. These tests are based on the linearly independent pairwise comparisons among the estimated marginal means. | | | | | | | | |
| a. Exact statistic | | | | | | | | |
| b. Computed using alpha = .05 | | | | | | | | |

**3. conflict**

| **Estimates** | | | | |
| --- | --- | --- | --- | --- |
| Measure: MEASURE_1 | | | | |
| conflict | Mean | Std. Error | 95% Confidence Interval | |
|  |  |  | Lower Bound | Upper Bound |
| 1 | 3.806 | .468 | 2.856 | 4.756 |
| 2 | 3.262 | .484 | 2.281 | 4.244 |

| **Pairwise Comparisons** | | | | | | |
| --- | --- | --- | --- | --- | --- | --- |
| Measure: MEASURE_1 | | | | | | |
| (I) conflict | (J) conflict | Mean Difference (I-J) | Std. Error | Sig.^b^ | 95% Confidence Interval for Difference^b^ | |
|  |  |  |  |  | Lower Bound | Upper Bound |
| 1 | 2 | .543^*^ | .223 | .020 | .090 | .996 |
| 2 | 1 | -.543^*^ | .223 | .020 | -.996 | -.090 |
| Based on estimated marginal means | | | | | | |
| *. The mean difference is significant at the .05 level. | | | | | | |
| b. Adjustment for multiple comparisons: Least Significant Difference (equivalent to no adjustments). | | | | | | |

| **Multivariate Tests** | | | | | | | | |
| --- | --- | --- | --- | --- | --- | --- | --- | --- |
|  | Value | F | Hypothesis df | Error df | Sig. | Partial Eta Squared | Noncent. Parameter | Observed Power^b^ |
| Pillai's trace | .141 | 5.915^a^ | 1.000 | 36.000 | .020 | .141 | 5.915 | .658 |
| Wilks' lambda | .859 | 5.915^a^ | 1.000 | 36.000 | .020 | .141 | 5.915 | .658 |
| Hotelling's trace | .164 | 5.915^a^ | 1.000 | 36.000 | .020 | .141 | 5.915 | .658 |
| Roy's largest root | .164 | 5.915^a^ | 1.000 | 36.000 | .020 | .141 | 5.915 | .658 |
| Each F tests the multivariate effect of conflict. These tests are based on the linearly independent pairwise comparisons among the estimated marginal means. | | | | | | | | |
| a. Exact statistic | | | | | | | | |
| b. Computed using alpha = .05 | | | | | | | | |

| **4. power * electrode** | | | | | |
| --- | --- | --- | --- | --- | --- |
| Measure: MEASURE_1 | | | | | |
| power | electrode | Mean | Std. Error | 95% Confidence Interval | |
|  |  |  |  | Lower Bound | Upper Bound |
| 1.00 | 1 | 1.054 | .875 | -.721 | 2.829 |
|  | 2 | 2.406 | .967 | .445 | 4.368 |
|  | 3 | 1.602 | .979 | -.384 | 3.587 |
|  | 4 | 2.915 | .597 | 1.704 | 4.127 |
|  | 5 | 4.666 | .676 | 3.296 | 6.036 |
|  | 6 | 4.088 | .810 | 2.445 | 5.731 |
|  | 7 | 5.605 | .719 | 4.147 | 7.062 |
|  | 8 | 6.739 | .778 | 5.162 | 8.317 |
| 2.00 | 1 | 1.337 | .875 | -.438 | 3.112 |
|  | 2 | 1.802 | .967 | -.160 | 3.763 |
|  | 3 | 1.196 | .979 | -.789 | 3.182 |
|  | 4 | 3.031 | .597 | 1.820 | 4.242 |
|  | 5 | 4.160 | .676 | 2.790 | 5.530 |
|  | 6 | 3.775 | .810 | 2.132 | 5.418 |
|  | 7 | 5.785 | .719 | 4.327 | 7.242 |
|  | 8 | 6.381 | .778 | 4.804 | 7.959 |

| **5. power * conflict** | | | | | |
| --- | --- | --- | --- | --- | --- |
| Measure: MEASURE_1 | | | | | |
| power | conflict | Mean | Std. Error | 95% Confidence Interval | |
|  |  |  |  | Lower Bound | Upper Bound |
| 1.00 | 1 | 3.807 | .662 | 2.463 | 5.150 |
|  | 2 | 3.462 | .684 | 2.074 | 4.850 |
| 2.00 | 1 | 3.804 | .662 | 2.461 | 5.148 |
|  | 2 | 3.062 | .684 | 1.674 | 4.451 |

| **6. electrode * conflict** | | | | | |
| --- | --- | --- | --- | --- | --- |
| Measure: MEASURE_1 | | | | | |
| electrode | conflict | Mean | Std. Error | 95% Confidence Interval | |
|  |  |  |  | Lower Bound | Upper Bound |
| 1 | 1 | 1.453 | .627 | .181 | 2.724 |
|  | 2 | .939 | .634 | -.346 | 2.224 |
| 2 | 1 | 2.349 | .685 | .960 | 3.737 |
|  | 2 | 1.859 | .703 | .434 | 3.285 |
| 3 | 1 | 1.688 | .691 | .287 | 3.090 |
|  | 2 | 1.110 | .715 | -.340 | 2.559 |
| 4 | 1 | 3.210 | .453 | 2.290 | 4.129 |
|  | 2 | 2.737 | .428 | 1.869 | 3.604 |
| 5 | 1 | 4.763 | .481 | 3.787 | 5.739 |
|  | 2 | 4.063 | .500 | 3.049 | 5.076 |
| 6 | 1 | 4.252 | .574 | 3.089 | 5.415 |
|  | 2 | 3.611 | .592 | 2.411 | 4.811 |
| 7 | 1 | 5.900 | .520 | 4.846 | 6.954 |
|  | 2 | 5.489 | .529 | 4.417 | 6.562 |
| 8 | 1 | 6.830 | .543 | 5.729 | 7.932 |
|  | 2 | 6.290 | .584 | 5.107 | 7.474 |

| **7. power * electrode * conflict** | | | | | | |
| --- | --- | --- | --- | --- | --- | --- |
| Measure: MEASURE_1 | | | | | | |
| power | electrode | conflict | Mean | Std. Error | 95% Confidence Interval | |
|  |  |  |  |  | Lower Bound | Upper Bound |
| 1.00 | 1 | 1 | 1.124 | .887 | -.674 | 2.922 |
|  |  | 2 | .984 | .896 | -.833 | 2.801 |
|  | 2 | 1 | 2.637 | .968 | .673 | 4.601 |
|  |  | 2 | 2.176 | .994 | .160 | 4.192 |
|  | 3 | 1 | 1.799 | .978 | -.183 | 3.782 |
|  |  | 2 | 1.404 | 1.011 | -.646 | 3.454 |
|  | 4 | 1 | 3.001 | .641 | 1.701 | 4.301 |
|  |  | 2 | 2.830 | .605 | 1.603 | 4.056 |
|  | 5 | 1 | 4.924 | .681 | 3.543 | 6.304 |
|  |  | 2 | 4.408 | .707 | 2.975 | 5.842 |
|  | 6 | 1 | 4.377 | .811 | 2.732 | 6.022 |
|  |  | 2 | 3.799 | .837 | 2.102 | 5.496 |
|  | 7 | 1 | 5.664 | .735 | 4.173 | 7.154 |
|  |  | 2 | 5.545 | .748 | 4.029 | 7.062 |
|  | 8 | 1 | 6.929 | .768 | 5.371 | 8.486 |
|  |  | 2 | 6.550 | .825 | 4.876 | 8.224 |
| 2.00 | 1 | 1 | 1.781 | .887 | -.017 | 3.579 |
|  |  | 2 | .893 | .896 | -.924 | 2.710 |
|  | 2 | 1 | 2.060 | .968 | .096 | 4.025 |
|  |  | 2 | 1.543 | .994 | -.473 | 3.559 |
|  | 3 | 1 | 1.578 | .978 | -.405 | 3.560 |
|  |  | 2 | .815 | 1.011 | -1.235 | 2.865 |
|  | 4 | 1 | 3.418 | .641 | 2.119 | 4.718 |
|  |  | 2 | 2.644 | .605 | 1.417 | 3.870 |
|  | 5 | 1 | 4.602 | .681 | 3.221 | 5.982 |
|  |  | 2 | 3.717 | .707 | 2.284 | 5.151 |
|  | 6 | 1 | 4.127 | .811 | 2.482 | 5.772 |
|  |  | 2 | 3.424 | .837 | 1.727 | 5.121 |
|  | 7 | 1 | 6.136 | .735 | 4.645 | 7.626 |
|  |  | 2 | 5.434 | .748 | 3.917 | 6.950 |
|  | 8 | 1 | 6.732 | .768 | 5.175 | 8.290 |
|  |  | 2 | 6.030 | .825 | 4.357 | 7.704 |

**Only fearful faces in P1**

GLM P7 congruent P7incongruent P8 congruent P8incongruent BY power

/WSFACTOR=electrode 2 Polynomial conflict 2 Polynomial

/METHOD=SSTYPE(3)

/POSTHOC=power(BTUKEY LSD BONFERRONI)

/EMMEANS=TABLES(power) COMPARE ADJ(LSD)

/EMMEANS=TABLES(electrode) COMPARE ADJ(LSD)

/EMMEANS=TABLES(conflict) COMPARE ADJ(LSD)

/EMMEANS=TABLES(power*electrode)

/EMMEANS=TABLES(power*conflict)

/EMMEANS=TABLES(electrode*conflict)

/EMMEANS=TABLES(power*electrode*conflict)

/EMMEANS=TABLES(power*conflict) COMPARE(conflict) ADJ(LSD)

/PRINT=DESCRIPTIVE ETASQ OPOWER HOMOGENEITY

/CRITERIA=ALPHA(.05)

/WSDESIGN=electrode conflict electrode*conflict

/DESIGN=power.

**General Linear Model**

| **Notes** | | |
| --- | --- | --- |
| Output Created | | 12-JAN-2021 16:43:06 |
| Comments | |  |
| Input | Data | E:\11.17 \ERP data\p1 fear.sav |
|  | Active Dataset | dataset |
|  | Filter | <none> |
|  | Weight | <none> |
|  | Split File | <none> |
|  | N of Rows in Working Data File | 41 |
| Missing Value Handling | Definition of Missing | User-defined missing values are treated as missing. |
|  | Cases Used | Statistics are based on all cases with valid data for all variables in the model. |
| Syntax | | GLM P7 congruent P7incongruent P8 congruent P8incongruent BY power  /WSFACTOR=electrode 2 Polynomial conflict 2 Polynomial  /METHOD=SSTYPE(3)  /POSTHOC=power(BTUKEY LSD BONFERRONI)  /EMMEANS=TABLES(power) COMPARE ADJ(LSD)  /EMMEANS=TABLES(electrode) COMPARE ADJ(LSD)  /EMMEANS=TABLES(conflict) COMPARE ADJ(LSD)  /EMMEANS=TABLES(power*electrode)  /EMMEANS=TABLES(power*conflict)  /EMMEANS=TABLES(electrode*conflict)  /EMMEANS=TABLES(power*electrode*conflict)  /EMMEANS=TABLES(power*conflict) COMPARE(conflict) ADJ(LSD)  /PRINT=DESCRIPTIVE ETASQ OPOWER HOMOGENEITY  /CRITERIA=ALPHA(.05)  /WSDESIGN=electrode conflict electrode*conflict  /DESIGN=power. |
| Resources | Processor Time | 00:00:00.08 |
|  | Elapsed Time | 00:00:00.09 |

| **Warnings** |
| --- |
| Post hoc tests are not performed for power because there are fewer than three groups. |

| **Within-Subjects Factors** | | |
| --- | --- | --- |
| Measure: MEASURE_1 | | |
| electrode | conflict | Dependent Variable |
| 1 | 1 | P7congruent |
|  | 2 | P7incongruent |
| 2 | 1 | P8congruent |
|  | 2 | P8incongruent |

| **Between-Subjects Factors** | | |
| --- | --- | --- |
|  | | N |
| power | 1.00 | 19 |
|  | 2.00 | 19 |

| **Descriptive Statistics** | | | | |
| --- | --- | --- | --- | --- |
|  | power | Mean | Std. Deviation | N |
| P7congruent | 1.00 | -1.7208 | 3.36565 | 19 |
|  | 2.00 | -.2350 | 2.45046 | 19 |
|  | Total | -.9779 | 2.99979 | 38 |
| P7  incongruent | 1.00 | -1.1557 | 3.36251 | 19 |
|  | 2.00 | -.1556 | 2.37397 | 19 |
|  | Total | -.6557 | 2.91530 | 38 |
| P8congruent | 1.00 | -1.7408 | 4.17533 | 19 |
|  | 2.00 | .3287 | 3.37212 | 19 |
|  | Total | -.7061 | 3.88751 | 38 |
| P8  incongruent | 1.00 | -1.3414 | 4.35361 | 19 |
|  | 2.00 | .3600 | 3.79912 | 19 |
|  | Total | -.4907 | 4.12137 | 38 |

| **Box's Test of Equality of Covariance Matrices^a^** | |
| --- | --- |
| Box's M | 18.065 |
| F | 1.588 |
| df1 | 10 |
| df2 | 6196.016 |
| Sig. | .103 |
| Tests the null hypothesis that the observed covariance matrices of the dependent variables are equal across groups. | |
| a. Design: + power  Within Subjects Design: electrode + conflict + electrode * conflict | |

| **Multivariate Tests^a^** | | | | | | | | | |
| --- | --- | --- | --- | --- | --- | --- | --- | --- | --- |
| Effect | | Value | F | Hypothesis df | Error df | Sig. | Partial Eta Squared | Noncent. Parameter | Observed Power^c^ |
| electrode | Pillai's Trace | .005 | .185^b^ | 1.000 | 36.000 | .670 | .005 | .185 | .070 |
|  | Wilks' Lambda | .995 | .185^b^ | 1.000 | 36.000 | .670 | .005 | .185 | .070 |
|  | Hotelling's Trace | .005 | .185^b^ | 1.000 | 36.000 | .670 | .005 | .185 | .070 |
|  | Roy's Largest Root | .005 | .185^b^ | 1.000 | 36.000 | .670 | .005 | .185 | .070 |
| electrode * power | Pillai's Trace | .011 | .400^b^ | 1.000 | 36.000 | .531 | .011 | .400 | .094 |
|  | Wilks' Lambda | .989 | .400^b^ | 1.000 | 36.000 | .531 | .011 | .400 | .094 |
|  | Hotelling's Trace | .011 | .400^b^ | 1.000 | 36.000 | .531 | .011 | .400 | .094 |
|  | Roy's Largest Root | .011 | .400^b^ | 1.000 | 36.000 | .531 | .011 | .400 | .094 |
| conflict | Pillai's Trace | .154 | 6.557^b^ | 1.000 | 36.000 | .015 | .154 | 6.557 | .703 |
|  | Wilks' Lambda | .846 | 6.557^b^ | 1.000 | 36.000 | .015 | .154 | 6.557 | .703 |
|  | Hotelling's Trace | .182 | 6.557^b^ | 1.000 | 36.000 | .015 | .154 | 6.557 | .703 |
|  | Roy's Largest Root | .182 | 6.557^b^ | 1.000 | 36.000 | .015 | .154 | 6.557 | .703 |
| conflict * power | Pillai's Trace | .103 | 4.134^b^ | 1.000 | 36.000 | .049 | .103 | 4.134 | .508 |
|  | Wilks' Lambda | .897 | 4.134^b^ | 1.000 | 36.000 | .049 | .103 | 4.134 | .508 |
|  | Hotelling's Trace | .115 | 4.134^b^ | 1.000 | 36.000 | .049 | .103 | 4.134 | .508 |
|  | Roy's Largest Root | .115 | 4.134^b^ | 1.000 | 36.000 | .049 | .103 | 4.134 | .508 |
| electrode * conflict | Pillai's Trace | .009 | .312^b^ | 1.000 | 36.000 | .580 | .009 | .312 | .085 |
|  | Wilks' Lambda | .991 | .312^b^ | 1.000 | 36.000 | .580 | .009 | .312 | .085 |
|  | Hotelling's Trace | .009 | .312^b^ | 1.000 | 36.000 | .580 | .009 | .312 | .085 |
|  | Roy's Largest Root | .009 | .312^b^ | 1.000 | 36.000 | .580 | .009 | .312 | .085 |
| electrode * conflict * power | Pillai's Trace | .003 | .094^b^ | 1.000 | 36.000 | .761 | .003 | .094 | .060 |
|  | Wilks' Lambda | .997 | .094^b^ | 1.000 | 36.000 | .761 | .003 | .094 | .060 |
|  | Hotelling's Trace | .003 | .094^b^ | 1.000 | 36.000 | .761 | .003 | .094 | .060 |
|  | Roy's Largest Root | .003 | .094^b^ | 1.000 | 36.000 | .761 | .003 | .094 | .060 |
| a. Design: + power  Within Subjects Design: electrode + conflict + electrode * conflict | | | | | | | | | |
| b. Exact statistic | | | | | | | | | |
| c. Computed using alpha = .05 | | | | | | | | | |

| **Mauchly's Test of Sphericity^a^** | | | | | | | |
| --- | --- | --- | --- | --- | --- | --- | --- |
| Measure: MEASURE_1 | | | | | | | |
| Within Subjects Effect | Mauchly's W | Approx. Chi-Square | df | Sig. | Epsilon^b^ | | |
|  |  |  |  |  | Greenhouse-Geisser | Huynh-Feldt | Lower-bound |
| electrode | 1.000 | .000 | 0 | . | 1.000 | 1.000 | 1.000 |
| conflict | 1.000 | .000 | 0 | . | 1.000 | 1.000 | 1.000 |
| electrode * conflict | 1.000 | .000 | 0 | . | 1.000 | 1.000 | 1.000 |
| Tests the null hypothesis that the error covariance matrix of the orthonormalized transformed dependent variables is proportional to an identity matrix. | | | | | | | |
| a. Design: + power  Within Subjects Design: electrode + conflict + electrode * conflict | | | | | | | |
| b. May be used to adjust the degrees of freedom for the averaged tests of significance. Corrected tests are displayed in the Tests of Within-Subjects Effects table. | | | | | | | |

| **Tests of Within-Subjects Effects** | | | | | | | | | |
| --- | --- | --- | --- | --- | --- | --- | --- | --- | --- |
| Measure: MEASURE_1 | | | | | | | | | |
| Source | | Type III Sum of Squares | df | Mean Square | F | Sig. | Partial Eta Squared | Noncent. Parameter | Observed Power^a^ |
| electrode | Sphericity Assumed | 1.812 | 1 | 1.812 | .185 | .670 | .005 | .185 | .070 |
|  | Greenhouse-Geisser | 1.812 | 1.000 | 1.812 | .185 | .670 | .005 | .185 | .070 |
|  | Huynh-Feldt | 1.812 | 1.000 | 1.812 | .185 | .670 | .005 | .185 | .070 |
|  | Lower-bound | 1.812 | 1.000 | 1.812 | .185 | .670 | .005 | .185 | .070 |
| electrode * power | Sphericity Assumed | 3.922 | 1 | 3.922 | .400 | .531 | .011 | .400 | .094 |
|  | Greenhouse-Geisser | 3.922 | 1.000 | 3.922 | .400 | .531 | .011 | .400 | .094 |
|  | Huynh-Feldt | 3.922 | 1.000 | 3.922 | .400 | .531 | .011 | .400 | .094 |
|  | Lower-bound | 3.922 | 1.000 | 3.922 | .400 | .531 | .011 | .400 | .094 |
| Error(electrode) | Sphericity Assumed | 353.336 | 36 | 9.815 |  |  |  |  |  |
|  | Greenhouse-Geisser | 353.336 | 36.000 | 9.815 |  |  |  |  |  |
|  | Huynh-Feldt | 353.336 | 36.000 | 9.815 |  |  |  |  |  |
|  | Lower-bound | 353.336 | 36.000 | 9.815 |  |  |  |  |  |
| conflict | Sphericity Assumed | 2.745 | 1 | 2.745 | 6.557 | .015 | .154 | 6.557 | .703 |
|  | Greenhouse-Geisser | 2.745 | 1.000 | 2.745 | 6.557 | .015 | .154 | 6.557 | .703 |
|  | Huynh-Feldt | 2.745 | 1.000 | 2.745 | 6.557 | .015 | .154 | 6.557 | .703 |
|  | Lower-bound | 2.745 | 1.000 | 2.745 | 6.557 | .015 | .154 | 6.557 | .703 |
| conflict * power | Sphericity Assumed | 1.731 | 1 | 1.731 | 4.134 | .049 | .103 | 4.134 | .508 |
|  | Greenhouse-Geisser | 1.731 | 1.000 | 1.731 | 4.134 | .049 | .103 | 4.134 | .508 |
|  | Huynh-Feldt | 1.731 | 1.000 | 1.731 | 4.134 | .049 | .103 | 4.134 | .508 |
|  | Lower-bound | 1.731 | 1.000 | 1.731 | 4.134 | .049 | .103 | 4.134 | .508 |
| Error(conflict) | Sphericity Assumed | 15.074 | 36 | .419 |  |  |  |  |  |
|  | Greenhouse-Geisser | 15.074 | 36.000 | .419 |  |  |  |  |  |
|  | Huynh-Feldt | 15.074 | 36.000 | .419 |  |  |  |  |  |
|  | Lower-bound | 15.074 | 36.000 | .419 |  |  |  |  |  |
| electrode * conflict | Sphericity Assumed | .108 | 1 | .108 | .312 | .580 | .009 | .312 | .085 |
|  | Greenhouse-Geisser | .108 | 1.000 | .108 | .312 | .580 | .009 | .312 | .085 |
|  | Huynh-Feldt | .108 | 1.000 | .108 | .312 | .580 | .009 | .312 | .085 |
|  | Lower-bound | .108 | 1.000 | .108 | .312 | .580 | .009 | .312 | .085 |
| electrode * conflict * power | Sphericity Assumed | .033 | 1 | .033 | .094 | .761 | .003 | .094 | .060 |
|  | Greenhouse-Geisser | .033 | 1.000 | .033 | .094 | .761 | .003 | .094 | .060 |
|  | Huynh-Feldt | .033 | 1.000 | .033 | .094 | .761 | .003 | .094 | .060 |
|  | Lower-bound | .033 | 1.000 | .033 | .094 | .761 | .003 | .094 | .060 |
| Error(electrode*conflict) | Sphericity Assumed | 12.518 | 36 | .348 |  |  |  |  |  |
|  | Greenhouse-Geisser | 12.518 | 36.000 | .348 |  |  |  |  |  |
|  | Huynh-Feldt | 12.518 | 36.000 | .348 |  |  |  |  |  |
|  | Lower-bound | 12.518 | 36.000 | .348 |  |  |  |  |  |
| a. Computed using alpha = .05 | | | | | | | | | |

| **Tests of Within-Subjects Contrasts** | | | | | | | | | | |
| --- | --- | --- | --- | --- | --- | --- | --- | --- | --- | --- |
| Measure: MEASURE_1 | | | | | | | | | | |
| Source | electrode | conflict | Type III Sum of Squares | df | Mean Square | F | Sig. | Partial Eta Squared | Noncent. Parameter | Observed Power^a^ |
| electrode | Linear |  | 1.812 | 1 | 1.812 | .185 | .670 | .005 | .185 | .070 |
| electrode * power | Linear |  | 3.922 | 1 | 3.922 | .400 | .531 | .011 | .400 | .094 |
| Error(electrode) | Linear |  | 353.336 | 36 | 9.815 |  |  |  |  |  |
| conflict |  | Linear | 2.745 | 1 | 2.745 | 6.557 | .015 | .154 | 6.557 | .703 |
| conflict * power |  | Linear | 1.731 | 1 | 1.731 | 4.134 | .049 | .103 | 4.134 | .508 |
| Error(conflict) |  | Linear | 15.074 | 36 | .419 |  |  |  |  |  |
| electrode * conflict | Linear | Linear | .108 | 1 | .108 | .312 | .580 | .009 | .312 | .085 |
| electrode * conflict * power | Linear | Linear | .033 | 1 | .033 | .094 | .761 | .003 | .094 | .060 |
| Error(electrode*conflict) | Linear | Linear | 12.518 | 36 | .348 |  |  |  |  |  |
| a. Computed using alpha = .05 | | | | | | | | | | |

| **Levene's Test of Equality of Error Variances^a^** | | | | |
| --- | --- | --- | --- | --- |
|  | F | df1 | df2 | Sig. |
| P7congruent | 3.884 | 1 | 36 | .056 |
| P7incongruent | 3.020 | 1 | 36 | .091 |
| P8congruent | 1.094 | 1 | 36 | .303 |
| P8incongruent | .228 | 1 | 36 | .636 |
| Tests the null hypothesis that the error variance of the dependent variable is equal across groups. | | | | |
| a. Design: + power  Within Subjects Design: electrode + conflict + electrode * conflict | | | | |

| **Tests of Between-Subjects Effects** | | | | | | | | |
| --- | --- | --- | --- | --- | --- | --- | --- | --- |
| Measure: MEASURE_1 | | | | | | | | |
| Transformed Variable: Average | | | | | | | | |
| Source | Type III Sum of Squares | df | Mean Square | F | Sig. | Partial Eta Squared | Noncent. Parameter | Observed Power^a^ |
|  | 76.102 | 1 | 76.102 | 2.021 | .164 | .053 | 2.021 | .283 |
| power | 92.976 | 1 | 92.976 | 2.469 | .125 | .064 | 2.469 | .334 |
| Error | 1355.467 | 36 | 37.652 |  |  |  |  |  |
| a. Computed using alpha = .05 | | | | | | | | |

**Estimated Marginal Means**

**1. power**

| **Estimates** | | | | |
| --- | --- | --- | --- | --- |
| Measure: MEASURE_1 | | | | |
| power | Mean | Std. Error | 95% Confidence Interval | |
|  |  |  | Lower Bound | Upper Bound |
| 1.00 | -1.490 | .704 | -2.917 | -.062 |
| 2.00 | .075 | .704 | -1.353 | 1.502 |

| **Pairwise Comparisons** | | | | | | |
| --- | --- | --- | --- | --- | --- | --- |
| Measure: MEASURE_1 | | | | | | |
| (I) power | (J) power | Mean Difference (I-J) | Std. Error | Sig.^a^ | 95% Confidence Interval for Difference^a^ | |
|  |  |  |  |  | Lower Bound | Upper Bound |
| 1.00 | 2.00 | -1.564 | .995 | .125 | -3.583 | .455 |
| 2.00 | 1.00 | 1.564 | .995 | .125 | -.455 | 3.583 |
| Based on estimated marginal means | | | | | | |
| a. Adjustment for multiple comparisons: Least Significant Difference (equivalent to no adjustments). | | | | | | |

| **Univariate Tests** | | | | | | | | |
| --- | --- | --- | --- | --- | --- | --- | --- | --- |
| Measure: MEASURE_1 | | | | | | | | |
|  | Sum of Squares | df | Mean Square | F | Sig. | Partial Eta Squared | Noncent. Parameter | Observed Power^a^ |
| Contrast | 23.244 | 1 | 23.244 | 2.469 | .125 | .064 | 2.469 | .334 |
| Error | 338.867 | 36 | 9.413 |  |  |  |  |  |
| The F tests the effect of power. This test is based on the linearly independent pairwise comparisons among the estimated marginal means. | | | | | | | | |
| a. Computed using alpha = .05 | | | | | | | | |

**2. electrode**

| **Estimates** | | | | |
| --- | --- | --- | --- | --- |
| Measure: MEASURE_1 | | | | |
| electrode | Mean | Std. Error | 95% Confidence Interval | |
|  |  |  | Lower Bound | Upper Bound |
| 1 | -.817 | .469 | -1.767 | .134 |
| 2 | -.598 | .636 | -1.889 | .692 |

| **Pairwise Comparisons** | | | | | | |
| --- | --- | --- | --- | --- | --- | --- |
| Measure: MEASURE_1 | | | | | | |
| (I) electrode | (J) electrode | Mean Difference (I-J) | Std. Error | Sig.^a^ | 95% Confidence Interval for Difference^a^ | |
|  |  |  |  |  | Lower Bound | Upper Bound |
| 1 | 2 | -.218 | .508 | .670 | -1.249 | .812 |
| 2 | 1 | .218 | .508 | .670 | -.812 | 1.249 |
| Based on estimated marginal means | | | | | | |
| a. Adjustment for multiple comparisons: Least Significant Difference (equivalent to no adjustments). | | | | | | |

| **Multivariate Tests** | | | | | | | | |
| --- | --- | --- | --- | --- | --- | --- | --- | --- |
|  | Value | F | Hypothesis df | Error df | Sig. | Partial Eta Squared | Noncent. Parameter | Observed Power^b^ |
| Pillai's trace | .005 | .185^a^ | 1.000 | 36.000 | .670 | .005 | .185 | .070 |
| Wilks' lambda | .995 | .185^a^ | 1.000 | 36.000 | .670 | .005 | .185 | .070 |
| Hotelling's trace | .005 | .185^a^ | 1.000 | 36.000 | .670 | .005 | .185 | .070 |
| Roy's largest root | .005 | .185^a^ | 1.000 | 36.000 | .670 | .005 | .185 | .070 |
| Each F tests the multivariate effect of electrode. These tests are based on the linearly independent pairwise comparisons among the estimated marginal means. | | | | | | | | |
| a. Exact statistic | | | | | | | | |
| b. Computed using alpha = .05 | | | | | | | | |

**3. conflict**

| **Estimates** | | | | |
| --- | --- | --- | --- | --- |
| Measure: MEASURE_1 | | | | |
| conflict | Mean | Std. Error | 95% Confidence Interval | |
|  |  |  | Lower Bound | Upper Bound |
| 1 | -.842 | .489 | -1.834 | .150 |
| 2 | -.573 | .512 | -1.611 | .465 |

| **Pairwise Comparisons** | | | | | | |
| --- | --- | --- | --- | --- | --- | --- |
| Measure: MEASURE_1 | | | | | | |
| (I) conflict | (J) conflict | Mean Difference (I-J) | Std. Error | Sig.^b^ | 95% Confidence Interval for Difference^b^ | |
|  |  |  |  |  | Lower Bound | Upper Bound |
| 1 | 2 | -.269^*^ | .105 | .015 | -.482 | -.056 |
| 2 | 1 | .269^*^ | .105 | .015 | .056 | .482 |
| Based on estimated marginal means | | | | | | |
| *. The mean difference is significant at the .05 level. | | | | | | |
| b. Adjustment for multiple comparisons: Least Significant Difference (equivalent to no adjustments). | | | | | | |

| **Multivariate Tests** | | | | | | | | |
| --- | --- | --- | --- | --- | --- | --- | --- | --- |
|  | Value | F | Hypothesis df | Error df | Sig. | Partial Eta Squared | Noncent. Parameter | Observed Power^b^ |
| Pillai's trace | .154 | 6.557^a^ | 1.000 | 36.000 | .015 | .154 | 6.557 | .703 |
| Wilks' lambda | .846 | 6.557^a^ | 1.000 | 36.000 | .015 | .154 | 6.557 | .703 |
| Hotelling's trace | .182 | 6.557^a^ | 1.000 | 36.000 | .015 | .154 | 6.557 | .703 |
| Roy's largest root | .182 | 6.557^a^ | 1.000 | 36.000 | .015 | .154 | 6.557 | .703 |
| Each F tests the multivariate effect of conflict. These tests are based on the linearly independent pairwise comparisons among the estimated marginal means. | | | | | | | | |
| a. Exact statistic | | | | | | | | |
| b. Computed using alpha = .05 | | | | | | | | |

| **4. power * electrode** | | | | | |
| --- | --- | --- | --- | --- | --- |
| Measure: MEASURE_1 | | | | | |
| power | electrode | Mean | Std. Error | 95% Confidence Interval | |
|  |  |  |  | Lower Bound | Upper Bound |
| 1.00 | 1 | -1.438 | .663 | -2.782 | -.094 |
|  | 2 | -1.541 | .900 | -3.366 | .284 |
| 2.00 | 1 | -.195 | .663 | -1.540 | 1.149 |
|  | 2 | .344 | .900 | -1.481 | 2.169 |

| **5. power * conflict** | | | | | |
| --- | --- | --- | --- | --- | --- |
| Measure: MEASURE_1 | | | | | |
| power | conflict | Mean | Std. Error | 95% Confidence Interval | |
|  |  |  |  | Lower Bound | Upper Bound |
| 1.00 | 1 | -1.731 | .692 | -3.133 | -.328 |
|  | 2 | -1.249 | .724 | -2.716 | .219 |
| 2.00 | 1 | .047 | .692 | -1.356 | 1.449 |
|  | 2 | .102 | .724 | -1.365 | 1.570 |

| **6. electrode * conflict** | | | | | |
| --- | --- | --- | --- | --- | --- |
| Measure: MEASURE_1 | | | | | |
| electrode | conflict | Mean | Std. Error | 95% Confidence Interval | |
|  |  |  |  | Lower Bound | Upper Bound |
| 1 | 1 | -.978 | .478 | -1.946 | -.009 |
|  | 2 | -.656 | .472 | -1.613 | .302 |
| 2 | 1 | -.706 | .616 | -1.955 | .542 |
|  | 2 | -.491 | .663 | -1.835 | .854 |

| **7. power * electrode * conflict** | | | | | | |
| --- | --- | --- | --- | --- | --- | --- |
| Measure: MEASURE_1 | | | | | | |
| power | electrode | conflict | Mean | Std. Error | 95% Confidence Interval | |
|  |  |  |  |  | Lower Bound | Upper Bound |
| 1.00 | 1 | 1 | -1.721 | .675 | -3.090 | -.351 |
|  |  | 2 | -1.156 | .668 | -2.510 | .198 |
|  | 2 | 1 | -1.741 | .871 | -3.507 | .025 |
|  |  | 2 | -1.341 | .937 | -3.242 | .560 |
| 2.00 | 1 | 1 | -.235 | .675 | -1.605 | 1.135 |
|  |  | 2 | -.156 | .668 | -1.510 | 1.199 |
|  | 2 | 1 | .329 | .871 | -1.437 | 2.094 |
|  |  | 2 | .360 | .937 | -1.541 | 2.261 |

**8. power * conflict**

| **Estimates** | | | | | |
| --- | --- | --- | --- | --- | --- |
| Measure: MEASURE_1 | | | | | |
| power | conflict | Mean | Std. Error | 95% Confidence Interval | |
|  |  |  |  | Lower Bound | Upper Bound |
| 1.00 | 1 | -1.731 | .692 | -3.133 | -.328 |
|  | 2 | -1.249 | .724 | -2.716 | .219 |
| 2.00 | 1 | .047 | .692 | -1.356 | 1.449 |
|  | 2 | .102 | .724 | -1.365 | 1.570 |

| **Pairwise Comparisons** | | | | | | | |
| --- | --- | --- | --- | --- | --- | --- | --- |
| Measure: MEASURE_1 | | | | | | | |
| power | (I) conflict | (J) conflict | Mean Difference (I-J) | Std. Error | Sig.^b^ | 95% Confidence Interval for Difference^b^ | |
|  |  |  |  |  |  | Lower Bound | Upper Bound |
| 1.00 | 1 | 2 | -.482^*^ | .148 | .003 | -.783 | -.181 |
|  | 2 | 1 | .482^*^ | .148 | .003 | .181 | .783 |
| 2.00 | 1 | 2 | -.055 | .148 | .711 | -.356 | .246 |
|  | 2 | 1 | .055 | .148 | .711 | -.246 | .356 |
| Based on estimated marginal means | | | | | | | |
| *. The mean difference is significant at the .05 level. | | | | | | | |
| b. Adjustment for multiple comparisons: Least Significant Difference (equivalent to no adjustments). | | | | | | | |

| **Multivariate Tests** | | | | | | | | | |
| --- | --- | --- | --- | --- | --- | --- | --- | --- | --- |
| power | | Value | F | Hypothesis df | Error df | Sig. | Partial Eta Squared | Noncent. Parameter | Observed Power^b^ |
| 1.00 | Pillai's trace | .227 | 10.552^a^ | 1.000 | 36.000 | .003 | .227 | 10.552 | .885 |
|  | Wilks' lambda | .773 | 10.552^a^ | 1.000 | 36.000 | .003 | .227 | 10.552 | .885 |
|  | Hotelling's trace | .293 | 10.552^a^ | 1.000 | 36.000 | .003 | .227 | 10.552 | .885 |
|  | Roy's largest root | .293 | 10.552^a^ | 1.000 | 36.000 | .003 | .227 | 10.552 | .885 |
| 2.00 | Pillai's trace | .004 | .139^a^ | 1.000 | 36.000 | .711 | .004 | .139 | .065 |
|  | Wilks' lambda | .996 | .139^a^ | 1.000 | 36.000 | .711 | .004 | .139 | .065 |
|  | Hotelling's trace | .004 | .139^a^ | 1.000 | 36.000 | .711 | .004 | .139 | .065 |
|  | Roy's largest root | .004 | .139^a^ | 1.000 | 36.000 | .711 | .004 | .139 | .065 |
| Each F tests the multivariate simple effects of conflict within each level combination of the other effects shown. These tests are based on the linearly independent pairwise comparisons among the estimated marginal means. | | | | | | | | | |
| a. Exact statistic | | | | | | | | | |
| b. Computed using alpha = .05 | | | | | | | | | |

**Only fearful faces in N170**

GLM P7 congruent P7incongruent P8 congruent P8incongruent BY power

/WSFACTOR=electrode 2 Polynomial conflict 2 Polynomial

/METHOD=SSTYPE(3)

/EMMEANS=TABLES(power) COMPARE ADJ(LSD)

/EMMEANS=TABLES(electrode) COMPARE ADJ(LSD)

/EMMEANS=TABLES(conflict) COMPARE ADJ(LSD)

/EMMEANS=TABLES(power*electrode)

/EMMEANS=TABLES(power*conflict)

/EMMEANS=TABLES(electrode*conflict)

/EMMEANS=TABLES(power*electrode*conflict)

/PRINT=DESCRIPTIVE ETASQ OPOWER HOMOGENEITY

/CRITERIA=ALPHA(.05)

/WSDESIGN=electrode conflict electrode*conflict

/DESIGN=power.

**General Linear Model**

| **Notes** | | |
| --- | --- | --- |
| Output Created | | 04-DEC-2020 22:43:06 |
| Comments | |  |
| Input | Data | C:\Users\lenovo\Desktop\N170 fear.sav |
|  | Active Dataset | dataset |
|  | Filter | <none> |
|  | Weight | <none> |
|  | Split File | <none> |
|  | N of Rows in Working Data File | 48 |
| Missing Value Handling | Definition of Missing | User-defined missing values are treated as missing. |
|  | Cases Used | Statistics are based on all cases with valid data for all variables in the model. |
| Syntax | | GLM P7 congruent P7incongruent P8 congruent P8incongruent BY power  /WSFACTOR=electrode 2 Polynomial conflict 2 Polynomial  /METHOD=SSTYPE(3)  /EMMEANS=TABLES(power) COMPARE ADJ(LSD)  /EMMEANS=TABLES(electrode) COMPARE ADJ(LSD)  /EMMEANS=TABLES(conflict) COMPARE ADJ(LSD)  /EMMEANS=TABLES(power*electrode)  /EMMEANS=TABLES(power*conflict)  /EMMEANS=TABLES(electrode*conflict)  /EMMEANS=TABLES(power*electrode*conflict)  /PRINT=DESCRIPTIVE ETASQ OPOWER HOMOGENEITY  /CRITERIA=ALPHA(.05)  /WSDESIGN=electrode conflict electrode*conflict  /DESIGN=power. |
| Resources | Processor Time | 00:00:00.05 |
|  | Elapsed Time | 00:00:00.03 |

| **Within-Subjects Factors** | | |
| --- | --- | --- |
| Measure: MEASURE_1 | | |
| electrode | conflict | Dependent Variable |
| 1 | 1 | P7congruent |
|  | 2 | P7incongruent |
| 2 | 1 | P8congruent |
|  | 2 | P8incongruent |

| **Between-Subjects Factors** | | |
| --- | --- | --- |
|  | | N |
| power | 1.00 | 19 |
|  | 2.00 | 19 |

| **Descriptive Statistics** | | | | |
| --- | --- | --- | --- | --- |
|  | power | Mean | Std. Deviation | N |
| P7congruent | 1.00 | -5.1061 | 4.86534 | 19 |
|  | 2.00 | -2.5989 | 2.68610 | 19 |
|  | Total | -3.8525 | 4.07920 | 38 |
| P7  incongruent | 1.00 | -4.5865 | 4.92502 | 19 |
|  | 2.00 | -2.5027 | 2.71880 | 19 |
|  | Total | -3.5446 | 4.06339 | 38 |
| P8congruent | 1.00 | -6.2349 | 6.54714 | 19 |
|  | 2.00 | -3.1414 | 3.98577 | 19 |
|  | Total | -4.6881 | 5.57125 | 38 |
| P8  incongruent | 1.00 | -5.8716 | 6.78421 | 19 |
|  | 2.00 | -3.0085 | 4.32480 | 19 |
|  | Total | -4.4400 | 5.79610 | 38 |

| **Box's Test of Equality of Covariance Matrices^a^** | |
| --- | --- |
| Box's M | 23.880 |
| F | 2.099 |
| df1 | 10 |
| df2 | 6196.016 |
| Sig. | .021 |
| Tests the null hypothesis that the observed covariance matrices of the dependent variables are equal across groups. | |
| a. Design: + power  Within Subjects Design: electrode + conflict + electrode * conflict | |

| **Multivariate Tests^a^** | | | | | | | | | |
| --- | --- | --- | --- | --- | --- | --- | --- | --- | --- |
| Effect | | Value | F | Hypothesis df | Error df | Sig. | Partial Eta Squared | Noncent. Parameter | Observed Power^c^ |
| electrode | Pillai's Trace | .053 | 2.024^b^ | 1.000 | 36.000 | .163 | .053 | 2.024 | .283 |
|  | Wilks' Lambda | .947 | 2.024^b^ | 1.000 | 36.000 | .163 | .053 | 2.024 | .283 |
|  | Hotelling's Trace | .056 | 2.024^b^ | 1.000 | 36.000 | .163 | .053 | 2.024 | .283 |
|  | Roy's Largest Root | .056 | 2.024^b^ | 1.000 | 36.000 | .163 | .053 | 2.024 | .283 |
| electrode * power | Pillai's Trace | .009 | .315^b^ | 1.000 | 36.000 | .578 | .009 | .315 | .085 |
|  | Wilks' Lambda | .991 | .315^b^ | 1.000 | 36.000 | .578 | .009 | .315 | .085 |
|  | Hotelling's Trace | .009 | .315^b^ | 1.000 | 36.000 | .578 | .009 | .315 | .085 |
|  | Roy's Largest Root | .009 | .315^b^ | 1.000 | 36.000 | .578 | .009 | .315 | .085 |
| conflict | Pillai's Trace | .104 | 4.187^b^ | 1.000 | 36.000 | .048 | .104 | 4.187 | .513 |
|  | Wilks' Lambda | .896 | 4.187^b^ | 1.000 | 36.000 | .048 | .104 | 4.187 | .513 |
|  | Hotelling's Trace | .116 | 4.187^b^ | 1.000 | 36.000 | .048 | .104 | 4.187 | .513 |
|  | Roy's Largest Root | .116 | 4.187^b^ | 1.000 | 36.000 | .048 | .104 | 4.187 | .513 |
| conflict * power | Pillai's Trace | .039 | 1.447^b^ | 1.000 | 36.000 | .237 | .039 | 1.447 | .216 |
|  | Wilks' Lambda | .961 | 1.447^b^ | 1.000 | 36.000 | .237 | .039 | 1.447 | .216 |
|  | Hotelling's Trace | .040 | 1.447^b^ | 1.000 | 36.000 | .237 | .039 | 1.447 | .216 |
|  | Roy's Largest Root | .040 | 1.447^b^ | 1.000 | 36.000 | .237 | .039 | 1.447 | .216 |
| electrode * conflict | Pillai's Trace | .002 | .088^b^ | 1.000 | 36.000 | .768 | .002 | .088 | .060 |
|  | Wilks' Lambda | .998 | .088^b^ | 1.000 | 36.000 | .768 | .002 | .088 | .060 |
|  | Hotelling's Trace | .002 | .088^b^ | 1.000 | 36.000 | .768 | .002 | .088 | .060 |
|  | Roy's Largest Root | .002 | .088^b^ | 1.000 | 36.000 | .768 | .002 | .088 | .060 |
| electrode * conflict * power | Pillai's Trace | .006 | .229^b^ | 1.000 | 36.000 | .635 | .006 | .229 | .075 |
|  | Wilks' Lambda | .994 | .229^b^ | 1.000 | 36.000 | .635 | .006 | .229 | .075 |
|  | Hotelling's Trace | .006 | .229^b^ | 1.000 | 36.000 | .635 | .006 | .229 | .075 |
|  | Roy's Largest Root | .006 | .229^b^ | 1.000 | 36.000 | .635 | .006 | .229 | .075 |
| a. Design: + power  Within Subjects Design: electrode + conflict + electrode * conflict | | | | | | | | | |
| b. Exact statistic | | | | | | | | | |
| c. Computed using alpha = .05 | | | | | | | | | |

| **Mauchly's Test of Sphericity^a^** | | | | | | | |
| --- | --- | --- | --- | --- | --- | --- | --- |
| Measure: MEASURE_1 | | | | | | | |
| Within Subjects Effect | Mauchly's W | Approx. Chi-Square | df | Sig. | Epsilon^b^ | | |
|  |  |  |  |  | Greenhouse-Geisser | Huynh-Feldt | Lower-bound |
| electrode | 1.000 | .000 | 0 | . | 1.000 | 1.000 | 1.000 |
| conflict | 1.000 | .000 | 0 | . | 1.000 | 1.000 | 1.000 |
| electrode * conflict | 1.000 | .000 | 0 | . | 1.000 | 1.000 | 1.000 |
| Tests the null hypothesis that the error covariance matrix of the orthonormalized transformed dependent variables is proportional to an identity matrix. | | | | | | | |
| a. Design: + power  Within Subjects Design: electrode + conflict + electrode * conflict | | | | | | | |
| b. May be used to adjust the degrees of freedom for the averaged tests of significance. Corrected tests are displayed in the Tests of Within-Subjects Effects table. | | | | | | | |

| **Tests of Within-Subjects Effects** | | | | | | | | | |
| --- | --- | --- | --- | --- | --- | --- | --- | --- | --- |
| Measure: MEASURE_1 | | | | | | | | | |
| Source | | Type III Sum of Squares | df | Mean Square | F | Sig. | Partial Eta Squared | Noncent. Parameter | Observed Power^a^ |
| electrode | Sphericity Assumed | 28.468 | 1 | 28.468 | 2.024 | .163 | .053 | 2.024 | .283 |
|  | Greenhouse-Geisser | 28.468 | 1.000 | 28.468 | 2.024 | .163 | .053 | 2.024 | .283 |
|  | Huynh-Feldt | 28.468 | 1.000 | 28.468 | 2.024 | .163 | .053 | 2.024 | .283 |
|  | Lower-bound | 28.468 | 1.000 | 28.468 | 2.024 | .163 | .053 | 2.024 | .283 |
| electrode * power | Sphericity Assumed | 4.429 | 1 | 4.429 | .315 | .578 | .009 | .315 | .085 |
|  | Greenhouse-Geisser | 4.429 | 1.000 | 4.429 | .315 | .578 | .009 | .315 | .085 |
|  | Huynh-Feldt | 4.429 | 1.000 | 4.429 | .315 | .578 | .009 | .315 | .085 |
|  | Lower-bound | 4.429 | 1.000 | 4.429 | .315 | .578 | .009 | .315 | .085 |
| Error(electrode) | Sphericity Assumed | 506.419 | 36 | 14.067 |  |  |  |  |  |
|  | Greenhouse-Geisser | 506.419 | 36.000 | 14.067 |  |  |  |  |  |
|  | Huynh-Feldt | 506.419 | 36.000 | 14.067 |  |  |  |  |  |
|  | Lower-bound | 506.419 | 36.000 | 14.067 |  |  |  |  |  |
| conflict | Sphericity Assumed | 2.936 | 1 | 2.936 | 4.187 | .048 | .104 | 4.187 | .513 |
|  | Greenhouse-Geisser | 2.936 | 1.000 | 2.936 | 4.187 | .048 | .104 | 4.187 | .513 |
|  | Huynh-Feldt | 2.936 | 1.000 | 2.936 | 4.187 | .048 | .104 | 4.187 | .513 |
|  | Lower-bound | 2.936 | 1.000 | 2.936 | 4.187 | .048 | .104 | 4.187 | .513 |
| conflict * power | Sphericity Assumed | 1.015 | 1 | 1.015 | 1.447 | .237 | .039 | 1.447 | .216 |
|  | Greenhouse-Geisser | 1.015 | 1.000 | 1.015 | 1.447 | .237 | .039 | 1.447 | .216 |
|  | Huynh-Feldt | 1.015 | 1.000 | 1.015 | 1.447 | .237 | .039 | 1.447 | .216 |
|  | Lower-bound | 1.015 | 1.000 | 1.015 | 1.447 | .237 | .039 | 1.447 | .216 |
| Error(conflict) | Sphericity Assumed | 25.246 | 36 | .701 |  |  |  |  |  |
|  | Greenhouse-Geisser | 25.246 | 36.000 | .701 |  |  |  |  |  |
|  | Huynh-Feldt | 25.246 | 36.000 | .701 |  |  |  |  |  |
|  | Lower-bound | 25.246 | 36.000 | .701 |  |  |  |  |  |
| electrode * conflict | Sphericity Assumed | .034 | 1 | .034 | .088 | .768 | .002 | .088 | .060 |
|  | Greenhouse-Geisser | .034 | 1.000 | .034 | .088 | .768 | .002 | .088 | .060 |
|  | Huynh-Feldt | .034 | 1.000 | .034 | .088 | .768 | .002 | .088 | .060 |
|  | Lower-bound | .034 | 1.000 | .034 | .088 | .768 | .002 | .088 | .060 |
| electrode * conflict * power | Sphericity Assumed | .088 | 1 | .088 | .229 | .635 | .006 | .229 | .075 |
|  | Greenhouse-Geisser | .088 | 1.000 | .088 | .229 | .635 | .006 | .229 | .075 |
|  | Huynh-Feldt | .088 | 1.000 | .088 | .229 | .635 | .006 | .229 | .075 |
|  | Lower-bound | .088 | 1.000 | .088 | .229 | .635 | .006 | .229 | .075 |
| Error(electrode*conflict) | Sphericity Assumed | 13.878 | 36 | .385 |  |  |  |  |  |
|  | Greenhouse-Geisser | 13.878 | 36.000 | .385 |  |  |  |  |  |
|  | Huynh-Feldt | 13.878 | 36.000 | .385 |  |  |  |  |  |
|  | Lower-bound | 13.878 | 36.000 | .385 |  |  |  |  |  |
| a. Computed using alpha = .05 | | | | | | | | | |

| **Tests of Within-Subjects Contrasts** | | | | | | | | | | |
| --- | --- | --- | --- | --- | --- | --- | --- | --- | --- | --- |
| Measure: MEASURE_1 | | | | | | | | | | |
| Source | electrode | conflict | Type III Sum of Squares | df | Mean Square | F | Sig. | Partial Eta Squared | Noncent. Parameter | Observed Power^a^ |
| electrode | Linear |  | 28.468 | 1 | 28.468 | 2.024 | .163 | .053 | 2.024 | .283 |
| electrode * power | Linear |  | 4.429 | 1 | 4.429 | .315 | .578 | .009 | .315 | .085 |
| Error(electrode) | Linear |  | 506.419 | 36 | 14.067 |  |  |  |  |  |
| conflict |  | Linear | 2.936 | 1 | 2.936 | 4.187 | .048 | .104 | 4.187 | .513 |
| conflict * power |  | Linear | 1.015 | 1 | 1.015 | 1.447 | .237 | .039 | 1.447 | .216 |
| Error(conflict) |  | Linear | 25.246 | 36 | .701 |  |  |  |  |  |
| electrode * conflict | Linear | Linear | .034 | 1 | .034 | .088 | .768 | .002 | .088 | .060 |
| electrode * conflict * power | Linear | Linear | .088 | 1 | .088 | .229 | .635 | .006 | .229 | .075 |
| Error(electrode*conflict) | Linear | Linear | 13.878 | 36 | .385 |  |  |  |  |  |
| a. Computed using alpha = .05 | | | | | | | | | | |

| **Levene's Test of Equality of Error Variances^a^** | | | | |
| --- | --- | --- | --- | --- |
|  | F | df1 | df2 | Sig. |
| P7congruent | 7.453 | 1 | 36 | .010 |
| P7incongruent | 6.454 | 1 | 36 | .016 |
| P8congruent | 4.209 | 1 | 36 | .048 |
| P8incongruent | 3.325 | 1 | 36 | .077 |
| Tests the null hypothesis that the error variance of the dependent variable is equal across groups. | | | | |
| a. Design: + power  Within Subjects Design: electrode + conflict + electrode * conflict | | | | |

| **Tests of Between-Subjects Effects** | | | | | | | | |
| --- | --- | --- | --- | --- | --- | --- | --- | --- |
| Measure: MEASURE_1 | | | | | | | | |
| Transformed Variable: Average | | | | | | | | |
| Source | Type III Sum of Squares | df | Mean Square | F | Sig. | Partial Eta Squared | Noncent. Parameter | Observed Power^a^ |
|  | 2594.295 | 1 | 2594.295 | 33.323 | .000 | .481 | 33.323 | 1.000 |
| power | 264.226 | 1 | 264.226 | 3.394 | .074 | .086 | 3.394 | .434 |
| Error | 2802.729 | 36 | 77.854 |  |  |  |  |  |
| a. Computed using alpha = .05 | | | | | | | | |

**Estimated Marginal Means**

**1. power**

| **Estimates** | | | | |
| --- | --- | --- | --- | --- |
| Measure: MEASURE_1 | | | | |
| power | Mean | Std. Error | 95% Confidence Interval | |
|  |  |  | Lower Bound | Upper Bound |
| 1.00 | -5.450 | 1.012 | -7.502 | -3.397 |
| 2.00 | -2.813 | 1.012 | -4.866 | -.760 |

| **Pairwise Comparisons** | | | | | | |
| --- | --- | --- | --- | --- | --- | --- |
| Measure: MEASURE_1 | | | | | | |
| (I) power | (J) power | Mean Difference (I-J) | Std. Error | Sig.^a^ | 95% Confidence Interval for Difference^a^ | |
|  |  |  |  |  | Lower Bound | Upper Bound |
| 1.00 | 2.00 | -2.637 | 1.431 | .074 | -5.540 | .266 |
| 2.00 | 1.00 | 2.637 | 1.431 | .074 | -.266 | 5.540 |
| Based on estimated marginal means | | | | | | |
| a. Adjustment for multiple comparisons: Least Significant Difference (equivalent to no adjustments). | | | | | | |

| **Univariate Tests** | | | | | | | | |
| --- | --- | --- | --- | --- | --- | --- | --- | --- |
| Measure: MEASURE_1 | | | | | | | | |
|  | Sum of Squares | df | Mean Square | F | Sig. | Partial Eta Squared | Noncent. Parameter | Observed Power^a^ |
| Contrast | 66.056 | 1 | 66.056 | 3.394 | .074 | .086 | 3.394 | .434 |
| Error | 700.682 | 36 | 19.463 |  |  |  |  |  |
| The F tests the effect of power. This test is based on the linearly independent pairwise comparisons among the estimated marginal means. | | | | | | | | |
| a. Computed using alpha = .05 | | | | | | | | |

**2. electrode**

| **Estimates** | | | | |
| --- | --- | --- | --- | --- |
| Measure: MEASURE_1 | | | | |
| electrode | Mean | Std. Error | 95% Confidence Interval | |
|  |  |  | Lower Bound | Upper Bound |
| 1 | -3.699 | .635 | -4.986 | -2.411 |
| 2 | -4.564 | .898 | -6.385 | -2.743 |

| **Pairwise Comparisons** | | | | | | |
| --- | --- | --- | --- | --- | --- | --- |
| Measure: MEASURE_1 | | | | | | |
| (I) electrode | (J) electrode | Mean Difference (I-J) | Std. Error | Sig.^a^ | 95% Confidence Interval for Difference^a^ | |
|  |  |  |  |  | Lower Bound | Upper Bound |
| 1 | 2 | .866 | .608 | .163 | -.368 | 2.099 |
| 2 | 1 | -.866 | .608 | .163 | -2.099 | .368 |
| Based on estimated marginal means | | | | | | |
| a. Adjustment for multiple comparisons: Least Significant Difference (equivalent to no adjustments). | | | | | | |

| **Multivariate Tests** | | | | | | | | |
| --- | --- | --- | --- | --- | --- | --- | --- | --- |
|  | Value | F | Hypothesis df | Error df | Sig. | Partial Eta Squared | Noncent. Parameter | Observed Power^b^ |
| Pillai's trace | .053 | 2.024^a^ | 1.000 | 36.000 | .163 | .053 | 2.024 | .283 |
| Wilks' lambda | .947 | 2.024^a^ | 1.000 | 36.000 | .163 | .053 | 2.024 | .283 |
| Hotelling's trace | .056 | 2.024^a^ | 1.000 | 36.000 | .163 | .053 | 2.024 | .283 |
| Roy's largest root | .056 | 2.024^a^ | 1.000 | 36.000 | .163 | .053 | 2.024 | .283 |
| Each F tests the multivariate effect of electrode. These tests are based on the linearly independent pairwise comparisons among the estimated marginal means. | | | | | | | | |
| a. Exact statistic | | | | | | | | |
| b. Computed using alpha = .05 | | | | | | | | |

**3. conflict**

| **Estimates** | | | | |
| --- | --- | --- | --- | --- |
| Measure: MEASURE_1 | | | | |
| conflict | Mean | Std. Error | 95% Confidence Interval | |
|  |  |  | Lower Bound | Upper Bound |
| 1 | -4.270 | .705 | -5.700 | -2.841 |
| 2 | -3.992 | .733 | -5.478 | -2.507 |

| **Pairwise Comparisons** | | | | | | |
| --- | --- | --- | --- | --- | --- | --- |
| Measure: MEASURE_1 | | | | | | |
| (I) conflict | (J) conflict | Mean Difference (I-J) | Std. Error | Sig.^b^ | 95% Confidence Interval for Difference^b^ | |
|  |  |  |  |  | Lower Bound | Upper Bound |
| 1 | 2 | -.278^*^ | .136 | .048 | -.553 | -.002 |
| 2 | 1 | .278^*^ | .136 | .048 | .002 | .553 |
| Based on estimated marginal means | | | | | | |
| *. The mean difference is significant at the .05 level. | | | | | | |
| b. Adjustment for multiple comparisons: Least Significant Difference (equivalent to no adjustments). | | | | | | |

| **Multivariate Tests** | | | | | | | | |
| --- | --- | --- | --- | --- | --- | --- | --- | --- |
|  | Value | F | Hypothesis df | Error df | Sig. | Partial Eta Squared | Noncent. Parameter | Observed Power^b^ |
| Pillai's trace | .104 | 4.187^a^ | 1.000 | 36.000 | .048 | .104 | 4.187 | .513 |
| Wilks' lambda | .896 | 4.187^a^ | 1.000 | 36.000 | .048 | .104 | 4.187 | .513 |
| Hotelling's trace | .116 | 4.187^a^ | 1.000 | 36.000 | .048 | .104 | 4.187 | .513 |
| Roy's largest root | .116 | 4.187^a^ | 1.000 | 36.000 | .048 | .104 | 4.187 | .513 |
| Each F tests the multivariate effect of conflict. These tests are based on the linearly independent pairwise comparisons among the estimated marginal means. | | | | | | | | |
| a. Exact statistic | | | | | | | | |
| b. Computed using alpha = .05 | | | | | | | | |

| **4. power * electrode** | | | | | |
| --- | --- | --- | --- | --- | --- |
| Measure: MEASURE_1 | | | | | |
| power | electrode | Mean | Std. Error | 95% Confidence Interval | |
|  |  |  |  | Lower Bound | Upper Bound |
| 1.00 | 1 | -4.846 | .898 | -6.667 | -3.025 |
|  | 2 | -6.053 | 1.270 | -8.629 | -3.478 |
| 2.00 | 1 | -2.551 | .898 | -4.372 | -.730 |
|  | 2 | -3.075 | 1.270 | -5.650 | -.499 |

| **5. power * conflict** | | | | | |
| --- | --- | --- | --- | --- | --- |
| Measure: MEASURE_1 | | | | | |
| power | conflict | Mean | Std. Error | 95% Confidence Interval | |
|  |  |  |  | Lower Bound | Upper Bound |
| 1.00 | 1 | -5.670 | .997 | -7.692 | -3.649 |
|  | 2 | -5.229 | 1.036 | -7.330 | -3.128 |
| 2.00 | 1 | -2.870 | .997 | -4.892 | -.848 |
|  | 2 | -2.756 | 1.036 | -4.857 | -.654 |

| **6. electrode * conflict** | | | | | |
| --- | --- | --- | --- | --- | --- |
| Measure: MEASURE_1 | | | | | |
| electrode | conflict | Mean | Std. Error | 95% Confidence Interval | |
|  |  |  |  | Lower Bound | Upper Bound |
| 1 | 1 | -3.852 | .637 | -5.145 | -2.560 |
|  | 2 | -3.545 | .645 | -4.853 | -2.236 |
| 2 | 1 | -4.688 | .879 | -6.471 | -2.905 |
|  | 2 | -4.440 | .923 | -6.312 | -2.568 |

| **7. power * electrode * conflict** | | | | | | |
| --- | --- | --- | --- | --- | --- | --- |
| Measure: MEASURE_1 | | | | | | |
| power | electrode | conflict | Mean | Std. Error | 95% Confidence Interval | |
|  |  |  |  |  | Lower Bound | Upper Bound |
| 1.00 | 1 | 1 | -5.106 | .902 | -6.934 | -3.278 |
|  |  | 2 | -4.587 | .913 | -6.437 | -2.736 |
|  | 2 | 1 | -6.235 | 1.243 | -8.757 | -3.713 |
|  |  | 2 | -5.872 | 1.305 | -8.519 | -3.225 |
| 2.00 | 1 | 1 | -2.599 | .902 | -4.427 | -.770 |
|  |  | 2 | -2.503 | .913 | -4.354 | -.652 |
|  | 2 | 1 | -3.141 | 1.243 | -5.663 | -.620 |
|  |  | 2 | -3.008 | 1.305 | -5.655 | -.362 |

**Only fearful faces in N450**

GLM F3 congruent F3incongruent F4 congruent F4incongruent FZ congruent FZ incongruent C3 congruent C3incongruent C4 congruent C4incongruent CZ congruent CZ incongruent CP1 congruent CP1incongruent CP2 congruent CP2incongruent BY power

/WSFACTOR=electrode 8 Polynomial conflict 2 Polynomial

/METHOD=SSTYPE(3)

/EMMEANS=TABLES(power) COMPARE ADJ(LSD)

/EMMEANS=TABLES(electrode) COMPARE ADJ(LSD)

/EMMEANS=TABLES(conflict) COMPARE ADJ(LSD)

/EMMEANS=TABLES(power*electrode)

/EMMEANS=TABLES(power*conflict)

/EMMEANS=TABLES(electrode*conflict)

/EMMEANS=TABLES(power*electrode*conflict)

/PRINT=DESCRIPTIVE ETASQ OPOWER HOMOGENEITY

/CRITERIA=ALPHA(.05)

/WSDESIGN=electrode conflict electrode*conflict

/DESIGN=power.

**General Linear Model**

| **Notes** | | |
| --- | --- | --- |
| Output Created | | 04-DEC-2020 23:25:45 |
| Comments | |  |
| Input | Data | C:\Users\lenovo\Desktop\N450 fear.sav |
|  | Active Dataset | dataset |
|  | Filter | <none> |
|  | Weight | <none> |
|  | Split File | <none> |
|  | N of Rows in Working Data File | 41 |
| Missing Value Handling | Definition of Missing | User-defined missing values are treated as missing. |
|  | Cases Used | Statistics are based on all cases with valid data for all variables in the model. |
| Syntax | | GLM F3 congruent F3incongruent F4 congruent F4incongruent FZ congruent FZ incongruent C3 congruent C3incongruent C4 congruent C4incongruent CZ congruent CZ incongruent CP1 congruent CP1incongruent CP2 congruent CP2incongruent BY power  /WSFACTOR=electrode 8 Polynomial conflict 2 Polynomial  /METHOD=SSTYPE(3)  /EMMEANS=TABLES(power) COMPARE ADJ(LSD)  /EMMEANS=TABLES(electrode) COMPARE ADJ(LSD)  /EMMEANS=TABLES(conflict) COMPARE ADJ(LSD)  /EMMEANS=TABLES(power*electrode)  /EMMEANS=TABLES(power*conflict)  /EMMEANS=TABLES(electrode*conflict)  /EMMEANS=TABLES(power*electrode*conflict)  /PRINT=DESCRIPTIVE ETASQ OPOWER HOMOGENEITY  /CRITERIA=ALPHA(.05)  /WSDESIGN=electrode conflict electrode*conflict  /DESIGN=power. |
| Resources | Processor Time | 00:00:00.05 |
|  | Elapsed Time | 00:00:00.04 |

| **Within-Subjects Factors** | | |
| --- | --- | --- |
| Measure: MEASURE_1 | | |
| electrode | conflict | Dependent Variable |
| 1 | 1 | F3congruent |
|  | 2 | F3incongruent |
| 2 | 1 | F4congruent |
|  | 2 | F4incongruent |
| 3 | 1 | FZcongruent |
|  | 2 | FZincongruent |
| 4 | 1 | C3congruent |
|  | 2 | C3incongruent |
| 5 | 1 | C4congruent |
|  | 2 | C4incongruent |
| 6 | 1 | CZcongruent |
|  | 2 | CZincongruent |
| 7 | 1 | CP1congruent |
|  | 2 | CP1incongruent |
| 8 | 1 | CP2congruent |
|  | 2 | CP2incongruent |

| **Between-Subjects Factors** | | |
| --- | --- | --- |
|  | | N |
| power | 1.00 | 19 |
|  | 2.00 | 19 |

| **Descriptive Statistics** | | | | |
| --- | --- | --- | --- | --- |
|  | power | Mean | Std. Deviation | N |
| F3congruent | 1.00 | 1.3618 | 4.36806 | 19 |
|  | 2.00 | 1.6684 | 3.64938 | 19 |
|  | Total | 1.5151 | 3.97307 | 38 |
| F3incongruent | 1.00 | .8410 | 4.91577 | 19 |
|  | 2.00 | .5117 | 3.09677 | 19 |
|  | Total | .6763 | 4.05574 | 38 |
| F4congruent | 1.00 | 2.5659 | 4.68614 | 19 |
|  | 2.00 | 1.7837 | 3.46284 | 19 |
|  | Total | 2.1748 | 4.08337 | 38 |
| F4incongruent | 1.00 | 2.1521 | 5.41873 | 19 |
|  | 2.00 | 1.3604 | 3.18432 | 19 |
|  | Total | 1.7563 | 4.40209 | 38 |
| FZ congruent | 1.00 | 1.8380 | 4.74347 | 19 |
|  | 2.00 | 1.4192 | 3.58815 | 19 |
|  | Total | 1.6286 | 4.15387 | 38 |
| FZ incongruent | 1.00 | 1.2767 | 5.65628 | 19 |
|  | 2.00 | .2631 | 3.03188 | 19 |
|  | Total | .7699 | 4.50557 | 38 |
| C3congruent | 1.00 | 3.0403 | 2.37261 | 19 |
|  | 2.00 | 3.3027 | 3.82188 | 19 |
|  | Total | 3.1715 | 3.14042 | 38 |
| C3incongruent | 1.00 | 2.8306 | 2.72263 | 19 |
|  | 2.00 | 2.4334 | 2.86633 | 19 |
|  | Total | 2.6320 | 2.76470 | 38 |
| C4congruent | 1.00 | 4.7282 | 3.22559 | 19 |
|  | 2.00 | 4.4908 | 2.81076 | 19 |
|  | Total | 4.6095 | 2.98655 | 38 |
| C4incongruent | 1.00 | 4.3207 | 3.64164 | 19 |
|  | 2.00 | 3.4342 | 2.20075 | 19 |
|  | Total | 3.8774 | 3.00159 | 38 |
| CZ congruent | 1.00 | 4.5147 | 3.69623 | 19 |
|  | 2.00 | 4.0390 | 4.19474 | 19 |
|  | Total | 4.2768 | 3.90700 | 38 |
| CZ incongruent | 1.00 | 3.6337 | 3.82026 | 19 |
|  | 2.00 | 2.9573 | 3.65672 | 19 |
|  | Total | 3.2955 | 3.70440 | 38 |
| CP1congruent | 1.00 | 5.6592 | 3.42738 | 19 |
|  | 2.00 | 6.0827 | 3.91236 | 19 |
|  | Total | 5.8709 | 3.63418 | 38 |
| CP1incongruent | 1.00 | 5.4867 | 3.46295 | 19 |
|  | 2.00 | 5.1791 | 3.05318 | 19 |
|  | Total | 5.3329 | 3.22386 | 38 |
| CP2congruent | 1.00 | 6.9837 | 3.64680 | 19 |
|  | 2.00 | 6.6894 | 3.48575 | 19 |
|  | Total | 6.8365 | 3.52181 | 38 |
| CP2incongruent | 1.00 | 6.4247 | 3.95479 | 19 |
|  | 2.00 | 5.8165 | 3.03983 | 19 |
|  | Total | 6.1206 | 3.49274 | 38 |

| **Box's Test of Equality of Covariance Matrices^a^** | |
| --- | --- |
| Box's M | 317.339 |
| F | 1.188 |
| df1 | 136 |
| df2 | 4002.174 |
| Sig. | .070 |
| Tests the null hypothesis that the observed covariance matrices of the dependent variables are equal across groups. | |
| a. Design: + power  Within Subjects Design: electrode + conflict + electrode * conflict | |

| **Multivariate Tests^a^** | | | | | | | | | |
| --- | --- | --- | --- | --- | --- | --- | --- | --- | --- |
| Effect | | Value | F | Hypothesis df | Error df | Sig. | Partial Eta Squared | Noncent. Parameter | Observed Power^c^ |
| electrode | Pillai's Trace | .809 | 18.116^b^ | 7.000 | 30.000 | .000 | .809 | 126.814 | 1.000 |
|  | Wilks' Lambda | .191 | 18.116^b^ | 7.000 | 30.000 | .000 | .809 | 126.814 | 1.000 |
|  | Hotelling's Trace | 4.227 | 18.116^b^ | 7.000 | 30.000 | .000 | .809 | 126.814 | 1.000 |
|  | Roy's Largest Root | 4.227 | 18.116^b^ | 7.000 | 30.000 | .000 | .809 | 126.814 | 1.000 |
| electrode * power | Pillai's Trace | .065 | .297^b^ | 7.000 | 30.000 | .950 | .065 | 2.081 | .122 |
|  | Wilks' Lambda | .935 | .297^b^ | 7.000 | 30.000 | .950 | .065 | 2.081 | .122 |
|  | Hotelling's Trace | .069 | .297^b^ | 7.000 | 30.000 | .950 | .065 | 2.081 | .122 |
|  | Roy's Largest Root | .069 | .297^b^ | 7.000 | 30.000 | .950 | .065 | 2.081 | .122 |
| conflict | Pillai's Trace | .166 | 7.165^b^ | 1.000 | 36.000 | .011 | .166 | 7.165 | .740 |
|  | Wilks' Lambda | .834 | 7.165^b^ | 1.000 | 36.000 | .011 | .166 | 7.165 | .740 |
|  | Hotelling's Trace | .199 | 7.165^b^ | 1.000 | 36.000 | .011 | .166 | 7.165 | .740 |
|  | Roy's Largest Root | .199 | 7.165^b^ | 1.000 | 36.000 | .011 | .166 | 7.165 | .740 |
| conflict * power | Pillai's Trace | .022 | .816^b^ | 1.000 | 36.000 | .372 | .022 | .816 | .142 |
|  | Wilks' Lambda | .978 | .816^b^ | 1.000 | 36.000 | .372 | .022 | .816 | .142 |
|  | Hotelling's Trace | .023 | .816^b^ | 1.000 | 36.000 | .372 | .022 | .816 | .142 |
|  | Roy's Largest Root | .023 | .816^b^ | 1.000 | 36.000 | .372 | .022 | .816 | .142 |
| electrode * conflict | Pillai's Trace | .521 | 4.665^b^ | 7.000 | 30.000 | .001 | .521 | 32.654 | .979 |
|  | Wilks' Lambda | .479 | 4.665^b^ | 7.000 | 30.000 | .001 | .521 | 32.654 | .979 |
|  | Hotelling's Trace | 1.088 | 4.665^b^ | 7.000 | 30.000 | .001 | .521 | 32.654 | .979 |
|  | Roy's Largest Root | 1.088 | 4.665^b^ | 7.000 | 30.000 | .001 | .521 | 32.654 | .979 |
| electrode * conflict * power | Pillai's Trace | .200 | 1.070^b^ | 7.000 | 30.000 | .406 | .200 | 7.489 | .379 |
|  | Wilks' Lambda | .800 | 1.070^b^ | 7.000 | 30.000 | .406 | .200 | 7.489 | .379 |
|  | Hotelling's Trace | .250 | 1.070^b^ | 7.000 | 30.000 | .406 | .200 | 7.489 | .379 |
|  | Roy's Largest Root | .250 | 1.070^b^ | 7.000 | 30.000 | .406 | .200 | 7.489 | .379 |
| a. Design: + power  Within Subjects Design: electrode + conflict + electrode * conflict | | | | | | | | | |
| b. Exact statistic | | | | | | | | | |
| c. Computed using alpha = .05 | | | | | | | | | |

| **Mauchly's Test of Sphericity^a^** | | | | | | | |
| --- | --- | --- | --- | --- | --- | --- | --- |
| Measure: MEASURE_1 | | | | | | | |
| Within Subjects Effect | Mauchly's W | Approx. Chi-Square | df | Sig. | Epsilon^b^ | | |
|  |  |  |  |  | Greenhouse-Geisser | Huynh-Feldt | Lower-bound |
| electrode | .000 | 275.013 | 27 | .000 | .300 | .328 | .143 |
| conflict | 1.000 | .000 | 0 | . | 1.000 | 1.000 | 1.000 |
| electrode * conflict | .064 | 92.176 | 27 | .000 | .513 | .592 | .143 |
| Tests the null hypothesis that the error covariance matrix of the orthonormalized transformed dependent variables is proportional to an identity matrix. | | | | | | | |
| a. Design: + power  Within Subjects Design: electrode + conflict + electrode * conflict | | | | | | | |
| b. May be used to adjust the degrees of freedom for the averaged tests of significance. Corrected tests are displayed in the Tests of Within-Subjects Effects table. | | | | | | | |

| **Tests of Within-Subjects Effects** | | | | | | | | | |
| --- | --- | --- | --- | --- | --- | --- | --- | --- | --- |
| Measure: MEASURE_1 | | | | | | | | | |
| Source | | Type III Sum of Squares | df | Mean Square | F | Sig. | Partial Eta Squared | Noncent. Parameter | Observed Power^a^ |
| electrode | Sphericity Assumed | 2101.015 | 7 | 300.145 | 29.585 | .000 | .451 | 207.098 | 1.000 |
|  | Greenhouse-Geisser | 2101.015 | 2.102 | 999.606 | 29.585 | .000 | .451 | 62.184 | 1.000 |
|  | Huynh-Feldt | 2101.015 | 2.297 | 914.607 | 29.585 | .000 | .451 | 67.963 | 1.000 |
|  | Lower-bound | 2101.015 | 1.000 | 2101.015 | 29.585 | .000 | .451 | 29.585 | 1.000 |
| electrode * power | Sphericity Assumed | 14.821 | 7 | 2.117 | .209 | .983 | .006 | 1.461 | .109 |
|  | Greenhouse-Geisser | 14.821 | 2.102 | 7.051 | .209 | .823 | .006 | .439 | .082 |
|  | Huynh-Feldt | 14.821 | 2.297 | 6.452 | .209 | .841 | .006 | .479 | .083 |
|  | Lower-bound | 14.821 | 1.000 | 14.821 | .209 | .651 | .006 | .209 | .073 |
| Error(electrode) | Sphericity Assumed | 2556.548 | 252 | 10.145 |  |  |  |  |  |
|  | Greenhouse-Geisser | 2556.548 | 75.666 | 33.787 |  |  |  |  |  |
|  | Huynh-Feldt | 2556.548 | 82.698 | 30.914 |  |  |  |  |  |
|  | Lower-bound | 2556.548 | 36.000 | 71.015 |  |  |  |  |  |
| conflict | Sphericity Assumed | 75.087 | 1 | 75.087 | 7.165 | .011 | .166 | 7.165 | .740 |
|  | Greenhouse-Geisser | 75.087 | 1.000 | 75.087 | 7.165 | .011 | .166 | 7.165 | .740 |
|  | Huynh-Feldt | 75.087 | 1.000 | 75.087 | 7.165 | .011 | .166 | 7.165 | .740 |
|  | Lower-bound | 75.087 | 1.000 | 75.087 | 7.165 | .011 | .166 | 7.165 | .740 |
| conflict * power | Sphericity Assumed | 8.551 | 1 | 8.551 | .816 | .372 | .022 | .816 | .142 |
|  | Greenhouse-Geisser | 8.551 | 1.000 | 8.551 | .816 | .372 | .022 | .816 | .142 |
|  | Huynh-Feldt | 8.551 | 1.000 | 8.551 | .816 | .372 | .022 | .816 | .142 |
|  | Lower-bound | 8.551 | 1.000 | 8.551 | .816 | .372 | .022 | .816 | .142 |
| Error(conflict) | Sphericity Assumed | 377.263 | 36 | 10.480 |  |  |  |  |  |
|  | Greenhouse-Geisser | 377.263 | 36.000 | 10.480 |  |  |  |  |  |
|  | Huynh-Feldt | 377.263 | 36.000 | 10.480 |  |  |  |  |  |
|  | Lower-bound | 377.263 | 36.000 | 10.480 |  |  |  |  |  |
| electrode * conflict | Sphericity Assumed | 4.865 | 7 | .695 | 1.687 | .113 | .045 | 11.806 | .687 |
|  | Greenhouse-Geisser | 4.865 | 3.589 | 1.356 | 1.687 | .163 | .045 | 6.053 | .477 |
|  | Huynh-Feldt | 4.865 | 4.146 | 1.173 | 1.687 | .154 | .045 | 6.992 | .518 |
|  | Lower-bound | 4.865 | 1.000 | 4.865 | 1.687 | .202 | .045 | 1.687 | .244 |
| electrode * conflict * power | Sphericity Assumed | 2.318 | 7 | .331 | .804 | .585 | .022 | 5.626 | .344 |
|  | Greenhouse-Geisser | 2.318 | 3.589 | .646 | .804 | .513 | .022 | 2.884 | .239 |
|  | Huynh-Feldt | 2.318 | 4.146 | .559 | .804 | .529 | .022 | 3.332 | .257 |
|  | Lower-bound | 2.318 | 1.000 | 2.318 | .804 | .376 | .022 | .804 | .141 |
| Error(electrode*conflict) | Sphericity Assumed | 103.844 | 252 | .412 |  |  |  |  |  |
|  | Greenhouse-Geisser | 103.844 | 129.198 | .804 |  |  |  |  |  |
|  | Huynh-Feldt | 103.844 | 149.255 | .696 |  |  |  |  |  |
|  | Lower-bound | 103.844 | 36.000 | 2.885 |  |  |  |  |  |
| a. Computed using alpha = .05 | | | | | | | | | |

| **Tests of Within-Subjects Contrasts** | | | | | | | | | | |
| --- | --- | --- | --- | --- | --- | --- | --- | --- | --- | --- |
| Measure: MEASURE_1 | | | | | | | | | | |
| Source | electrode | conflict | Type III Sum of Squares | df | Mean Square | F | Sig. | Partial Eta Squared | Noncent. Parameter | Observed Power^a^ |
| electrode | Linear |  | 1909.217 | 1 | 1909.217 | 45.539 | .000 | .558 | 45.539 | 1.000 |
|  | Quadratic |  | 44.384 | 1 | 44.384 | 41.397 | .000 | .535 | 41.397 | 1.000 |
|  | Cubic |  | 2.000 | 1 | 2.000 | .470 | .498 | .013 | .470 | .102 |
|  | Order 4 |  | 1.968 | 1 | 1.968 | .494 | .487 | .014 | .494 | .105 |
|  | Order 5 |  | 11.459 | 1 | 11.459 | 1.377 | .248 | .037 | 1.377 | .208 |
|  | Order 6 |  | 128.411 | 1 | 128.411 | 18.702 | .000 | .342 | 18.702 | .988 |
|  | Order 7 |  | 3.576 | 1 | 3.576 | .780 | .383 | .021 | .780 | .138 |
| electrode * power | Linear |  | .130 | 1 | .130 | .003 | .956 | .000 | .003 | .050 |
|  | Quadratic |  | 1.056 | 1 | 1.056 | .985 | .328 | .027 | .985 | .162 |
|  | Cubic |  | 3.330 | 1 | 3.330 | .782 | .382 | .021 | .782 | .138 |
|  | Order 4 |  | .611 | 1 | .611 | .153 | .698 | .004 | .153 | .067 |
|  | Order 5 |  | 6.602 | 1 | 6.602 | .793 | .379 | .022 | .793 | .140 |
|  | Order 6 |  | 2.023 | 1 | 2.023 | .295 | .591 | .008 | .295 | .083 |
|  | Order 7 |  | 1.069 | 1 | 1.069 | .233 | .632 | .006 | .233 | .076 |
| Error(electrode) | Linear |  | 1509.301 | 36 | 41.925 |  |  |  |  |  |
|  | Quadratic |  | 38.597 | 36 | 1.072 |  |  |  |  |  |
|  | Cubic |  | 153.313 | 36 | 4.259 |  |  |  |  |  |
|  | Order 4 |  | 143.376 | 36 | 3.983 |  |  |  |  |  |
|  | Order 5 |  | 299.618 | 36 | 8.323 |  |  |  |  |  |
|  | Order 6 |  | 247.188 | 36 | 6.866 |  |  |  |  |  |
|  | Order 7 |  | 165.155 | 36 | 4.588 |  |  |  |  |  |
| conflict |  | Linear | 75.087 | 1 | 75.087 | 7.165 | .011 | .166 | 7.165 | .740 |
| conflict * power |  | Linear | 8.551 | 1 | 8.551 | .816 | .372 | .022 | .816 | .142 |
| Error(conflict) |  | Linear | 377.263 | 36 | 10.480 |  |  |  |  |  |
| electrode * conflict | Linear | Linear | .010 | 1 | .010 | .013 | .908 | .000 | .013 | .051 |
|  | Quadratic | Linear | .000 | 1 | .000 | .001 | .982 | .000 | .001 | .050 |
|  | Cubic | Linear | .602 | 1 | .602 | 1.331 | .256 | .036 | 1.331 | .202 |
|  | Order 4 | Linear | .589 | 1 | .589 | 2.141 | .152 | .056 | 2.141 | .296 |
|  | Order 5 | Linear | .016 | 1 | .016 | .031 | .861 | .001 | .031 | .053 |
|  | Order 6 | Linear | 3.501 | 1 | 3.501 | 15.730 | .000 | .304 | 15.730 | .971 |
|  | Order 7 | Linear | .145 | 1 | .145 | .406 | .528 | .011 | .406 | .095 |
| electrode * conflict * power | Linear | Linear | .001 | 1 | .001 | .001 | .975 | .000 | .001 | .050 |
|  | Quadratic | Linear | .067 | 1 | .067 | .218 | .643 | .006 | .218 | .074 |
|  | Cubic | Linear | .170 | 1 | .170 | .375 | .544 | .010 | .375 | .092 |
|  | Order 4 | Linear | .317 | 1 | .317 | 1.151 | .290 | .031 | 1.151 | .181 |
|  | Order 5 | Linear | 1.437 | 1 | 1.437 | 2.748 | .106 | .071 | 2.748 | .365 |
|  | Order 6 | Linear | .082 | 1 | .082 | .369 | .547 | .010 | .369 | .091 |
|  | Order 7 | Linear | .244 | 1 | .244 | .682 | .414 | .019 | .682 | .127 |
| Error(electrode*conflict) | Linear | Linear | 26.824 | 36 | .745 |  |  |  |  |  |
|  | Quadratic | Linear | 11.082 | 36 | .308 |  |  |  |  |  |
|  | Cubic | Linear | 16.294 | 36 | .453 |  |  |  |  |  |
|  | Order 4 | Linear | 9.911 | 36 | .275 |  |  |  |  |  |
|  | Order 5 | Linear | 18.833 | 36 | .523 |  |  |  |  |  |
|  | Order 6 | Linear | 8.013 | 36 | .223 |  |  |  |  |  |
|  | Order 7 | Linear | 12.887 | 36 | .358 |  |  |  |  |  |
| a. Computed using alpha = .05 | | | | | | | | | | |

| **Levene's Test of Equality of Error Variances^a^** | | | | |
| --- | --- | --- | --- | --- |
|  | F | df1 | df2 | Sig. |
| F3congruent | .515 | 1 | 36 | .478 |
| F3incongruent | 4.406 | 1 | 36 | .043 |
| F4congruent | 2.006 | 1 | 36 | .165 |
| F4incongruent | 5.783 | 1 | 36 | .021 |
| FZcongruent | 2.175 | 1 | 36 | .149 |
| FZincongruent | 10.234 | 1 | 36 | .003 |
| C3congruent | 3.466 | 1 | 36 | .071 |
| C3incongruent | .265 | 1 | 36 | .610 |
| C4congruent | .512 | 1 | 36 | .479 |
| C4incongruent | 8.199 | 1 | 36 | .007 |
| CZcongruent | 1.610 | 1 | 36 | .213 |
| CZincongruent | .157 | 1 | 36 | .694 |
| CP1congruent | .673 | 1 | 36 | .417 |
| CP1incongruent | .009 | 1 | 36 | .926 |
| CP2congruent | .002 | 1 | 36 | .964 |
| CP2incongruent | .511 | 1 | 36 | .479 |
| Tests the null hypothesis that the error variance of the dependent variable is equal across groups. | | | | |
| a. Design: + power  Within Subjects Design: electrode + conflict + electrode * conflict | | | | |

| **Tests of Between-Subjects Effects** | | | | | | | | |
| --- | --- | --- | --- | --- | --- | --- | --- | --- |
| Measure: MEASURE_1 | | | | | | | | |
| Transformed Variable: Average | | | | | | | | |
| Source | Type III Sum of Squares | df | Mean Square | F | Sig. | Partial Eta Squared | Noncent. Parameter | Observed Power^a^ |
|  | 7065.949 | 1 | 7065.949 | 50.906 | .000 | .586 | 50.906 | 1.000 |
| power | 23.017 | 1 | 23.017 | .166 | .686 | .005 | .166 | .068 |
| Error | 4996.893 | 36 | 138.803 |  |  |  |  |  |
| a. Computed using alpha = .05 | | | | | | | | |

**Estimated Marginal Means**

**1. power**

| **Estimates** | | | | |
| --- | --- | --- | --- | --- |
| Measure: MEASURE_1 | | | | |
| power | Mean | Std. Error | 95% Confidence Interval | |
|  |  |  | Lower Bound | Upper Bound |
| 1.00 | 3.604 | .676 | 2.233 | 4.974 |
| 2.00 | 3.214 | .676 | 1.844 | 4.585 |

| **Pairwise Comparisons** | | | | | | |
| --- | --- | --- | --- | --- | --- | --- |
| Measure: MEASURE_1 | | | | | | |
| (I) power | (J) power | Mean Difference (I-J) | Std. Error | Sig.^a^ | 95% Confidence Interval for Difference^a^ | |
|  |  |  |  |  | Lower Bound | Upper Bound |
| 1.00 | 2.00 | .389 | .956 | .686 | -1.549 | 2.327 |
| 2.00 | 1.00 | -.389 | .956 | .686 | -2.327 | 1.549 |
| Based on estimated marginal means | | | | | | |
| a. Adjustment for multiple comparisons: Least Significant Difference (equivalent to no adjustments). | | | | | | |

| **Univariate Tests** | | | | | | | | |
| --- | --- | --- | --- | --- | --- | --- | --- | --- |
| Measure: MEASURE_1 | | | | | | | | |
|  | Sum of Squares | df | Mean Square | F | Sig. | Partial Eta Squared | Noncent. Parameter | Observed Power^a^ |
| Contrast | 1.439 | 1 | 1.439 | .166 | .686 | .005 | .166 | .068 |
| Error | 312.306 | 36 | 8.675 |  |  |  |  |  |
| The F tests the effect of power. This test is based on the linearly independent pairwise comparisons among the estimated marginal means. | | | | | | | | |
| a. Computed using alpha = .05 | | | | | | | | |

**2. electrode**

| **Estimates** | | | | |
| --- | --- | --- | --- | --- |
| Measure: MEASURE_1 | | | | |
| electrode | Mean | Std. Error | 95% Confidence Interval | |
|  |  |  | Lower Bound | Upper Bound |
| 1 | 1.096 | .640 | -.202 | 2.394 |
| 2 | 1.966 | .676 | .595 | 3.336 |
| 3 | 1.199 | .692 | -.205 | 2.604 |
| 4 | 2.902 | .464 | 1.960 | 3.843 |
| 5 | 4.243 | .470 | 3.291 | 5.196 |
| 6 | 3.786 | .609 | 2.552 | 5.021 |
| 7 | 5.602 | .544 | 4.498 | 6.705 |
| 8 | 6.479 | .559 | 5.345 | 7.612 |

| **Pairwise Comparisons** | | | | | | |
| --- | --- | --- | --- | --- | --- | --- |
| Measure: MEASURE_1 | | | | | | |
| (I) electrode | (J) electrode | Mean Difference (I-J) | Std. Error | Sig.^b^ | 95% Confidence Interval for Difference^b^ | |
|  |  |  |  |  | Lower Bound | Upper Bound |
| 1 | 2 | -.870 | .464 | .069 | -1.811 | .071 |
|  | 3 | -.104 | .310 | .740 | -.732 | .525 |
|  | 4 | -1.806^*^ | .447 | .000 | -2.712 | -.900 |
|  | 5 | -3.148^*^ | .569 | .000 | -4.302 | -1.993 |
|  | 6 | -2.690^*^ | .587 | .000 | -3.881 | -1.500 |
|  | 7 | -4.506^*^ | .707 | .000 | -5.941 | -3.072 |
|  | 8 | -5.383^*^ | .744 | .000 | -6.892 | -3.873 |
| 2 | 1 | .870 | .464 | .069 | -.071 | 1.811 |
|  | 3 | .766^*^ | .297 | .014 | .165 | 1.368 |
|  | 4 | -.936 | .610 | .134 | -2.174 | .302 |
|  | 5 | -2.278^*^ | .458 | .000 | -3.207 | -1.349 |
|  | 6 | -1.821^*^ | .576 | .003 | -2.989 | -.653 |
|  | 7 | -3.636^*^ | .747 | .000 | -5.151 | -2.122 |
|  | 8 | -4.513^*^ | .689 | .000 | -5.911 | -3.115 |
| 3 | 1 | .104 | .310 | .740 | -.525 | .732 |
|  | 2 | -.766^*^ | .297 | .014 | -1.368 | -.165 |
|  | 4 | -1.702^*^ | .544 | .003 | -2.807 | -.598 |
|  | 5 | -3.044^*^ | .519 | .000 | -4.098 | -1.991 |
|  | 6 | -2.587^*^ | .532 | .000 | -3.666 | -1.508 |
|  | 7 | -4.403^*^ | .732 | .000 | -5.887 | -2.919 |
|  | 8 | -5.279^*^ | .718 | .000 | -6.736 | -3.823 |
| 4 | 1 | 1.806^*^ | .447 | .000 | .900 | 2.712 |
|  | 2 | .936 | .610 | .134 | -.302 | 2.174 |
|  | 3 | 1.702^*^ | .544 | .003 | .598 | 2.807 |
|  | 5 | -1.342^*^ | .400 | .002 | -2.153 | -.530 |
|  | 6 | -.884^*^ | .391 | .030 | -1.677 | -.091 |
|  | 7 | -2.700^*^ | .349 | .000 | -3.407 | -1.993 |
|  | 8 | -3.577^*^ | .447 | .000 | -4.483 | -2.671 |
| 5 | 1 | 3.148^*^ | .569 | .000 | 1.993 | 4.302 |
|  | 2 | 2.278^*^ | .458 | .000 | 1.349 | 3.207 |
|  | 3 | 3.044^*^ | .519 | .000 | 1.991 | 4.098 |
|  | 4 | 1.342^*^ | .400 | .002 | .530 | 2.153 |
|  | 6 | .457 | .370 | .225 | -.293 | 1.208 |
|  | 7 | -1.358^*^ | .413 | .002 | -2.197 | -.520 |
|  | 8 | -2.235^*^ | .291 | .000 | -2.825 | -1.645 |
| 6 | 1 | 2.690^*^ | .587 | .000 | 1.500 | 3.881 |
|  | 2 | 1.821^*^ | .576 | .003 | .653 | 2.989 |
|  | 3 | 2.587^*^ | .532 | .000 | 1.508 | 3.666 |
|  | 4 | .884^*^ | .391 | .030 | .091 | 1.677 |
|  | 5 | -.457 | .370 | .225 | -1.208 | .293 |
|  | 7 | -1.816^*^ | .332 | .000 | -2.489 | -1.143 |
|  | 8 | -2.692^*^ | .331 | .000 | -3.364 | -2.021 |
| 7 | 1 | 4.506^*^ | .707 | .000 | 3.072 | 5.941 |
|  | 2 | 3.636^*^ | .747 | .000 | 2.122 | 5.151 |
|  | 3 | 4.403^*^ | .732 | .000 | 2.919 | 5.887 |
|  | 4 | 2.700^*^ | .349 | .000 | 1.993 | 3.407 |
|  | 5 | 1.358^*^ | .413 | .002 | .520 | 2.197 |
|  | 6 | 1.816^*^ | .332 | .000 | 1.143 | 2.489 |
|  | 8 | -.877^*^ | .245 | .001 | -1.373 | -.381 |
| 8 | 1 | 5.383^*^ | .744 | .000 | 3.873 | 6.892 |
|  | 2 | 4.513^*^ | .689 | .000 | 3.115 | 5.911 |
|  | 3 | 5.279^*^ | .718 | .000 | 3.823 | 6.736 |
|  | 4 | 3.577^*^ | .447 | .000 | 2.671 | 4.483 |
|  | 5 | 2.235^*^ | .291 | .000 | 1.645 | 2.825 |
|  | 6 | 2.692^*^ | .331 | .000 | 2.021 | 3.364 |
|  | 7 | .877^*^ | .245 | .001 | .381 | 1.373 |
| Based on estimated marginal means | | | | | | |
| *. The mean difference is significant at the .05 level. | | | | | | |
| b. Adjustment for multiple comparisons: Least Significant Difference (equivalent to no adjustments). | | | | | | |

| **Multivariate Tests** | | | | | | | | |
| --- | --- | --- | --- | --- | --- | --- | --- | --- |
|  | Value | F | Hypothesis df | Error df | Sig. | Partial Eta Squared | Noncent. Parameter | Observed Power^b^ |
| Pillai's trace | .809 | 18.116^a^ | 7.000 | 30.000 | .000 | .809 | 126.814 | 1.000 |
| Wilks' lambda | .191 | 18.116^a^ | 7.000 | 30.000 | .000 | .809 | 126.814 | 1.000 |
| Hotelling's trace | 4.227 | 18.116^a^ | 7.000 | 30.000 | .000 | .809 | 126.814 | 1.000 |
| Roy's largest root | 4.227 | 18.116^a^ | 7.000 | 30.000 | .000 | .809 | 126.814 | 1.000 |
| Each F tests the multivariate effect of electrode. These tests are based on the linearly independent pairwise comparisons among the estimated marginal means. | | | | | | | | |
| a. Exact statistic | | | | | | | | |
| b. Computed using alpha = .05 | | | | | | | | |

**3. conflict**

| **Estimates** | | | | |
| --- | --- | --- | --- | --- |
| Measure: MEASURE_1 | | | | |
| conflict | Mean | Std. Error | 95% Confidence Interval | |
|  |  |  | Lower Bound | Upper Bound |
| 1 | 3.760 | .501 | 2.745 | 4.776 |
| 2 | 3.058 | .490 | 2.063 | 4.052 |

| **Pairwise Comparisons** | | | | | | |
| --- | --- | --- | --- | --- | --- | --- |
| Measure: MEASURE_1 | | | | | | |
| (I) conflict | (J) conflict | Mean Difference (I-J) | Std. Error | Sig.^b^ | 95% Confidence Interval for Difference^b^ | |
|  |  |  |  |  | Lower Bound | Upper Bound |
| 1 | 2 | .703^*^ | .263 | .011 | .170 | 1.235 |
| 2 | 1 | -.703^*^ | .263 | .011 | -1.235 | -.170 |
| Based on estimated marginal means | | | | | | |
| *. The mean difference is significant at the .05 level. | | | | | | |
| b. Adjustment for multiple comparisons: Least Significant Difference (equivalent to no adjustments). | | | | | | |

| **Multivariate Tests** | | | | | | | | |
| --- | --- | --- | --- | --- | --- | --- | --- | --- |
|  | Value | F | Hypothesis df | Error df | Sig. | Partial Eta Squared | Noncent. Parameter | Observed Power^b^ |
| Pillai's trace | .166 | 7.165^a^ | 1.000 | 36.000 | .011 | .166 | 7.165 | .740 |
| Wilks' lambda | .834 | 7.165^a^ | 1.000 | 36.000 | .011 | .166 | 7.165 | .740 |
| Hotelling's trace | .199 | 7.165^a^ | 1.000 | 36.000 | .011 | .166 | 7.165 | .740 |
| Roy's largest root | .199 | 7.165^a^ | 1.000 | 36.000 | .011 | .166 | 7.165 | .740 |
| Each F tests the multivariate effect of conflict. These tests are based on the linearly independent pairwise comparisons among the estimated marginal means. | | | | | | | | |
| a. Exact statistic | | | | | | | | |
| b. Computed using alpha = .05 | | | | | | | | |

| **4. power * electrode** | | | | | |
| --- | --- | --- | --- | --- | --- |
| Measure: MEASURE_1 | | | | | |
| power | electrode | Mean | Std. Error | 95% Confidence Interval | |
|  |  |  |  | Lower Bound | Upper Bound |
| 1.00 | 1 | 1.101 | .905 | -.734 | 2.937 |
|  | 2 | 2.359 | .956 | .421 | 4.297 |
|  | 3 | 1.557 | .979 | -.429 | 3.543 |
|  | 4 | 2.935 | .656 | 1.604 | 4.267 |
|  | 5 | 4.524 | .664 | 3.177 | 5.871 |
|  | 6 | 4.074 | .861 | 2.329 | 5.820 |
|  | 7 | 5.573 | .769 | 4.012 | 7.134 |
|  | 8 | 6.704 | .791 | 5.101 | 8.308 |
| 2.00 | 1 | 1.090 | .905 | -.746 | 2.926 |
|  | 2 | 1.572 | .956 | -.366 | 3.510 |
|  | 3 | .841 | .979 | -1.145 | 2.827 |
|  | 4 | 2.868 | .656 | 1.537 | 4.199 |
|  | 5 | 3.963 | .664 | 2.616 | 5.310 |
|  | 6 | 3.498 | .861 | 1.753 | 5.244 |
|  | 7 | 5.631 | .769 | 4.070 | 7.192 |
|  | 8 | 6.253 | .791 | 4.650 | 7.856 |

| **5. power * conflict** | | | | | |
| --- | --- | --- | --- | --- | --- |
| Measure: MEASURE_1 | | | | | |
| power | conflict | Mean | Std. Error | 95% Confidence Interval | |
|  |  |  |  | Lower Bound | Upper Bound |
| 1.00 | 1 | 3.836 | .708 | 2.401 | 5.272 |
|  | 2 | 3.371 | .693 | 1.964 | 4.777 |
| 2.00 | 1 | 3.684 | .708 | 2.249 | 5.120 |
|  | 2 | 2.744 | .693 | 1.338 | 4.151 |

| **6. electrode * conflict** | | | | | |
| --- | --- | --- | --- | --- | --- |
| Measure: MEASURE_1 | | | | | |
| electrode | conflict | Mean | Std. Error | 95% Confidence Interval | |
|  |  |  |  | Lower Bound | Upper Bound |
| 1 | 1 | 1.515 | .653 | .191 | 2.839 |
|  | 2 | .676 | .666 | -.675 | 2.028 |
| 2 | 1 | 2.175 | .668 | .819 | 3.530 |
|  | 2 | 1.756 | .721 | .294 | 3.218 |
| 3 | 1 | 1.629 | .682 | .245 | 3.012 |
|  | 2 | .770 | .736 | -.723 | 2.263 |
| 4 | 1 | 3.171 | .516 | 2.125 | 4.218 |
|  | 2 | 2.632 | .453 | 1.712 | 3.552 |
| 5 | 1 | 4.610 | .491 | 3.614 | 5.605 |
|  | 2 | 3.877 | .488 | 2.888 | 4.867 |
| 6 | 1 | 4.277 | .641 | 2.976 | 5.577 |
|  | 2 | 3.296 | .607 | 2.065 | 4.526 |
| 7 | 1 | 5.871 | .597 | 4.661 | 7.081 |
|  | 2 | 5.333 | .530 | 4.259 | 6.407 |
| 8 | 1 | 6.837 | .579 | 5.663 | 8.010 |
|  | 2 | 6.121 | .572 | 4.960 | 7.281 |

| **7. power * electrode * conflict** | | | | | | |
| --- | --- | --- | --- | --- | --- | --- |
| Measure: MEASURE_1 | | | | | | |
| power | electrode | conflict | Mean | Std. Error | 95% Confidence Interval | |
|  |  |  |  |  | Lower Bound | Upper Bound |
| 1.00 | 1 | 1 | 1.362 | .923 | -.511 | 3.234 |
|  |  | 2 | .841 | .942 | -1.070 | 2.752 |
|  | 2 | 1 | 2.566 | .945 | .649 | 4.483 |
|  |  | 2 | 2.152 | 1.020 | .084 | 4.220 |
|  | 3 | 1 | 1.838 | .965 | -.119 | 3.795 |
|  |  | 2 | 1.277 | 1.041 | -.835 | 3.388 |
|  | 4 | 1 | 3.040 | .730 | 1.560 | 4.520 |
|  |  | 2 | 2.831 | .641 | 1.530 | 4.131 |
|  | 5 | 1 | 4.728 | .694 | 3.321 | 6.136 |
|  |  | 2 | 4.321 | .690 | 2.921 | 5.721 |
|  | 6 | 1 | 4.515 | .907 | 2.675 | 6.354 |
|  |  | 2 | 3.634 | .858 | 1.894 | 5.374 |
|  | 7 | 1 | 5.659 | .844 | 3.948 | 7.370 |
|  |  | 2 | 5.487 | .749 | 3.968 | 7.006 |
|  | 8 | 1 | 6.984 | .818 | 5.324 | 8.643 |
|  |  | 2 | 6.425 | .809 | 4.784 | 8.066 |
| 2.00 | 1 | 1 | 1.668 | .923 | -.204 | 3.541 |
|  |  | 2 | .512 | .942 | -1.400 | 2.423 |
|  | 2 | 1 | 1.784 | .945 | -.133 | 3.701 |
|  |  | 2 | 1.360 | 1.020 | -.707 | 3.428 |
|  | 3 | 1 | 1.419 | .965 | -.538 | 3.376 |
|  |  | 2 | .263 | 1.041 | -1.848 | 2.375 |
|  | 4 | 1 | 3.303 | .730 | 1.823 | 4.783 |
|  |  | 2 | 2.433 | .641 | 1.133 | 3.734 |
|  | 5 | 1 | 4.491 | .694 | 3.083 | 5.898 |
|  |  | 2 | 3.434 | .690 | 2.034 | 4.834 |
|  | 6 | 1 | 4.039 | .907 | 2.200 | 5.878 |
|  |  | 2 | 2.957 | .858 | 1.217 | 4.697 |
|  | 7 | 1 | 6.083 | .844 | 4.371 | 7.794 |
|  |  | 2 | 5.179 | .749 | 3.660 | 6.698 |
|  | 8 | 1 | 6.689 | .818 | 5.030 | 8.349 |
|  |  | 2 | 5.816 | .809 | 4.175 | 7.458 |

**Only happy faces in P1**

GLM P7 congruent P7incongruent P8 congruent P8incongruent BY power

/WSFACTOR=electrode 2 Polynomial conflict 2 Polynomial

/METHOD=SSTYPE(3)

/POSTHOC=power(BTUKEY LSD BONFERRONI)

/EMMEANS=TABLES(power) COMPARE ADJ(LSD)

/EMMEANS=TABLES(electrode) COMPARE ADJ(LSD)

/EMMEANS=TABLES(conflict) COMPARE ADJ(LSD)

/EMMEANS=TABLES(power*electrode)

/EMMEANS=TABLES(power*conflict)

/EMMEANS=TABLES(electrode*conflict)

/EMMEANS=TABLES(power*electrode*conflict)

/EMMEANS=TABLES(power*electrode*conflict) COMPARE(conflict) ADJ(LSD)

/PRINT=DESCRIPTIVE ETASQ OPOWER HOMOGENEITY

/CRITERIA=ALPHA(.05)

/WSDESIGN=electrode conflict electrode*conflict

/DESIGN=power.

**General Linear Model**

| **Notes** | | |
| --- | --- | --- |
| Output Created | | 12-JAN-2021 19:22:03 |
| Comments | |  |
| Input | Data | E:\11.17 \ERP data\p1 happy.sav |
|  | Active Dataset | dataset |
|  | Filter | <none> |
|  | Weight | <none> |
|  | Split File | <none> |
|  | N of Rows in Working Data File | 41 |
| Missing Value Handling | Definition of Missing | User-defined missing values are treated as missing. |
|  | Cases Used | Statistics are based on all cases with valid data for all variables in the model. |
| Syntax | | GLM P7 congruent P7incongruent P8 congruent P8incongruent BY power  /WSFACTOR=electrode 2 Polynomial conflict 2 Polynomial  /METHOD=SSTYPE(3)  /POSTHOC=power(BTUKEY LSD BONFERRONI)  /EMMEANS=TABLES(power) COMPARE ADJ(LSD)  /EMMEANS=TABLES(electrode) COMPARE ADJ(LSD)  /EMMEANS=TABLES(conflict) COMPARE ADJ(LSD)  /EMMEANS=TABLES(power*electrode)  /EMMEANS=TABLES(power*conflict)  /EMMEANS=TABLES(electrode*conflict)  /EMMEANS=TABLES(power*electrode*conflict)  /EMMEANS=TABLES(power*electrode*conflict) COMPARE(conflict) ADJ(LSD)  /PRINT=DESCRIPTIVE ETASQ OPOWER HOMOGENEITY  /CRITERIA=ALPHA(.05)  /WSDESIGN=electrode conflict electrode*conflict  /DESIGN=power. |
| Resources | Processor Time | 00:00:00.09 |
|  | Elapsed Time | 00:00:00.07 |

| **Warnings** |
| --- |
| Post hoc tests are not performed for power because there are fewer than three groups. |

| **Within-Subjects Factors** | | |
| --- | --- | --- |
| Measure: MEASURE_1 | | |
| electrode | conflict | Dependent Variable |
| 1 | 1 | P7congruent |
|  | 2 | P7incongruent |
| 2 | 1 | P8congruent |
|  | 2 | P8incongruent |

| **Between-Subjects Factors** | | |
| --- | --- | --- |
|  | | N |
| power | 1.00 | 19 |
|  | 2.00 | 19 |

| **Descriptive Statistics** | | | | |
| --- | --- | --- | --- | --- |
|  | power | Mean | Std. Deviation | N |
| P7congruent | 1.00 | -1.3347 | 3.33157 | 19 |
|  | 2.00 | .0294 | 1.90070 | 19 |
|  | Total | -.6526 | 2.76315 | 38 |
| P7incongruent | 1.00 | -1.6830 | 2.97954 | 19 |
|  | 2.00 | .0328 | 2.35712 | 19 |
|  | Total | -.8251 | 2.78885 | 38 |
| P8congruent | 1.00 | -1.6496 | 4.43204 | 19 |
|  | 2.00 | .6286 | 3.77291 | 19 |
|  | Total | -.5105 | 4.22063 | 38 |
| P8incongruent | 1.00 | -1.7280 | 4.01424 | 19 |
|  | 2.00 | .1115 | 3.95426 | 19 |
|  | Total | -.8082 | 4.03917 | 38 |

| **Box's Test of Equality of Covariance Matrices^a^** | |
| --- | --- |
| Box's M | 15.689 |
| F | 1.379 |
| df1 | 10 |
| df2 | 6196.016 |
| Sig. | .183 |
| Tests the null hypothesis that the observed covariance matrices of the dependent variables are equal across groups. | |
| a. Design: + power  Within Subjects Design: electrode + conflict + electrode * conflict | |

| **Multivariate Tests^a^** | | | | | | | | | |
| --- | --- | --- | --- | --- | --- | --- | --- | --- | --- |
| Effect | | Value | F | Hypothesis df | Error df | Sig. | Partial Eta Squared | Noncent. Parameter | Observed Power^c^ |
| electrode | Pillai's Trace | .001 | .024^b^ | 1.000 | 36.000 | .878 | .001 | .024 | .053 |
|  | Wilks' Lambda | .999 | .024^b^ | 1.000 | 36.000 | .878 | .001 | .024 | .053 |
|  | Hotelling's Trace | .001 | .024^b^ | 1.000 | 36.000 | .878 | .001 | .024 | .053 |
|  | Roy's Largest Root | .001 | .024^b^ | 1.000 | 36.000 | .878 | .001 | .024 | .053 |
| electrode * power | Pillai's Trace | .007 | .256^b^ | 1.000 | 36.000 | .616 | .007 | .256 | .078 |
|  | Wilks' Lambda | .993 | .256^b^ | 1.000 | 36.000 | .616 | .007 | .256 | .078 |
|  | Hotelling's Trace | .007 | .256^b^ | 1.000 | 36.000 | .616 | .007 | .256 | .078 |
|  | Roy's Largest Root | .007 | .256^b^ | 1.000 | 36.000 | .616 | .007 | .256 | .078 |
| conflict | Pillai's Trace | .072 | 2.784^b^ | 1.000 | 36.000 | .104 | .072 | 2.784 | .369 |
|  | Wilks' Lambda | .928 | 2.784^b^ | 1.000 | 36.000 | .104 | .072 | 2.784 | .369 |
|  | Hotelling's Trace | .077 | 2.784^b^ | 1.000 | 36.000 | .104 | .072 | 2.784 | .369 |
|  | Roy's Largest Root | .077 | 2.784^b^ | 1.000 | 36.000 | .104 | .072 | 2.784 | .369 |
| conflict * power | Pillai's Trace | .001 | .024^b^ | 1.000 | 36.000 | .878 | .001 | .024 | .053 |
|  | Wilks' Lambda | .999 | .024^b^ | 1.000 | 36.000 | .878 | .001 | .024 | .053 |
|  | Hotelling's Trace | .001 | .024^b^ | 1.000 | 36.000 | .878 | .001 | .024 | .053 |
|  | Roy's Largest Root | .001 | .024^b^ | 1.000 | 36.000 | .878 | .001 | .024 | .053 |
| electrode * conflict | Pillai's Trace | .016 | .573^b^ | 1.000 | 36.000 | .454 | .016 | .573 | .114 |
|  | Wilks' Lambda | .984 | .573^b^ | 1.000 | 36.000 | .454 | .016 | .573 | .114 |
|  | Hotelling's Trace | .016 | .573^b^ | 1.000 | 36.000 | .454 | .016 | .573 | .114 |
|  | Roy's Largest Root | .016 | .573^b^ | 1.000 | 36.000 | .454 | .016 | .573 | .114 |
| electrode * conflict * power | Pillai's Trace | .137 | 5.700^b^ | 1.000 | 36.000 | .022 | .137 | 5.700 | .642 |
|  | Wilks' Lambda | .863 | 5.700^b^ | 1.000 | 36.000 | .022 | .137 | 5.700 | .642 |
|  | Hotelling's Trace | .158 | 5.700^b^ | 1.000 | 36.000 | .022 | .137 | 5.700 | .642 |
|  | Roy's Largest Root | .158 | 5.700^b^ | 1.000 | 36.000 | .022 | .137 | 5.700 | .642 |
| a. Design: + power  Within Subjects Design: electrode + conflict + electrode * conflict | | | | | | | | | |
| b. Exact statistic | | | | | | | | | |
| c. Computed using alpha = .05 | | | | | | | | | |

| **Mauchly's Test of Sphericity^a^** | | | | | | | |
| --- | --- | --- | --- | --- | --- | --- | --- |
| Measure: MEASURE_1 | | | | | | | |
| Within Subjects Effect | Mauchly's W | Approx. Chi-Square | df | Sig. | Epsilon^b^ | | |
|  |  |  |  |  | Greenhouse-Geisser | Huynh-Feldt | Lower-bound |
| electrode | 1.000 | .000 | 0 | . | 1.000 | 1.000 | 1.000 |
| conflict | 1.000 | .000 | 0 | . | 1.000 | 1.000 | 1.000 |
| electrode * conflict | 1.000 | .000 | 0 | . | 1.000 | 1.000 | 1.000 |
| Tests the null hypothesis that the error covariance matrix of the orthonormalized transformed dependent variables is proportional to an identity matrix. | | | | | | | |
| a. Design: + power  Within Subjects Design: electrode + conflict + electrode * conflict | | | | | | | |
| b. May be used to adjust the degrees of freedom for the averaged tests of significance. Corrected tests are displayed in the Tests of Within-Subjects Effects table. | | | | | | | |

| **Tests of Within-Subjects Effects** | | | | | | | | | |
| --- | --- | --- | --- | --- | --- | --- | --- | --- | --- |
| Measure: MEASURE_1 | | | | | | | | | |
| Source | | Type III Sum of Squares | df | Mean Square | F | Sig. | Partial Eta Squared | Noncent. Parameter | Observed Power^a^ |
| electrode | Sphericity Assumed | .240 | 1 | .240 | .024 | .878 | .001 | .024 | .053 |
|  | Greenhouse-Geisser | .240 | 1.000 | .240 | .024 | .878 | .001 | .024 | .053 |
|  | Huynh-Feldt | .240 | 1.000 | .240 | .024 | .878 | .001 | .024 | .053 |
|  | Lower-bound | .240 | 1.000 | .240 | .024 | .878 | .001 | .024 | .053 |
| electrode * power | Sphericity Assumed | 2.557 | 1 | 2.557 | .256 | .616 | .007 | .256 | .078 |
|  | Greenhouse-Geisser | 2.557 | 1.000 | 2.557 | .256 | .616 | .007 | .256 | .078 |
|  | Huynh-Feldt | 2.557 | 1.000 | 2.557 | .256 | .616 | .007 | .256 | .078 |
|  | Lower-bound | 2.557 | 1.000 | 2.557 | .256 | .616 | .007 | .256 | .078 |
| Error(electrode) | Sphericity Assumed | 359.343 | 36 | 9.982 |  |  |  |  |  |
|  | Greenhouse-Geisser | 359.343 | 36.000 | 9.982 |  |  |  |  |  |
|  | Huynh-Feldt | 359.343 | 36.000 | 9.982 |  |  |  |  |  |
|  | Lower-bound | 359.343 | 36.000 | 9.982 |  |  |  |  |  |
| conflict | Sphericity Assumed | 2.101 | 1 | 2.101 | 2.784 | .104 | .072 | 2.784 | .369 |
|  | Greenhouse-Geisser | 2.101 | 1.000 | 2.101 | 2.784 | .104 | .072 | 2.784 | .369 |
|  | Huynh-Feldt | 2.101 | 1.000 | 2.101 | 2.784 | .104 | .072 | 2.784 | .369 |
|  | Lower-bound | 2.101 | 1.000 | 2.101 | 2.784 | .104 | .072 | 2.784 | .369 |
| conflict * power | Sphericity Assumed | .018 | 1 | .018 | .024 | .878 | .001 | .024 | .053 |
|  | Greenhouse-Geisser | .018 | 1.000 | .018 | .024 | .878 | .001 | .024 | .053 |
|  | Huynh-Feldt | .018 | 1.000 | .018 | .024 | .878 | .001 | .024 | .053 |
|  | Lower-bound | .018 | 1.000 | .018 | .024 | .878 | .001 | .024 | .053 |
| Error(conflict) | Sphericity Assumed | 27.165 | 36 | .755 |  |  |  |  |  |
|  | Greenhouse-Geisser | 27.165 | 36.000 | .755 |  |  |  |  |  |
|  | Huynh-Feldt | 27.165 | 36.000 | .755 |  |  |  |  |  |
|  | Lower-bound | 27.165 | 36.000 | .755 |  |  |  |  |  |
| electrode * conflict | Sphericity Assumed | .149 | 1 | .149 | .573 | .454 | .016 | .573 | .114 |
|  | Greenhouse-Geisser | .149 | 1.000 | .149 | .573 | .454 | .016 | .573 | .114 |
|  | Huynh-Feldt | .149 | 1.000 | .149 | .573 | .454 | .016 | .573 | .114 |
|  | Lower-bound | .149 | 1.000 | .149 | .573 | .454 | .016 | .573 | .114 |
| electrode * conflict * power | Sphericity Assumed | 1.484 | 1 | 1.484 | 5.700 | .022 | .137 | 5.700 | .642 |
|  | Greenhouse-Geisser | 1.484 | 1.000 | 1.484 | 5.700 | .022 | .137 | 5.700 | .642 |
|  | Huynh-Feldt | 1.484 | 1.000 | 1.484 | 5.700 | .022 | .137 | 5.700 | .642 |
|  | Lower-bound | 1.484 | 1.000 | 1.484 | 5.700 | .022 | .137 | 5.700 | .642 |
| Error(electrode*conflict) | Sphericity Assumed | 9.371 | 36 | .260 |  |  |  |  |  |
|  | Greenhouse-Geisser | 9.371 | 36.000 | .260 |  |  |  |  |  |
|  | Huynh-Feldt | 9.371 | 36.000 | .260 |  |  |  |  |  |
|  | Lower-bound | 9.371 | 36.000 | .260 |  |  |  |  |  |
| a. Computed using alpha = .05 | | | | | | | | | |

| **Tests of Within-Subjects Contrasts** | | | | | | | | | | |
| --- | --- | --- | --- | --- | --- | --- | --- | --- | --- | --- |
| Measure: MEASURE_1 | | | | | | | | | | |
| Source | electrode | conflict | Type III Sum of Squares | df | Mean Square | F | Sig. | Partial Eta Squared | Noncent. Parameter | Observed Power^a^ |
| electrode | Linear |  | .240 | 1 | .240 | .024 | .878 | .001 | .024 | .053 |
| electrode * power | Linear |  | 2.557 | 1 | 2.557 | .256 | .616 | .007 | .256 | .078 |
| Error(electrode) | Linear |  | 359.343 | 36 | 9.982 |  |  |  |  |  |
| conflict |  | Linear | 2.101 | 1 | 2.101 | 2.784 | .104 | .072 | 2.784 | .369 |
| conflict * power |  | Linear | .018 | 1 | .018 | .024 | .878 | .001 | .024 | .053 |
| Error(conflict) |  | Linear | 27.165 | 36 | .755 |  |  |  |  |  |
| electrode * conflict | Linear | Linear | .149 | 1 | .149 | .573 | .454 | .016 | .573 | .114 |
| electrode * conflict * power | Linear | Linear | 1.484 | 1 | 1.484 | 5.700 | .022 | .137 | 5.700 | .642 |
| Error(electrode*conflict) | Linear | Linear | 9.371 | 36 | .260 |  |  |  |  |  |
| a. Computed using alpha = .05 | | | | | | | | | | |

| **Levene's Test of Equality of Error Variances^a^** | | | | |
| --- | --- | --- | --- | --- |
|  | F | df1 | df2 | Sig. |
| P7congruent | 7.017 | 1 | 36 | .012 |
| P7incongruent | 1.454 | 1 | 36 | .236 |
| P8congruent | .297 | 1 | 36 | .589 |
| P8incongruent | .000 | 1 | 36 | .992 |
| Tests the null hypothesis that the error variance of the dependent variable is equal across groups. | | | | |
| a. Design: + power  Within Subjects Design: electrode + conflict + electrode * conflict | | | | |

| **Tests of Between-Subjects Effects** | | | | | | | | |
| --- | --- | --- | --- | --- | --- | --- | --- | --- |
| Measure: MEASURE_1 | | | | | | | | |
| Transformed Variable: Average | | | | | | | | |
| Source | Type III Sum of Squares | df | Mean Square | F | Sig. | Partial Eta Squared | Noncent. Parameter | Observed Power^a^ |
|  | 74.291 | 1 | 74.291 | 2.042 | .162 | .054 | 2.042 | .285 |
| power | 123.037 | 1 | 123.037 | 3.381 | .074 | .086 | 3.381 | .432 |
| Error | 1310.049 | 36 | 36.390 |  |  |  |  |  |
| a. Computed using alpha = .05 | | | | | | | | |

**Estimated Marginal Means**

**1. power**

| **Estimates** | | | | |
| --- | --- | --- | --- | --- |
| Measure: MEASURE_1 | | | | |
| power | Mean | Std. Error | 95% Confidence Interval | |
|  |  |  | Lower Bound | Upper Bound |
| 1.00 | -1.599 | .692 | -3.002 | -.195 |
| 2.00 | .201 | .692 | -1.203 | 1.604 |

| **Pairwise Comparisons** | | | | | | |
| --- | --- | --- | --- | --- | --- | --- |
| Measure: MEASURE_1 | | | | | | |
| (I) power | (J) power | Mean Difference (I-J) | Std. Error | Sig.^a^ | 95% Confidence Interval for Difference^a^ | |
|  |  |  |  |  | Lower Bound | Upper Bound |
| 1.00 | 2.00 | -1.799 | .979 | .074 | -3.784 | .185 |
| 2.00 | 1.00 | 1.799 | .979 | .074 | -.185 | 3.784 |
| Based on estimated marginal means | | | | | | |
| a. Adjustment for multiple comparisons: Least Significant Difference (equivalent to no adjustments). | | | | | | |

| **Univariate Tests** | | | | | | | | |
| --- | --- | --- | --- | --- | --- | --- | --- | --- |
| Measure: MEASURE_1 | | | | | | | | |
|  | Sum of Squares | df | Mean Square | F | Sig. | Partial Eta Squared | Noncent. Parameter | Observed Power^a^ |
| Contrast | 30.759 | 1 | 30.759 | 3.381 | .074 | .086 | 3.381 | .432 |
| Error | 327.512 | 36 | 9.098 |  |  |  |  |  |
| The F tests the effect of power. This test is based on the linearly independent pairwise comparisons among the estimated marginal means. | | | | | | | | |
| a. Computed using alpha = .05 | | | | | | | | |

**2. electrode**

| **Estimates** | | | | |
| --- | --- | --- | --- | --- |
| Measure: MEASURE_1 | | | | |
| electrode | Mean | Std. Error | 95% Confidence Interval | |
|  |  |  | Lower Bound | Upper Bound |
| 1 | -.739 | .430 | -1.611 | .133 |
| 2 | -.659 | .652 | -1.982 | .663 |

| **Pairwise Comparisons** | | | | | | |
| --- | --- | --- | --- | --- | --- | --- |
| Measure: MEASURE_1 | | | | | | |
| (I) electrode | (J) electrode | Mean Difference (I-J) | Std. Error | Sig.^a^ | 95% Confidence Interval for Difference^a^ | |
|  |  |  |  |  | Lower Bound | Upper Bound |
| 1 | 2 | -.079 | .513 | .878 | -1.119 | .960 |
| 2 | 1 | .079 | .513 | .878 | -.960 | 1.119 |
| Based on estimated marginal means | | | | | | |
| a. Adjustment for multiple comparisons: Least Significant Difference (equivalent to no adjustments). | | | | | | |

| **Multivariate Tests** | | | | | | | | |
| --- | --- | --- | --- | --- | --- | --- | --- | --- |
|  | Value | F | Hypothesis df | Error df | Sig. | Partial Eta Squared | Noncent. Parameter | Observed Power^b^ |
| Pillai's trace | .001 | .024^a^ | 1.000 | 36.000 | .878 | .001 | .024 | .053 |
| Wilks' lambda | .999 | .024^a^ | 1.000 | 36.000 | .878 | .001 | .024 | .053 |
| Hotelling's trace | .001 | .024^a^ | 1.000 | 36.000 | .878 | .001 | .024 | .053 |
| Roy's largest root | .001 | .024^a^ | 1.000 | 36.000 | .878 | .001 | .024 | .053 |
| Each F tests the multivariate effect of electrode. These tests are based on the linearly independent pairwise comparisons among the estimated marginal means. | | | | | | | | |
| a. Exact statistic | | | | | | | | |
| b. Computed using alpha = .05 | | | | | | | | |

**3. conflict**

| **Estimates** | | | | |
| --- | --- | --- | --- | --- |
| Measure: MEASURE_1 | | | | |
| conflict | Mean | Std. Error | 95% Confidence Interval | |
|  |  |  | Lower Bound | Upper Bound |
| 1 | -.582 | .503 | -1.602 | .439 |
| 2 | -.817 | .486 | -1.801 | .168 |

| **Pairwise Comparisons** | | | | | | |
| --- | --- | --- | --- | --- | --- | --- |
| Measure: MEASURE_1 | | | | | | |
| (I) conflict | (J) conflict | Mean Difference (I-J) | Std. Error | Sig.^a^ | 95% Confidence Interval for Difference^a^ | |
|  |  |  |  |  | Lower Bound | Upper Bound |
| 1 | 2 | .235 | .141 | .104 | -.051 | .521 |
| 2 | 1 | -.235 | .141 | .104 | -.521 | .051 |
| Based on estimated marginal means | | | | | | |
| a. Adjustment for multiple comparisons: Least Significant Difference (equivalent to no adjustments). | | | | | | |

| **Multivariate Tests** | | | | | | | | |
| --- | --- | --- | --- | --- | --- | --- | --- | --- |
|  | Value | F | Hypothesis df | Error df | Sig. | Partial Eta Squared | Noncent. Parameter | Observed Power^b^ |
| Pillai's trace | .072 | 2.784^a^ | 1.000 | 36.000 | .104 | .072 | 2.784 | .369 |
| Wilks' lambda | .928 | 2.784^a^ | 1.000 | 36.000 | .104 | .072 | 2.784 | .369 |
| Hotelling's trace | .077 | 2.784^a^ | 1.000 | 36.000 | .104 | .072 | 2.784 | .369 |
| Roy's largest root | .077 | 2.784^a^ | 1.000 | 36.000 | .104 | .072 | 2.784 | .369 |
| Each F tests the multivariate effect of conflict. These tests are based on the linearly independent pairwise comparisons among the estimated marginal means. | | | | | | | | |
| a. Exact statistic | | | | | | | | |
| b. Computed using alpha = .05 | | | | | | | | |

| **4. power * electrode** | | | | | |
| --- | --- | --- | --- | --- | --- |
| Measure: MEASURE_1 | | | | | |
| power | electrode | Mean | Std. Error | 95% Confidence Interval | |
|  |  |  |  | Lower Bound | Upper Bound |
| 1.00 | 1 | -1.509 | .608 | -2.742 | -.276 |
|  | 2 | -1.689 | .922 | -3.559 | .182 |
| 2.00 | 1 | .031 | .608 | -1.202 | 1.264 |
|  | 2 | .370 | .922 | -1.501 | 2.241 |

| **5. power * conflict** | | | | | |
| --- | --- | --- | --- | --- | --- |
| Measure: MEASURE_1 | | | | | |
| power | conflict | Mean | Std. Error | 95% Confidence Interval | |
|  |  |  |  | Lower Bound | Upper Bound |
| 1.00 | 1 | -1.492 | .711 | -2.935 | -.049 |
|  | 2 | -1.705 | .687 | -3.098 | -.313 |
| 2.00 | 1 | .329 | .711 | -1.114 | 1.772 |
|  | 2 | .072 | .687 | -1.320 | 1.465 |

| **6. electrode * conflict** | | | | | |
| --- | --- | --- | --- | --- | --- |
| Measure: MEASURE_1 | | | | | |
| electrode | conflict | Mean | Std. Error | 95% Confidence Interval | |
|  |  |  |  | Lower Bound | Upper Bound |
| 1 | 1 | -.653 | .440 | -1.545 | .240 |
|  | 2 | -.825 | .436 | -1.709 | .059 |
| 2 | 1 | -.510 | .668 | -1.865 | .844 |
|  | 2 | -.808 | .646 | -2.119 | .503 |

| **7. power * electrode * conflict** | | | | | | |
| --- | --- | --- | --- | --- | --- | --- |
| Measure: MEASURE_1 | | | | | | |
| power | electrode | conflict | Mean | Std. Error | 95% Confidence Interval | |
|  |  |  |  |  | Lower Bound | Upper Bound |
| 1.00 | 1 | 1 | -1.335 | .622 | -2.597 | -.073 |
|  |  | 2 | -1.683 | .616 | -2.933 | -.433 |
|  | 2 | 1 | -1.650 | .944 | -3.565 | .265 |
|  |  | 2 | -1.728 | .914 | -3.582 | .126 |
| 2.00 | 1 | 1 | .029 | .622 | -1.232 | 1.291 |
|  |  | 2 | .033 | .616 | -1.217 | 1.283 |
|  | 2 | 1 | .629 | .944 | -1.286 | 2.544 |
|  |  | 2 | .111 | .914 | -1.742 | 1.965 |

**8. power * electrode * conflict**

| **Estimates** | | | | | | |
| --- | --- | --- | --- | --- | --- | --- |
| Measure: MEASURE_1 | | | | | | |
| power | electrode | conflict | Mean | Std. Error | 95% Confidence Interval | |
|  |  |  |  |  | Lower Bound | Upper Bound |
| 1.00 | 1 | 1 | -1.335 | .622 | -2.597 | -.073 |
|  |  | 2 | -1.683 | .616 | -2.933 | -.433 |
|  | 2 | 1 | -1.650 | .944 | -3.565 | .265 |
|  |  | 2 | -1.728 | .914 | -3.582 | .126 |
| 2.00 | 1 | 1 | .029 | .622 | -1.232 | 1.291 |
|  |  | 2 | .033 | .616 | -1.217 | 1.283 |
|  | 2 | 1 | .629 | .944 | -1.286 | 2.544 |
|  |  | 2 | .111 | .914 | -1.742 | 1.965 |

| **Pairwise Comparisons** | | | | | | | | |
| --- | --- | --- | --- | --- | --- | --- | --- | --- |
| Measure: MEASURE_1 | | | | | | | | |
| power | electrode | (I) conflict | (J) conflict | Mean Difference (I-J) | Std. Error | Sig.^b^ | 95% Confidence Interval for Difference^b^ | |
|  |  |  |  |  |  |  | Lower Bound | Upper Bound |
| 1.00 | 1 | 1 | 2 | .348 | .235 | .148 | -.129 | .826 |
|  |  | 2 | 1 | -.348 | .235 | .148 | -.826 | .129 |
|  | 2 | 1 | 2 | .078 | .227 | .731 | -.381 | .538 |
|  |  | 2 | 1 | -.078 | .227 | .731 | -.538 | .381 |
| 2.00 | 1 | 1 | 2 | -.003 | .235 | .989 | -.481 | .474 |
|  |  | 2 | 1 | .003 | .235 | .989 | -.474 | .481 |
|  | 2 | 1 | 2 | .517^*^ | .227 | .029 | .057 | .977 |
|  |  | 2 | 1 | -.517^*^ | .227 | .029 | -.977 | -.057 |
| Based on estimated marginal means | | | | | | | | |
| *. The mean difference is significant at the .05 level. | | | | | | | | |
| b. Adjustment for multiple comparisons: Least Significant Difference (equivalent to no adjustments). | | | | | | | | |

| **Multivariate Tests** | | | | | | | | | | |
| --- | --- | --- | --- | --- | --- | --- | --- | --- | --- | --- |
| power | electrode | | Value | F | Hypothesis df | Error df | Sig. | Partial Eta Squared | Noncent. Parameter | Observed Power^b^ |
| 1.00 | 1 | Pillai's trace | .057 | 2.189^a^ | 1.000 | 36.000 | .148 | .057 | 2.189 | .302 |
|  |  | Wilks' lambda | .943 | 2.189^a^ | 1.000 | 36.000 | .148 | .057 | 2.189 | .302 |
|  |  | Hotelling's trace | .061 | 2.189^a^ | 1.000 | 36.000 | .148 | .057 | 2.189 | .302 |
|  |  | Roy's largest root | .061 | 2.189^a^ | 1.000 | 36.000 | .148 | .057 | 2.189 | .302 |
|  | 2 | Pillai's trace | .003 | .120^a^ | 1.000 | 36.000 | .731 | .003 | .120 | .063 |
|  |  | Wilks' lambda | .997 | .120^a^ | 1.000 | 36.000 | .731 | .003 | .120 | .063 |
|  |  | Hotelling's trace | .003 | .120^a^ | 1.000 | 36.000 | .731 | .003 | .120 | .063 |
|  |  | Roy's largest root | .003 | .120^a^ | 1.000 | 36.000 | .731 | .003 | .120 | .063 |
| 2.00 | 1 | Pillai's trace | .000 | .000^a^ | 1.000 | 36.000 | .989 | .000 | .000 | .050 |
|  |  | Wilks' lambda | 1.000 | .000^a^ | 1.000 | 36.000 | .989 | .000 | .000 | .050 |
|  |  | Hotelling's trace | .000 | .000^a^ | 1.000 | 36.000 | .989 | .000 | .000 | .050 |
|  |  | Roy's largest root | .000 | .000^a^ | 1.000 | 36.000 | .989 | .000 | .000 | .050 |
|  | 2 | Pillai's trace | .126 | 5.203^a^ | 1.000 | 36.000 | .029 | .126 | 5.203 | .603 |
|  |  | Wilks' lambda | .874 | 5.203^a^ | 1.000 | 36.000 | .029 | .126 | 5.203 | .603 |
|  |  | Hotelling's trace | .145 | 5.203^a^ | 1.000 | 36.000 | .029 | .126 | 5.203 | .603 |
|  |  | Roy's largest root | .145 | 5.203^a^ | 1.000 | 36.000 | .029 | .126 | 5.203 | .603 |
| Each F tests the multivariate simple effects of conflict within each level combination of the other effects shown. These tests are based on the linearly independent pairwise comparisons among the estimated marginal means. | | | | | | | | | | |
| a. Exact statistic | | | | | | | | | | |
| b. Computed using alpha = .05 | | | | | | | | | | |

**Only happy faces in N170**

GLM P7 congruent P7incongruent P8 congruent P8incongruent BY power

/WSFACTOR=electrode 2 Polynomial conflict 2 Polynomial

/METHOD=SSTYPE(3)

/EMMEANS=TABLES(power) COMPARE ADJ(LSD)

/EMMEANS=TABLES(electrode) COMPARE ADJ(LSD)

/EMMEANS=TABLES(conflict) COMPARE ADJ(LSD)

/EMMEANS=TABLES(power*electrode)

/EMMEANS=TABLES(power*conflict)

/EMMEANS=TABLES(electrode*conflict)

/EMMEANS=TABLES(power*electrode*conflict)

/PRINT=DESCRIPTIVE ETASQ OPOWER HOMOGENEITY

/CRITERIA=ALPHA(.05)

/WSDESIGN=electrode conflict electrode*conflict

/DESIGN=power.

**General Linear Model**

| **Notes** | | |
| --- | --- | --- |
| Output Created | | 04-DEC-2020 23:06:06 |
| Comments | |  |
| Input | Data | C:\Users\lenovo\Desktop\N170 happy.sav |
|  | Active Dataset | dataset |
|  | Filter | <none> |
|  | Weight | <none> |
|  | Split File | <none> |
|  | N of Rows in Working Data File | 41 |
| Missing Value Handling | Definition of Missing | User-defined missing values are treated as missing. |
|  | Cases Used | Statistics are based on all cases with valid data for all variables in the model. |
| Syntax | | GLM P7 congruent P7incongruent P8 congruent P8incongruent BY power  /WSFACTOR=electrode 2 Polynomial conflict 2 Polynomial  /METHOD=SSTYPE(3)  /EMMEANS=TABLES(power) COMPARE ADJ(LSD)  /EMMEANS=TABLES(electrode) COMPARE ADJ(LSD)  /EMMEANS=TABLES(conflict) COMPARE ADJ(LSD)  /EMMEANS=TABLES(power*electrode)  /EMMEANS=TABLES(power*conflict)  /EMMEANS=TABLES(electrode*conflict)  /EMMEANS=TABLES(power*electrode*conflict)  /PRINT=DESCRIPTIVE ETASQ OPOWER HOMOGENEITY  /CRITERIA=ALPHA(.05)  /WSDESIGN=electrode conflict electrode*conflict  /DESIGN=power. |
| Resources | Processor Time | 00:00:00.05 |
|  | Elapsed Time | 00:00:00.04 |

| **Within-Subjects Factors** | | |
| --- | --- | --- |
| Measure: MEASURE_1 | | |
| electrode | conflict | Dependent Variable |
| 1 | 1 | P7congruent |
|  | 2 | P7incongruent |
| 2 | 1 | P8congruent |
|  | 2 | P8incongruent |

| **Between-Subjects Factors** | | |
| --- | --- | --- |
|  | | N |
| power | 1.00 | 19 |
|  | 2.00 | 19 |

| **Descriptive Statistics** | | | | |
| --- | --- | --- | --- | --- |
|  | power | Mean | Std. Deviation | N |
| P7congruent | 1.00 | -4.4784 | 4.88787 | 19 |
|  | 2.00 | -2.3939 | 2.45649 | 19 |
|  | Total | -3.4361 | 3.95905 | 38 |
| P7incongruent | 1.00 | -4.9372 | 4.72755 | 19 |
|  | 2.00 | -2.4409 | 2.58984 | 19 |
|  | Total | -3.6891 | 3.96685 | 38 |
| P8congruent | 1.00 | -6.2620 | 6.57442 | 19 |
|  | 2.00 | -3.5120 | 4.35820 | 19 |
|  | Total | -4.8870 | 5.67534 | 38 |
| P8incongruent | 1.00 | -6.2529 | 6.20181 | 19 |
|  | 2.00 | -3.7711 | 4.71167 | 19 |
|  | Total | -5.0120 | 5.57610 | 38 |

| **Box's Test of Equality of Covariance Matrices^a^** | |
| --- | --- |
| Box's M | 27.181 |
| F | 2.389 |
| df1 | 10 |
| df2 | 6196.016 |
| Sig. | .008 |
| Tests the null hypothesis that the observed covariance matrices of the dependent variables are equal across groups. | |
| a. Design: + power  Within Subjects Design: electrode + conflict + electrode * conflict | |

| **Multivariate Tests^a^** | | | | | | | | | |
| --- | --- | --- | --- | --- | --- | --- | --- | --- | --- |
| Effect | | Value | F | Hypothesis df | Error df | Sig. | Partial Eta Squared | Noncent. Parameter | Observed Power^c^ |
| electrode | Pillai's Trace | .107 | 4.294^b^ | 1.000 | 36.000 | .045 | .107 | 4.294 | .523 |
|  | Wilks' Lambda | .893 | 4.294^b^ | 1.000 | 36.000 | .045 | .107 | 4.294 | .523 |
|  | Hotelling's Trace | .119 | 4.294^b^ | 1.000 | 36.000 | .045 | .107 | 4.294 | .523 |
|  | Roy's Largest Root | .119 | 4.294^b^ | 1.000 | 36.000 | .045 | .107 | 4.294 | .523 |
| electrode * power | Pillai's Trace | .002 | .059^b^ | 1.000 | 36.000 | .809 | .002 | .059 | .056 |
|  | Wilks' Lambda | .998 | .059^b^ | 1.000 | 36.000 | .809 | .002 | .059 | .056 |
|  | Hotelling's Trace | .002 | .059^b^ | 1.000 | 36.000 | .809 | .002 | .059 | .056 |
|  | Roy's Largest Root | .002 | .059^b^ | 1.000 | 36.000 | .809 | .002 | .059 | .056 |
| conflict | Pillai's Trace | .043 | 1.613^b^ | 1.000 | 36.000 | .212 | .043 | 1.613 | .235 |
|  | Wilks' Lambda | .957 | 1.613^b^ | 1.000 | 36.000 | .212 | .043 | 1.613 | .235 |
|  | Hotelling's Trace | .045 | 1.613^b^ | 1.000 | 36.000 | .212 | .043 | 1.613 | .235 |
|  | Roy's Largest Root | .045 | 1.613^b^ | 1.000 | 36.000 | .212 | .043 | 1.613 | .235 |
| conflict * power | Pillai's Trace | .002 | .058^b^ | 1.000 | 36.000 | .811 | .002 | .058 | .056 |
|  | Wilks' Lambda | .998 | .058^b^ | 1.000 | 36.000 | .811 | .002 | .058 | .056 |
|  | Hotelling's Trace | .002 | .058^b^ | 1.000 | 36.000 | .811 | .002 | .058 | .056 |
|  | Roy's Largest Root | .002 | .058^b^ | 1.000 | 36.000 | .811 | .002 | .058 | .056 |
| electrode * conflict | Pillai's Trace | .010 | .350^b^ | 1.000 | 36.000 | .558 | .010 | .350 | .089 |
|  | Wilks' Lambda | .990 | .350^b^ | 1.000 | 36.000 | .558 | .010 | .350 | .089 |
|  | Hotelling's Trace | .010 | .350^b^ | 1.000 | 36.000 | .558 | .010 | .350 | .089 |
|  | Roy's Largest Root | .010 | .350^b^ | 1.000 | 36.000 | .558 | .010 | .350 | .089 |
| electrode * conflict * power | Pillai's Trace | .064 | 2.470^b^ | 1.000 | 36.000 | .125 | .064 | 2.470 | .334 |
|  | Wilks' Lambda | .936 | 2.470^b^ | 1.000 | 36.000 | .125 | .064 | 2.470 | .334 |
|  | Hotelling's Trace | .069 | 2.470^b^ | 1.000 | 36.000 | .125 | .064 | 2.470 | .334 |
|  | Roy's Largest Root | .069 | 2.470^b^ | 1.000 | 36.000 | .125 | .064 | 2.470 | .334 |
| a. Design: + power  Within Subjects Design: electrode + conflict + electrode * conflict | | | | | | | | | |
| b. Exact statistic | | | | | | | | | |
| c. Computed using alpha = .05 | | | | | | | | | |

| **Mauchly's Test of Sphericity^a^** | | | | | | | |
| --- | --- | --- | --- | --- | --- | --- | --- |
| Measure: MEASURE_1 | | | | | | | |
| Within Subjects Effect | Mauchly's W | Approx. Chi-Square | df | Sig. | Epsilon^b^ | | |
|  |  |  |  |  | Greenhouse-Geisser | Huynh-Feldt | Lower-bound |
| electrode | 1.000 | .000 | 0 | . | 1.000 | 1.000 | 1.000 |
| conflict | 1.000 | .000 | 0 | . | 1.000 | 1.000 | 1.000 |
| electrode * conflict | 1.000 | .000 | 0 | . | 1.000 | 1.000 | 1.000 |
| Tests the null hypothesis that the error covariance matrix of the orthonormalized transformed dependent variables is proportional to an identity matrix. | | | | | | | |
| a. Design: + power  Within Subjects Design: electrode + conflict + electrode * conflict | | | | | | | |
| b. May be used to adjust the degrees of freedom for the averaged tests of significance. Corrected tests are displayed in the Tests of Within-Subjects Effects table. | | | | | | | |

| **Tests of Within-Subjects Effects** | | | | | | | | | |
| --- | --- | --- | --- | --- | --- | --- | --- | --- | --- |
| Measure: MEASURE_1 | | | | | | | | | |
| Source | | Type III Sum of Squares | df | Mean Square | F | Sig. | Partial Eta Squared | Noncent. Parameter | Observed Power^a^ |
| electrode | Sphericity Assumed | 73.090 | 1 | 73.090 | 4.294 | .045 | .107 | 4.294 | .523 |
|  | Greenhouse-Geisser | 73.090 | 1.000 | 73.090 | 4.294 | .045 | .107 | 4.294 | .523 |
|  | Huynh-Feldt | 73.090 | 1.000 | 73.090 | 4.294 | .045 | .107 | 4.294 | .523 |
|  | Lower-bound | 73.090 | 1.000 | 73.090 | 4.294 | .045 | .107 | 4.294 | .523 |
| electrode * power | Sphericity Assumed | 1.006 | 1 | 1.006 | .059 | .809 | .002 | .059 | .056 |
|  | Greenhouse-Geisser | 1.006 | 1.000 | 1.006 | .059 | .809 | .002 | .059 | .056 |
|  | Huynh-Feldt | 1.006 | 1.000 | 1.006 | .059 | .809 | .002 | .059 | .056 |
|  | Lower-bound | 1.006 | 1.000 | 1.006 | .059 | .809 | .002 | .059 | .056 |
| Error(electrode) | Sphericity Assumed | 612.844 | 36 | 17.023 |  |  |  |  |  |
|  | Greenhouse-Geisser | 612.844 | 36.000 | 17.023 |  |  |  |  |  |
|  | Huynh-Feldt | 612.844 | 36.000 | 17.023 |  |  |  |  |  |
|  | Lower-bound | 612.844 | 36.000 | 17.023 |  |  |  |  |  |
| conflict | Sphericity Assumed | 1.357 | 1 | 1.357 | 1.613 | .212 | .043 | 1.613 | .235 |
|  | Greenhouse-Geisser | 1.357 | 1.000 | 1.357 | 1.613 | .212 | .043 | 1.613 | .235 |
|  | Huynh-Feldt | 1.357 | 1.000 | 1.357 | 1.613 | .212 | .043 | 1.613 | .235 |
|  | Lower-bound | 1.357 | 1.000 | 1.357 | 1.613 | .212 | .043 | 1.613 | .235 |
| conflict * power | Sphericity Assumed | .049 | 1 | .049 | .058 | .811 | .002 | .058 | .056 |
|  | Greenhouse-Geisser | .049 | 1.000 | .049 | .058 | .811 | .002 | .058 | .056 |
|  | Huynh-Feldt | .049 | 1.000 | .049 | .058 | .811 | .002 | .058 | .056 |
|  | Lower-bound | .049 | 1.000 | .049 | .058 | .811 | .002 | .058 | .056 |
| Error(conflict) | Sphericity Assumed | 30.289 | 36 | .841 |  |  |  |  |  |
|  | Greenhouse-Geisser | 30.289 | 36.000 | .841 |  |  |  |  |  |
|  | Huynh-Feldt | 30.289 | 36.000 | .841 |  |  |  |  |  |
|  | Lower-bound | 30.289 | 36.000 | .841 |  |  |  |  |  |
| electrode * conflict | Sphericity Assumed | .155 | 1 | .155 | .350 | .558 | .010 | .350 | .089 |
|  | Greenhouse-Geisser | .155 | 1.000 | .155 | .350 | .558 | .010 | .350 | .089 |
|  | Huynh-Feldt | .155 | 1.000 | .155 | .350 | .558 | .010 | .350 | .089 |
|  | Lower-bound | .155 | 1.000 | .155 | .350 | .558 | .010 | .350 | .089 |
| electrode * conflict * power | Sphericity Assumed | 1.098 | 1 | 1.098 | 2.470 | .125 | .064 | 2.470 | .334 |
|  | Greenhouse-Geisser | 1.098 | 1.000 | 1.098 | 2.470 | .125 | .064 | 2.470 | .334 |
|  | Huynh-Feldt | 1.098 | 1.000 | 1.098 | 2.470 | .125 | .064 | 2.470 | .334 |
|  | Lower-bound | 1.098 | 1.000 | 1.098 | 2.470 | .125 | .064 | 2.470 | .334 |
| Error(electrode*conflict) | Sphericity Assumed | 16.008 | 36 | .445 |  |  |  |  |  |
|  | Greenhouse-Geisser | 16.008 | 36.000 | .445 |  |  |  |  |  |
|  | Huynh-Feldt | 16.008 | 36.000 | .445 |  |  |  |  |  |
|  | Lower-bound | 16.008 | 36.000 | .445 |  |  |  |  |  |
| a. Computed using alpha = .05 | | | | | | | | | |

| **Tests of Within-Subjects Contrasts** | | | | | | | | | | |
| --- | --- | --- | --- | --- | --- | --- | --- | --- | --- | --- |
| Measure: MEASURE_1 | | | | | | | | | | |
| Source | electrode | conflict | Type III Sum of Squares | df | Mean Square | F | Sig. | Partial Eta Squared | Noncent. Parameter | Observed Power^a^ |
| electrode | Linear |  | 73.090 | 1 | 73.090 | 4.294 | .045 | .107 | 4.294 | .523 |
| electrode * power | Linear |  | 1.006 | 1 | 1.006 | .059 | .809 | .002 | .059 | .056 |
| Error(electrode) | Linear |  | 612.844 | 36 | 17.023 |  |  |  |  |  |
| conflict |  | Linear | 1.357 | 1 | 1.357 | 1.613 | .212 | .043 | 1.613 | .235 |
| conflict * power |  | Linear | .049 | 1 | .049 | .058 | .811 | .002 | .058 | .056 |
| Error(conflict) |  | Linear | 30.289 | 36 | .841 |  |  |  |  |  |
| electrode * conflict | Linear | Linear | .155 | 1 | .155 | .350 | .558 | .010 | .350 | .089 |
| electrode * conflict * power | Linear | Linear | 1.098 | 1 | 1.098 | 2.470 | .125 | .064 | 2.470 | .334 |
| Error(electrode*conflict) | Linear | Linear | 16.008 | 36 | .445 |  |  |  |  |  |
| a. Computed using alpha = .05 | | | | | | | | | | |

| **Levene's Test of Equality of Error Variances^a^** | | | | |
| --- | --- | --- | --- | --- |
|  | F | df1 | df2 | Sig. |
| P7congruent | 8.948 | 1 | 36 | .005 |
| P7incongruent | 6.959 | 1 | 36 | .012 |
| P8congruent | 3.060 | 1 | 36 | .089 |
| P8incongruent | 1.397 | 1 | 36 | .245 |
| Tests the null hypothesis that the error variance of the dependent variable is equal across groups. | | | | |
| a. Design: + power  Within Subjects Design: electrode + conflict + electrode * conflict | | | | |

| **Tests of Between-Subjects Effects** | | | | | | | | |
| --- | --- | --- | --- | --- | --- | --- | --- | --- |
| Measure: MEASURE_1 | | | | | | | | |
| Transformed Variable: Average | | | | | | | | |
| Source | Type III Sum of Squares | df | Mean Square | F | Sig. | Partial Eta Squared | Noncent. Parameter | Observed Power^a^ |
|  | 2753.318 | 1 | 2753.318 | 37.913 | .000 | .513 | 37.913 | 1.000 |
| power | 228.687 | 1 | 228.687 | 3.149 | .084 | .080 | 3.149 | .408 |
| Error | 2614.372 | 36 | 72.621 |  |  |  |  |  |
| a. Computed using alpha = .05 | | | | | | | | |

**Estimated Marginal Means**

**1. power**

| **Estimates** | | | | |
| --- | --- | --- | --- | --- |
| Measure: MEASURE_1 | | | | |
| power | Mean | Std. Error | 95% Confidence Interval | |
|  |  |  | Lower Bound | Upper Bound |
| 1.00 | -5.483 | .978 | -7.465 | -3.500 |
| 2.00 | -3.029 | .978 | -5.012 | -1.047 |

| **Pairwise Comparisons** | | | | | | |
| --- | --- | --- | --- | --- | --- | --- |
| Measure: MEASURE_1 | | | | | | |
| (I) power | (J) power | Mean Difference (I-J) | Std. Error | Sig.^a^ | 95% Confidence Interval for Difference^a^ | |
|  |  |  |  |  | Lower Bound | Upper Bound |
| 1.00 | 2.00 | -2.453 | 1.382 | .084 | -5.257 | .351 |
| 2.00 | 1.00 | 2.453 | 1.382 | .084 | -.351 | 5.257 |
| Based on estimated marginal means | | | | | | |
| a. Adjustment for multiple comparisons: Least Significant Difference (equivalent to no adjustments). | | | | | | |

| **Univariate Tests** | | | | | | | | |
| --- | --- | --- | --- | --- | --- | --- | --- | --- |
| Measure: MEASURE_1 | | | | | | | | |
|  | Sum of Squares | df | Mean Square | F | Sig. | Partial Eta Squared | Noncent. Parameter | Observed Power^a^ |
| Contrast | 57.172 | 1 | 57.172 | 3.149 | .084 | .080 | 3.149 | .408 |
| Error | 653.593 | 36 | 18.155 |  |  |  |  |  |
| The F tests the effect of power. This test is based on the linearly independent pairwise comparisons among the estimated marginal means. | | | | | | | | |
| a. Computed using alpha = .05 | | | | | | | | |

**2. electrode**

| **Estimates** | | | | |
| --- | --- | --- | --- | --- |
| Measure: MEASURE_1 | | | | |
| electrode | Mean | Std. Error | 95% Confidence Interval | |
|  |  |  | Lower Bound | Upper Bound |
| 1 | -3.563 | .617 | -4.813 | -2.312 |
| 2 | -4.949 | .894 | -6.763 | -3.136 |

| **Pairwise Comparisons** | | | | | | |
| --- | --- | --- | --- | --- | --- | --- |
| Measure: MEASURE_1 | | | | | | |
| (I) electrode | (J) electrode | Mean Difference (I-J) | Std. Error | Sig.^b^ | 95% Confidence Interval for Difference^b^ | |
|  |  |  |  |  | Lower Bound | Upper Bound |
| 1 | 2 | 1.387^*^ | .669 | .045 | .029 | 2.744 |
| 2 | 1 | -1.387^*^ | .669 | .045 | -2.744 | -.029 |
| Based on estimated marginal means | | | | | | |
| *. The mean difference is significant at the .05 level. | | | | | | |
| b. Adjustment for multiple comparisons: Least Significant Difference (equivalent to no adjustments). | | | | | | |

| **Multivariate Tests** | | | | | | | | |
| --- | --- | --- | --- | --- | --- | --- | --- | --- |
|  | Value | F | Hypothesis df | Error df | Sig. | Partial Eta Squared | Noncent. Parameter | Observed Power^b^ |
| Pillai's trace | .107 | 4.294^a^ | 1.000 | 36.000 | .045 | .107 | 4.294 | .523 |
| Wilks' lambda | .893 | 4.294^a^ | 1.000 | 36.000 | .045 | .107 | 4.294 | .523 |
| Hotelling's trace | .119 | 4.294^a^ | 1.000 | 36.000 | .045 | .107 | 4.294 | .523 |
| Roy's largest root | .119 | 4.294^a^ | 1.000 | 36.000 | .045 | .107 | 4.294 | .523 |
| Each F tests the multivariate effect of electrode. These tests are based on the linearly independent pairwise comparisons among the estimated marginal means. | | | | | | | | |
| a. Exact statistic | | | | | | | | |
| b. Computed using alpha = .05 | | | | | | | | |

**3. conflict**

| **Estimates** | | | | |
| --- | --- | --- | --- | --- |
| Measure: MEASURE_1 | | | | |
| conflict | Mean | Std. Error | 95% Confidence Interval | |
|  |  |  | Lower Bound | Upper Bound |
| 1 | -4.162 | .703 | -5.587 | -2.736 |
| 2 | -4.351 | .687 | -5.745 | -2.956 |

| **Pairwise Comparisons** | | | | | | |
| --- | --- | --- | --- | --- | --- | --- |
| Measure: MEASURE_1 | | | | | | |
| (I) conflict | (J) conflict | Mean Difference (I-J) | Std. Error | Sig.^a^ | 95% Confidence Interval for Difference^a^ | |
|  |  |  |  |  | Lower Bound | Upper Bound |
| 1 | 2 | .189 | .149 | .212 | -.113 | .491 |
| 2 | 1 | -.189 | .149 | .212 | -.491 | .113 |
| Based on estimated marginal means | | | | | | |
| a. Adjustment for multiple comparisons: Least Significant Difference (equivalent to no adjustments). | | | | | | |

| **Multivariate Tests** | | | | | | | | |
| --- | --- | --- | --- | --- | --- | --- | --- | --- |
|  | Value | F | Hypothesis df | Error df | Sig. | Partial Eta Squared | Noncent. Parameter | Observed Power^b^ |
| Pillai's trace | .043 | 1.613^a^ | 1.000 | 36.000 | .212 | .043 | 1.613 | .235 |
| Wilks' lambda | .957 | 1.613^a^ | 1.000 | 36.000 | .212 | .043 | 1.613 | .235 |
| Hotelling's trace | .045 | 1.613^a^ | 1.000 | 36.000 | .212 | .043 | 1.613 | .235 |
| Roy's largest root | .045 | 1.613^a^ | 1.000 | 36.000 | .212 | .043 | 1.613 | .235 |
| Each F tests the multivariate effect of conflict. These tests are based on the linearly independent pairwise comparisons among the estimated marginal means. | | | | | | | | |
| a. Exact statistic | | | | | | | | |
| b. Computed using alpha = .05 | | | | | | | | |

| **4. power * electrode** | | | | | |
| --- | --- | --- | --- | --- | --- |
| Measure: MEASURE_1 | | | | | |
| power | electrode | Mean | Std. Error | 95% Confidence Interval | |
|  |  |  |  | Lower Bound | Upper Bound |
| 1.00 | 1 | -4.708 | .872 | -6.476 | -2.939 |
|  | 2 | -6.257 | 1.264 | -8.822 | -3.693 |
| 2.00 | 1 | -2.417 | .872 | -4.186 | -.649 |
|  | 2 | -3.642 | 1.264 | -6.206 | -1.077 |

| **5. power * conflict** | | | | | |
| --- | --- | --- | --- | --- | --- |
| Measure: MEASURE_1 | | | | | |
| power | conflict | Mean | Std. Error | 95% Confidence Interval | |
|  |  |  |  | Lower Bound | Upper Bound |
| 1.00 | 1 | -5.370 | .994 | -7.386 | -3.354 |
|  | 2 | -5.595 | .972 | -7.567 | -3.624 |
| 2.00 | 1 | -2.953 | .994 | -4.969 | -.937 |
|  | 2 | -3.106 | .972 | -5.077 | -1.135 |

| **6. electrode * conflict** | | | | | |
| --- | --- | --- | --- | --- | --- |
| Measure: MEASURE_1 | | | | | |
| electrode | conflict | Mean | Std. Error | 95% Confidence Interval | |
|  |  |  |  | Lower Bound | Upper Bound |
| 1 | 1 | -3.436 | .628 | -4.709 | -2.164 |
|  | 2 | -3.689 | .618 | -4.943 | -2.435 |
| 2 | 1 | -4.887 | .905 | -6.722 | -3.052 |
|  | 2 | -5.012 | .893 | -6.824 | -3.200 |

| **7. power * electrode * conflict** | | | | | | |
| --- | --- | --- | --- | --- | --- | --- |
| Measure: MEASURE_1 | | | | | | |
| power | electrode | conflict | Mean | Std. Error | 95% Confidence Interval | |
|  |  |  |  |  | Lower Bound | Upper Bound |
| 1.00 | 1 | 1 | -4.478 | .887 | -6.278 | -2.679 |
|  |  | 2 | -4.937 | .874 | -6.711 | -3.164 |
|  | 2 | 1 | -6.262 | 1.280 | -8.857 | -3.667 |
|  |  | 2 | -6.253 | 1.263 | -8.815 | -3.690 |
| 2.00 | 1 | 1 | -2.394 | .887 | -4.194 | -.594 |
|  |  | 2 | -2.441 | .874 | -4.214 | -.667 |
|  | 2 | 1 | -3.512 | 1.280 | -6.107 | -.917 |
|  |  | 2 | -3.771 | 1.263 | -6.334 | -1.209 |

**Only happy faces in N450**

GLM F3 congruent F3incongruent F4 congruent F4incongruent FZ congruent FZ incongruent C3 congruent C3incongruent C4 congruent C4incongruent CZ congruent CZ incongruent CP1 congruent CP1incongruent CP2 congruent CP2incongruent BY power

/WSFACTOR=electrode 8 Polynomial conflict 2 Polynomial

/METHOD=SSTYPE(3)

/EMMEANS=TABLES(power) COMPARE ADJ(LSD)

/EMMEANS=TABLES(electrode) COMPARE ADJ(LSD)

/EMMEANS=TABLES(conflict) COMPARE ADJ(LSD)

/EMMEANS=TABLES(power*electrode)

/EMMEANS=TABLES(power*conflict)

/EMMEANS=TABLES(electrode*conflict)

/EMMEANS=TABLES(power*electrode*conflict)

/PRINT=DESCRIPTIVE ETASQ OPOWER HOMOGENEITY

/CRITERIA=ALPHA(.05)

/WSDESIGN=electrode conflict electrode*conflict

/DESIGN=power.

**General Linear Model**

| **Notes** | | |
| --- | --- | --- |
| Output Created | | 04-DEC-2020 23:34:00 |
| Comments | |  |
| Input | Data | C:\Users\lenovo\Desktop\N450 happy.sav |
|  | Active Dataset | dataset |
|  | Filter | <none> |
|  | Weight | <none> |
|  | Split File | <none> |
|  | N of Rows in Working Data File | 78 |
| Missing Value Handling | Definition of Missing | User-defined missing values are treated as missing. |
|  | Cases Used | Statistics are based on all cases with valid data for all variables in the model. |
| Syntax | | GLM F3 congruent F3incongruent F4 congruent F4incongruent FZ congruent FZ incongruent C3 congruent C3incongruent C4 congruent C4incongruent CZ congruent CZ incongruent CP1 congruent CP1incongruent CP2 congruent CP2incongruent BY power  /WSFACTOR=electrode 8 Polynomial conflict 2 Polynomial  /METHOD=SSTYPE(3)  /EMMEANS=TABLES(power) COMPARE ADJ(LSD)  /EMMEANS=TABLES(electrode) COMPARE ADJ(LSD)  /EMMEANS=TABLES(conflict) COMPARE ADJ(LSD)  /EMMEANS=TABLES(power*electrode)  /EMMEANS=TABLES(power*conflict)  /EMMEANS=TABLES(electrode*conflict)  /EMMEANS=TABLES(power*electrode*conflict)  /PRINT=DESCRIPTIVE ETASQ OPOWER HOMOGENEITY  /CRITERIA=ALPHA(.05)  /WSDESIGN=electrode conflict electrode*conflict  /DESIGN=power. |
| Resources | Processor Time | 00:00:00.11 |
|  | Elapsed Time | 00:00:00.07 |

| **Within-Subjects Factors** | | |
| --- | --- | --- |
| Measure: MEASURE_1 | | |
| electrode | conflict | Dependent Variable |
| 1 | 1 | F3congruent |
|  | 2 | F3incongruent |
| 2 | 1 | F4congruent |
|  | 2 | F4incongruent |
| 3 | 1 | FZ congruent |
|  | 2 | FZ incongruent |
| 4 | 1 | C3congruent |
|  | 2 | C3incongruent |
| 5 | 1 | C4congruent |
|  | 2 | C4incongruent |
| 6 | 1 | CZ congruent |
|  | 2 | CZ incongruent |
| 7 | 1 | CP1congruent |
|  | 2 | CP1incongruent |
| 8 | 1 | CP2congruent |
|  | 2 | CP2incongruent |

| **Between-Subjects Factors** | | |
| --- | --- | --- |
|  | | N |
| power | 1.00 | 19 |
|  | 2.00 | 19 |

| **Descriptive Statistics** | | | | |
| --- | --- | --- | --- | --- |
|  | power | Mean | Std. Deviation | N |
| F3congruent | 1.00 | .8861 | 4.65271 | 19 |
|  | 2.00 | 1.8942 | 3.06081 | 19 |
|  | Total | 1.3901 | 3.91789 | 38 |
| F3incongruent | 1.00 | 1.1237 | 4.72325 | 19 |
|  | 2.00 | 1.2758 | 2.92808 | 19 |
|  | Total | 1.1998 | 3.87685 | 38 |
| F4congruent | 1.00 | 2.7060 | 5.55808 | 19 |
|  | 2.00 | 2.3390 | 2.91934 | 19 |
|  | Total | 2.5225 | 4.38285 | 38 |
| F4incongruent | 1.00 | 2.2033 | 5.52660 | 19 |
|  | 2.00 | 1.7296 | 2.84638 | 19 |
|  | Total | 1.9665 | 4.34257 | 38 |
| FZ congruent | 1.00 | 1.7603 | 5.55318 | 19 |
|  | 2.00 | 1.7350 | 3.01489 | 19 |
|  | Total | 1.7477 | 4.40730 | 38 |
| FZ incongruent | 1.00 | 1.5311 | 5.58831 | 19 |
|  | 2.00 | 1.3570 | 2.85195 | 19 |
|  | Total | 1.4441 | 4.37690 | 38 |
| C3congruent | 1.00 | 2.9622 | 2.47176 | 19 |
|  | 2.00 | 3.5275 | 2.92801 | 19 |
|  | Total | 3.2449 | 2.68794 | 38 |
| C3incongruent | 1.00 | 2.8282 | 2.74239 | 19 |
|  | 2.00 | 2.8476 | 2.65594 | 19 |
|  | Total | 2.8379 | 2.66280 | 38 |
| C4congruent | 1.00 | 5.1197 | 3.65678 | 19 |
|  | 2.00 | 4.7123 | 2.30126 | 19 |
|  | Total | 4.9160 | 3.02064 | 38 |
| C4incongruent | 1.00 | 4.5006 | 3.90764 | 19 |
|  | 2.00 | 4.0018 | 2.61291 | 19 |
|  | Total | 4.2512 | 3.28843 | 38 |
| CZ congruent | 1.00 | 4.2400 | 3.64553 | 19 |
|  | 2.00 | 4.2130 | 3.01957 | 19 |
|  | Total | 4.2265 | 3.30171 | 38 |
| CZ incongruent | 1.00 | 3.9657 | 3.65265 | 19 |
|  | 2.00 | 3.8817 | 3.84468 | 19 |
|  | Total | 3.9237 | 3.69912 | 38 |
| CP1congruent | 1.00 | 5.6698 | 3.05679 | 19 |
|  | 2.00 | 6.1871 | 2.83120 | 19 |
|  | Total | 5.9285 | 2.91787 | 38 |
| CP1incongruent | 1.00 | 5.6061 | 3.23957 | 19 |
|  | 2.00 | 5.6714 | 3.56880 | 19 |
|  | Total | 5.6387 | 3.36195 | 38 |
| CP2congruent | 1.00 | 6.8733 | 3.72285 | 19 |
|  | 2.00 | 6.7740 | 2.77210 | 19 |
|  | Total | 6.8236 | 3.23782 | 38 |
| CP2incongruent | 1.00 | 6.6790 | 4.08183 | 19 |
|  | 2.00 | 6.2396 | 3.41378 | 19 |
|  | Total | 6.4593 | 3.71814 | 38 |

| **Box's Test of Equality of Covariance Matrices^a^** | |
| --- | --- |
| Box's M | 364.403 |
| F | 1.365 |
| df1 | 136 |
| df2 | 4002.174 |
| Sig. | .004 |
| Tests the null hypothesis that the observed covariance matrices of the dependent variables are equal across groups. | |
| a. Design: + power  Within Subjects Design: electrode + conflict + electrode * conflict | |

| **Multivariate Tests^a^** | | | | | | | | | |
| --- | --- | --- | --- | --- | --- | --- | --- | --- | --- |
| Effect | | Value | F | Hypothesis df | Error df | Sig. | Partial Eta Squared | Noncent. Parameter | Observed Power^c^ |
| electrode | Pillai's Trace | .776 | 14.861^b^ | 7.000 | 30.000 | .000 | .776 | 104.025 | 1.000 |
|  | Wilks' Lambda | .224 | 14.861^b^ | 7.000 | 30.000 | .000 | .776 | 104.025 | 1.000 |
|  | Hotelling's Trace | 3.468 | 14.861^b^ | 7.000 | 30.000 | .000 | .776 | 104.025 | 1.000 |
|  | Roy's Largest Root | 3.468 | 14.861^b^ | 7.000 | 30.000 | .000 | .776 | 104.025 | 1.000 |
| electrode * power | Pillai's Trace | .064 | .292^b^ | 7.000 | 30.000 | .952 | .064 | 2.043 | .120 |
|  | Wilks' Lambda | .936 | .292^b^ | 7.000 | 30.000 | .952 | .064 | 2.043 | .120 |
|  | Hotelling's Trace | .068 | .292^b^ | 7.000 | 30.000 | .952 | .064 | 2.043 | .120 |
|  | Roy's Largest Root | .068 | .292^b^ | 7.000 | 30.000 | .952 | .064 | 2.043 | .120 |
| conflict | Pillai's Trace | .060 | 2.307^b^ | 1.000 | 36.000 | .138 | .060 | 2.307 | .315 |
|  | Wilks' Lambda | .940 | 2.307^b^ | 1.000 | 36.000 | .138 | .060 | 2.307 | .315 |
|  | Hotelling's Trace | .064 | 2.307^b^ | 1.000 | 36.000 | .138 | .060 | 2.307 | .315 |
|  | Roy's Largest Root | .064 | 2.307^b^ | 1.000 | 36.000 | .138 | .060 | 2.307 | .315 |
| conflict * power | Pillai's Trace | .011 | .411^b^ | 1.000 | 36.000 | .526 | .011 | .411 | .096 |
|  | Wilks' Lambda | .989 | .411^b^ | 1.000 | 36.000 | .526 | .011 | .411 | .096 |
|  | Hotelling's Trace | .011 | .411^b^ | 1.000 | 36.000 | .526 | .011 | .411 | .096 |
|  | Roy's Largest Root | .011 | .411^b^ | 1.000 | 36.000 | .526 | .011 | .411 | .096 |
| electrode * conflict | Pillai's Trace | .185 | .973^b^ | 7.000 | 30.000 | .469 | .185 | 6.808 | .344 |
|  | Wilks' Lambda | .815 | .973^b^ | 7.000 | 30.000 | .469 | .185 | 6.808 | .344 |
|  | Hotelling's Trace | .227 | .973^b^ | 7.000 | 30.000 | .469 | .185 | 6.808 | .344 |
|  | Roy's Largest Root | .227 | .973^b^ | 7.000 | 30.000 | .469 | .185 | 6.808 | .344 |
| electrode * conflict * power | Pillai's Trace | .221 | 1.213^b^ | 7.000 | 30.000 | .326 | .221 | 8.491 | .429 |
|  | Wilks' Lambda | .779 | 1.213^b^ | 7.000 | 30.000 | .326 | .221 | 8.491 | .429 |
|  | Hotelling's Trace | .283 | 1.213^b^ | 7.000 | 30.000 | .326 | .221 | 8.491 | .429 |
|  | Roy's Largest Root | .283 | 1.213^b^ | 7.000 | 30.000 | .326 | .221 | 8.491 | .429 |
| a. Design: + power  Within Subjects Design: electrode + conflict + electrode * conflict | | | | | | | | | |
| b. Exact statistic | | | | | | | | | |
| c. Computed using alpha = .05 | | | | | | | | | |

| **Mauchly's Test of Sphericity^a^** | | | | | | | |
| --- | --- | --- | --- | --- | --- | --- | --- |
| Measure: MEASURE_1 | | | | | | | |
| Within Subjects Effect | Mauchly's W | Approx. Chi-Square | df | Sig. | Epsilon^b^ | | |
|  |  |  |  |  | Greenhouse-Geisser | Huynh-Feldt | Lower-bound |
| electrode | .000 | 265.316 | 27 | .000 | .297 | .324 | .143 |
| conflict | 1.000 | .000 | 0 | . | 1.000 | 1.000 | 1.000 |
| electrode * conflict | .044 | 104.336 | 27 | .000 | .534 | .620 | .143 |
| Tests the null hypothesis that the error covariance matrix of the orthonormalized transformed dependent variables is proportional to an identity matrix. | | | | | | | |
| a. Design: + power  Within Subjects Design: electrode + conflict + electrode * conflict | | | | | | | |
| b. May be used to adjust the degrees of freedom for the averaged tests of significance. Corrected tests are displayed in the Tests of Within-Subjects Effects table. | | | | | | | |

| **Tests of Within-Subjects Effects** | | | | | | | | | |
| --- | --- | --- | --- | --- | --- | --- | --- | --- | --- |
| Measure: MEASURE_1 | | | | | | | | | |
| Source | | Type III Sum of Squares | df | Mean Square | F | Sig. | Partial Eta Squared | Noncent. Parameter | Observed Power^a^ |
| electrode | Sphericity Assumed | 2026.508 | 7 | 289.501 | 28.343 | .000 | .440 | 198.399 | 1.000 |
|  | Greenhouse-Geisser | 2026.508 | 2.076 | 976.196 | 28.343 | .000 | .440 | 58.837 | 1.000 |
|  | Huynh-Feldt | 2026.508 | 2.266 | 894.158 | 28.343 | .000 | .440 | 64.236 | 1.000 |
|  | Lower-bound | 2026.508 | 1.000 | 2026.508 | 28.343 | .000 | .440 | 28.343 | .999 |
| electrode * power | Sphericity Assumed | 18.469 | 7 | 2.638 | .258 | .969 | .007 | 1.808 | .126 |
|  | Greenhouse-Geisser | 18.469 | 2.076 | 8.897 | .258 | .781 | .007 | .536 | .090 |
|  | Huynh-Feldt | 18.469 | 2.266 | 8.149 | .258 | .800 | .007 | .585 | .092 |
|  | Lower-bound | 18.469 | 1.000 | 18.469 | .258 | .614 | .007 | .258 | .078 |
| Error(electrode) | Sphericity Assumed | 2574.003 | 252 | 10.214 |  |  |  |  |  |
|  | Greenhouse-Geisser | 2574.003 | 74.733 | 34.443 |  |  |  |  |  |
|  | Huynh-Feldt | 2574.003 | 81.590 | 31.548 |  |  |  |  |  |
|  | Lower-bound | 2574.003 | 36.000 | 71.500 |  |  |  |  |  |
| conflict | Sphericity Assumed | 22.511 | 1 | 22.511 | 2.307 | .138 | .060 | 2.307 | .315 |
|  | Greenhouse-Geisser | 22.511 | 1.000 | 22.511 | 2.307 | .138 | .060 | 2.307 | .315 |
|  | Huynh-Feldt | 22.511 | 1.000 | 22.511 | 2.307 | .138 | .060 | 2.307 | .315 |
|  | Lower-bound | 22.511 | 1.000 | 22.511 | 2.307 | .138 | .060 | 2.307 | .315 |
| conflict * power | Sphericity Assumed | 4.007 | 1 | 4.007 | .411 | .526 | .011 | .411 | .096 |
|  | Greenhouse-Geisser | 4.007 | 1.000 | 4.007 | .411 | .526 | .011 | .411 | .096 |
|  | Huynh-Feldt | 4.007 | 1.000 | 4.007 | .411 | .526 | .011 | .411 | .096 |
|  | Lower-bound | 4.007 | 1.000 | 4.007 | .411 | .526 | .011 | .411 | .096 |
| Error(conflict) | Sphericity Assumed | 351.227 | 36 | 9.756 |  |  |  |  |  |
|  | Greenhouse-Geisser | 351.227 | 36.000 | 9.756 |  |  |  |  |  |
|  | Huynh-Feldt | 351.227 | 36.000 | 9.756 |  |  |  |  |  |
|  | Lower-bound | 351.227 | 36.000 | 9.756 |  |  |  |  |  |
| electrode * conflict | Sphericity Assumed | 3.207 | 7 | .458 | 1.369 | .219 | .037 | 9.584 | .578 |
|  | Greenhouse-Geisser | 3.207 | 3.739 | .858 | 1.369 | .250 | .037 | 5.119 | .403 |
|  | Huynh-Feldt | 3.207 | 4.342 | .738 | 1.369 | .244 | .037 | 5.945 | .439 |
|  | Lower-bound | 3.207 | 1.000 | 3.207 | 1.369 | .250 | .037 | 1.369 | .207 |
| electrode * conflict * power | Sphericity Assumed | 2.623 | 7 | .375 | 1.120 | .351 | .030 | 7.840 | .479 |
|  | Greenhouse-Geisser | 2.623 | 3.739 | .702 | 1.120 | .349 | .030 | 4.188 | .333 |
|  | Huynh-Feldt | 2.623 | 4.342 | .604 | 1.120 | .350 | .030 | 4.863 | .362 |
|  | Lower-bound | 2.623 | 1.000 | 2.623 | 1.120 | .297 | .030 | 1.120 | .178 |
| Error(electrode*conflict) | Sphericity Assumed | 84.311 | 252 | .335 |  |  |  |  |  |
|  | Greenhouse-Geisser | 84.311 | 134.601 | .626 |  |  |  |  |  |
|  | Huynh-Feldt | 84.311 | 156.314 | .539 |  |  |  |  |  |
|  | Lower-bound | 84.311 | 36.000 | 2.342 |  |  |  |  |  |
| a. Computed using alpha = .05 | | | | | | | | | |

| **Tests of Within-Subjects Contrasts** | | | | | | | | | | |
| --- | --- | --- | --- | --- | --- | --- | --- | --- | --- | --- |
| Measure: MEASURE_1 | | | | | | | | | | |
| Source | electrode | conflict | Type III Sum of Squares | df | Mean Square | F | Sig. | Partial Eta Squared | Noncent. Parameter | Observed Power^a^ |
| electrode | Linear |  | 1858.810 | 1 | 1858.810 | 44.451 | .000 | .553 | 44.451 | 1.000 |
|  | Quadratic |  | 32.264 | 1 | 32.264 | 32.145 | .000 | .472 | 32.145 | 1.000 |
|  | Cubic |  | 1.460 | 1 | 1.460 | .317 | .577 | .009 | .317 | .085 |
|  | Order 4 |  | .969 | 1 | .969 | .234 | .631 | .006 | .234 | .076 |
|  | Order 5 |  | 15.797 | 1 | 15.797 | 1.630 | .210 | .043 | 1.630 | .237 |
|  | Order 6 |  | 107.124 | 1 | 107.124 | 17.913 | .000 | .332 | 17.913 | .984 |
|  | Order 7 |  | 10.086 | 1 | 10.086 | 2.365 | .133 | .062 | 2.365 | .322 |
| electrode * power | Linear |  | 1.018 | 1 | 1.018 | .024 | .877 | .001 | .024 | .053 |
|  | Quadratic |  | 1.243 | 1 | 1.243 | 1.239 | .273 | .033 | 1.239 | .192 |
|  | Cubic |  | 4.132 | 1 | 4.132 | .898 | .350 | .024 | .898 | .152 |
|  | Order 4 |  | .254 | 1 | .254 | .061 | .806 | .002 | .061 | .057 |
|  | Order 5 |  | 9.327 | 1 | 9.327 | .962 | .333 | .026 | .962 | .159 |
|  | Order 6 |  | .010 | 1 | .010 | .002 | .968 | .000 | .002 | .050 |
|  | Order 7 |  | 2.485 | 1 | 2.485 | .583 | .450 | .016 | .583 | .115 |
| Error(electrode) | Linear |  | 1505.422 | 36 | 41.817 |  |  |  |  |  |
|  | Quadratic |  | 36.134 | 36 | 1.004 |  |  |  |  |  |
|  | Cubic |  | 165.630 | 36 | 4.601 |  |  |  |  |  |
|  | Order 4 |  | 148.997 | 36 | 4.139 |  |  |  |  |  |
|  | Order 5 |  | 348.989 | 36 | 9.694 |  |  |  |  |  |
|  | Order 6 |  | 215.289 | 36 | 5.980 |  |  |  |  |  |
|  | Order 7 |  | 153.541 | 36 | 4.265 |  |  |  |  |  |
| conflict |  | Linear | 22.511 | 1 | 22.511 | 2.307 | .138 | .060 | 2.307 | .315 |
| conflict * power |  | Linear | 4.007 | 1 | 4.007 | .411 | .526 | .011 | .411 | .096 |
| Error(conflict) |  | Linear | 351.227 | 36 | 9.756 |  |  |  |  |  |
| electrode * conflict | Linear | Linear | .002 | 1 | .002 | .002 | .961 | .000 | .002 | .050 |
|  | Quadratic | Linear | .678 | 1 | .678 | 2.612 | .115 | .068 | 2.612 | .350 |
|  | Cubic | Linear | .228 | 1 | .228 | 1.270 | .267 | .034 | 1.270 | .195 |
|  | Order 4 | Linear | .016 | 1 | .016 | .103 | .750 | .003 | .103 | .061 |
|  | Order 5 | Linear | 1.091 | 1 | 1.091 | 2.757 | .106 | .071 | 2.757 | .366 |
|  | Order 6 | Linear | .920 | 1 | .920 | 3.525 | .069 | .089 | 3.525 | .447 |
|  | Order 7 | Linear | .271 | 1 | .271 | 1.634 | .209 | .043 | 1.634 | .238 |
| electrode * conflict * power | Linear | Linear | .193 | 1 | .193 | .208 | .651 | .006 | .208 | .073 |
|  | Quadratic | Linear | .743 | 1 | .743 | 2.862 | .099 | .074 | 2.862 | .377 |
|  | Cubic | Linear | .200 | 1 | .200 | 1.110 | .299 | .030 | 1.110 | .176 |
|  | Order 4 | Linear | .299 | 1 | .299 | 1.965 | .170 | .052 | 1.965 | .276 |
|  | Order 5 | Linear | .864 | 1 | .864 | 2.185 | .148 | .057 | 2.185 | .301 |
|  | Order 6 | Linear | .155 | 1 | .155 | .592 | .446 | .016 | .592 | .116 |
|  | Order 7 | Linear | .169 | 1 | .169 | 1.016 | .320 | .027 | 1.016 | .166 |
| Error(electrode*conflict) | Linear | Linear | 33.388 | 36 | .927 |  |  |  |  |  |
|  | Quadratic | Linear | 9.349 | 36 | .260 |  |  |  |  |  |
|  | Cubic | Linear | 6.475 | 36 | .180 |  |  |  |  |  |
|  | Order 4 | Linear | 5.487 | 36 | .152 |  |  |  |  |  |
|  | Order 5 | Linear | 14.239 | 36 | .396 |  |  |  |  |  |
|  | Order 6 | Linear | 9.394 | 36 | .261 |  |  |  |  |  |
|  | Order 7 | Linear | 5.979 | 36 | .166 |  |  |  |  |  |
| a. Computed using alpha = .05 | | | | | | | | | | |

| **Levene's Test of Equality of Error Variances^a^** | | | | |
| --- | --- | --- | --- | --- |
|  | F | df1 | df2 | Sig. |
| F3congruent | 2.494 | 1 | 36 | .123 |
| F3incongruent | 3.605 | 1 | 36 | .066 |
| F4congruent | 5.538 | 1 | 36 | .024 |
| F4incongruent | 8.010 | 1 | 36 | .008 |
| FZ congruent | 7.969 | 1 | 36 | .008 |
| FZ incongruent | 10.497 | 1 | 36 | .003 |
| C3congruent | 1.215 | 1 | 36 | .278 |
| C3incongruent | .050 | 1 | 36 | .824 |
| C4congruent | 5.619 | 1 | 36 | .023 |
| C4incongruent | 4.947 | 1 | 36 | .032 |
| CZ congruent | .265 | 1 | 36 | .610 |
| CZ incongruent | .352 | 1 | 36 | .557 |
| CP1congruent | .050 | 1 | 36 | .825 |
| CP1incongruent | .389 | 1 | 36 | .537 |
| CP2congruent | .434 | 1 | 36 | .514 |
| CP2incongruent | .576 | 1 | 36 | .453 |
| Tests the null hypothesis that the error variance of the dependent variable is equal across groups. | | | | |
| a. Design: + power  Within Subjects Design: electrode + conflict + electrode * conflict | | | | |

| **Tests of Between-Subjects Effects** | | | | | | | | |
| --- | --- | --- | --- | --- | --- | --- | --- | --- |
| Measure: MEASURE_1 | | | | | | | | |
| Transformed Variable: Average | | | | | | | | |
| Source | Type III Sum of Squares | df | Mean Square | F | Sig. | Partial Eta Squared | Noncent. Parameter | Observed Power^a^ |
|  | 8133.656 | 1 | 8133.656 | 61.840 | .000 | .632 | 61.840 | 1.000 |
| power | .043 | 1 | .043 | .000 | .986 | .000 | .000 | .050 |
| Error | 4735.009 | 36 | 131.528 |  |  |  |  |  |
| a. Computed using alpha = .05 | | | | | | | | |

**Estimated Marginal Means**

**1. power**

| **Estimates** | | | | |
| --- | --- | --- | --- | --- |
| Measure: MEASURE_1 | | | | |
| power | Mean | Std. Error | 95% Confidence Interval | |
|  |  |  | Lower Bound | Upper Bound |
| 1.00 | 3.666 | .658 | 2.332 | 5.000 |
| 2.00 | 3.649 | .658 | 2.315 | 4.983 |

| **Pairwise Comparisons** | | | | | | |
| --- | --- | --- | --- | --- | --- | --- |
| Measure: MEASURE_1 | | | | | | |
| (I) power | (J) power | Mean Difference (I-J) | Std. Error | Sig.^a^ | 95% Confidence Interval for Difference^a^ | |
|  |  |  |  |  | Lower Bound | Upper Bound |
| 1.00 | 2.00 | .017 | .930 | .986 | -1.870 | 1.903 |
| 2.00 | 1.00 | -.017 | .930 | .986 | -1.903 | 1.870 |
| Based on estimated marginal means | | | | | | |
| a. Adjustment for multiple comparisons: Least Significant Difference (equivalent to no adjustments). | | | | | | |

| **Univariate Tests** | | | | | | | | |
| --- | --- | --- | --- | --- | --- | --- | --- | --- |
| Measure: MEASURE_1 | | | | | | | | |
|  | Sum of Squares | df | Mean Square | F | Sig. | Partial Eta Squared | Noncent. Parameter | Observed Power^a^ |
| Contrast | .003 | 1 | .003 | .000 | .986 | .000 | .000 | .050 |
| Error | 295.938 | 36 | 8.221 |  |  |  |  |  |
| The F tests the effect of power. This test is based on the linearly independent pairwise comparisons among the estimated marginal means. | | | | | | | | |
| a. Computed using alpha = .05 | | | | | | | | |

**2. electrode**

| **Estimates** | | | | |
| --- | --- | --- | --- | --- |
| Measure: MEASURE_1 | | | | |
| electrode | Mean | Std. Error | 95% Confidence Interval | |
|  |  |  | Lower Bound | Upper Bound |
| 1 | 1.295 | .622 | .034 | 2.556 |
| 2 | 2.244 | .703 | .818 | 3.671 |
| 3 | 1.596 | .708 | .161 | 3.031 |
| 4 | 3.041 | .414 | 2.201 | 3.882 |
| 5 | 4.584 | .502 | 3.565 | 5.602 |
| 6 | 4.075 | .561 | 2.938 | 5.212 |
| 7 | 5.784 | .494 | 4.781 | 6.786 |
| 8 | 6.641 | .555 | 5.516 | 7.766 |

| **Pairwise Comparisons** | | | | | | |
| --- | --- | --- | --- | --- | --- | --- |
| Measure: MEASURE_1 | | | | | | |
| (I) electrode | (J) electrode | Mean Difference (I-J) | Std. Error | Sig.^b^ | 95% Confidence Interval for Difference^b^ | |
|  |  |  |  |  | Lower Bound | Upper Bound |
| 1 | 2 | -.950^*^ | .466 | .049 | -1.895 | -.004 |
|  | 3 | -.301 | .308 | .335 | -.925 | .323 |
|  | 4 | -1.746^*^ | .436 | .000 | -2.630 | -.862 |
|  | 5 | -3.289^*^ | .551 | .000 | -4.407 | -2.170 |
|  | 6 | -2.780^*^ | .580 | .000 | -3.957 | -1.603 |
|  | 7 | -4.489^*^ | .678 | .000 | -5.864 | -3.113 |
|  | 8 | -5.346^*^ | .737 | .000 | -6.841 | -3.852 |
| 2 | 1 | .950^*^ | .466 | .049 | .004 | 1.895 |
|  | 3 | .649^*^ | .308 | .042 | .024 | 1.274 |
|  | 4 | -.797 | .627 | .212 | -2.068 | .474 |
|  | 5 | -2.339^*^ | .451 | .000 | -3.253 | -1.425 |
|  | 6 | -1.831^*^ | .583 | .003 | -3.013 | -.648 |
|  | 7 | -3.539^*^ | .754 | .000 | -5.069 | -2.009 |
|  | 8 | -4.397^*^ | .711 | .000 | -5.838 | -2.956 |
| 3 | 1 | .301 | .308 | .335 | -.323 | .925 |
|  | 2 | -.649^*^ | .308 | .042 | -1.274 | -.024 |
|  | 4 | -1.446^*^ | .560 | .014 | -2.581 | -.310 |
|  | 5 | -2.988^*^ | .537 | .000 | -4.077 | -1.898 |
|  | 6 | -2.479^*^ | .562 | .000 | -3.620 | -1.339 |
|  | 7 | -4.188^*^ | .731 | .000 | -5.669 | -2.706 |
|  | 8 | -5.046^*^ | .740 | .000 | -6.546 | -3.546 |
| 4 | 1 | 1.746^*^ | .436 | .000 | .862 | 2.630 |
|  | 2 | .797 | .627 | .212 | -.474 | 2.068 |
|  | 3 | 1.446^*^ | .560 | .014 | .310 | 2.581 |
|  | 5 | -1.542^*^ | .401 | .000 | -2.356 | -.729 |
|  | 6 | -1.034^*^ | .387 | .011 | -1.819 | -.249 |
|  | 7 | -2.742^*^ | .328 | .000 | -3.407 | -2.078 |
|  | 8 | -3.600^*^ | .426 | .000 | -4.464 | -2.736 |
| 5 | 1 | 3.289^*^ | .551 | .000 | 2.170 | 4.407 |
|  | 2 | 2.339^*^ | .451 | .000 | 1.425 | 3.253 |
|  | 3 | 2.988^*^ | .537 | .000 | 1.898 | 4.077 |
|  | 4 | 1.542^*^ | .401 | .000 | .729 | 2.356 |
|  | 6 | .509 | .334 | .137 | -.170 | 1.187 |
|  | 7 | -1.200^*^ | .436 | .009 | -2.084 | -.316 |
|  | 8 | -2.058^*^ | .332 | .000 | -2.732 | -1.384 |
| 6 | 1 | 2.780^*^ | .580 | .000 | 1.603 | 3.957 |
|  | 2 | 1.831^*^ | .583 | .003 | .648 | 3.013 |
|  | 3 | 2.479^*^ | .562 | .000 | 1.339 | 3.620 |
|  | 4 | 1.034^*^ | .387 | .011 | .249 | 1.819 |
|  | 5 | -.509 | .334 | .137 | -1.187 | .170 |
|  | 7 | -1.708^*^ | .321 | .000 | -2.360 | -1.057 |
|  | 8 | -2.566^*^ | .321 | .000 | -3.216 | -1.916 |
| 7 | 1 | 4.489^*^ | .678 | .000 | 3.113 | 5.864 |
|  | 2 | 3.539^*^ | .754 | .000 | 2.009 | 5.069 |
|  | 3 | 4.188^*^ | .731 | .000 | 2.706 | 5.669 |
|  | 4 | 2.742^*^ | .328 | .000 | 2.078 | 3.407 |
|  | 5 | 1.200^*^ | .436 | .009 | .316 | 2.084 |
|  | 6 | 1.708^*^ | .321 | .000 | 1.057 | 2.360 |
|  | 8 | -.858^*^ | .243 | .001 | -1.351 | -.365 |
| 8 | 1 | 5.346^*^ | .737 | .000 | 3.852 | 6.841 |
|  | 2 | 4.397^*^ | .711 | .000 | 2.956 | 5.838 |
|  | 3 | 5.046^*^ | .740 | .000 | 3.546 | 6.546 |
|  | 4 | 3.600^*^ | .426 | .000 | 2.736 | 4.464 |
|  | 5 | 2.058^*^ | .332 | .000 | 1.384 | 2.732 |
|  | 6 | 2.566^*^ | .321 | .000 | 1.916 | 3.216 |
|  | 7 | .858^*^ | .243 | .001 | .365 | 1.351 |
| Based on estimated marginal means | | | | | | |
| *. The mean difference is significant at the .05 level. | | | | | | |
| b. Adjustment for multiple comparisons: Least Significant Difference (equivalent to no adjustments). | | | | | | |

| **Multivariate Tests** | | | | | | | | |
| --- | --- | --- | --- | --- | --- | --- | --- | --- |
|  | Value | F | Hypothesis df | Error df | Sig. | Partial Eta Squared | Noncent. Parameter | Observed Power^b^ |
| Pillai's trace | .776 | 14.861^a^ | 7.000 | 30.000 | .000 | .776 | 104.025 | 1.000 |
| Wilks' lambda | .224 | 14.861^a^ | 7.000 | 30.000 | .000 | .776 | 104.025 | 1.000 |
| Hotelling's trace | 3.468 | 14.861^a^ | 7.000 | 30.000 | .000 | .776 | 104.025 | 1.000 |
| Roy's largest root | 3.468 | 14.861^a^ | 7.000 | 30.000 | .000 | .776 | 104.025 | 1.000 |
| Each F tests the multivariate effect of electrode. These tests are based on the linearly independent pairwise comparisons among the estimated marginal means. | | | | | | | | |
| a. Exact statistic | | | | | | | | |
| b. Computed using alpha = .05 | | | | | | | | |

**3. conflict**

| **Estimates** | | | | |
| --- | --- | --- | --- | --- |
| Measure: MEASURE_1 | | | | |
| conflict | Mean | Std. Error | 95% Confidence Interval | |
|  |  |  | Lower Bound | Upper Bound |
| 1 | 3.850 | .463 | 2.910 | 4.790 |
| 2 | 3.465 | .500 | 2.451 | 4.479 |

| **Pairwise Comparisons** | | | | | | |
| --- | --- | --- | --- | --- | --- | --- |
| Measure: MEASURE_1 | | | | | | |
| (I) conflict | (J) conflict | Mean Difference (I-J) | Std. Error | Sig.^a^ | 95% Confidence Interval for Difference^a^ | |
|  |  |  |  |  | Lower Bound | Upper Bound |
| 1 | 2 | .385 | .253 | .138 | -.129 | .899 |
| 2 | 1 | -.385 | .253 | .138 | -.899 | .129 |
| Based on estimated marginal means | | | | | | |
| a. Adjustment for multiple comparisons: Least Significant Difference (equivalent to no adjustments). | | | | | | |

| **Multivariate Tests** | | | | | | | | |
| --- | --- | --- | --- | --- | --- | --- | --- | --- |
|  | Value | F | Hypothesis df | Error df | Sig. | Partial Eta Squared | Noncent. Parameter | Observed Power^b^ |
| Pillai's trace | .060 | 2.307^a^ | 1.000 | 36.000 | .138 | .060 | 2.307 | .315 |
| Wilks' lambda | .940 | 2.307^a^ | 1.000 | 36.000 | .138 | .060 | 2.307 | .315 |
| Hotelling's trace | .064 | 2.307^a^ | 1.000 | 36.000 | .138 | .060 | 2.307 | .315 |
| Roy's largest root | .064 | 2.307^a^ | 1.000 | 36.000 | .138 | .060 | 2.307 | .315 |
| Each F tests the multivariate effect of conflict. These tests are based on the linearly independent pairwise comparisons among the estimated marginal means. | | | | | | | | |
| a. Exact statistic | | | | | | | | |
| b. Computed using alpha = .05 | | | | | | | | |

| **4. power * electrode** | | | | | |
| --- | --- | --- | --- | --- | --- |
| Measure: MEASURE_1 | | | | | |
| power | electrode | Mean | Std. Error | 95% Confidence Interval | |
|  |  |  |  | Lower Bound | Upper Bound |
| 1.00 | 1 | 1.005 | .879 | -.778 | 2.788 |
|  | 2 | 2.455 | .995 | .438 | 4.472 |
|  | 3 | 1.646 | 1.001 | -.384 | 3.675 |
|  | 4 | 2.895 | .586 | 1.707 | 4.083 |
|  | 5 | 4.810 | .710 | 3.370 | 6.250 |
|  | 6 | 4.103 | .793 | 2.495 | 5.711 |
|  | 7 | 5.638 | .699 | 4.220 | 7.056 |
|  | 8 | 6.776 | .784 | 5.185 | 8.367 |
| 2.00 | 1 | 1.585 | .879 | -.198 | 3.368 |
|  | 2 | 2.034 | .995 | .017 | 4.051 |
|  | 3 | 1.546 | 1.001 | -.483 | 3.575 |
|  | 4 | 3.188 | .586 | 1.999 | 4.376 |
|  | 5 | 4.357 | .710 | 2.917 | 5.797 |
|  | 6 | 4.047 | .793 | 2.440 | 5.655 |
|  | 7 | 5.929 | .699 | 4.512 | 7.347 |
|  | 8 | 6.507 | .784 | 4.916 | 8.098 |

| **5. power * conflict** | | | | | |
| --- | --- | --- | --- | --- | --- |
| Measure: MEASURE_1 | | | | | |
| power | conflict | Mean | Std. Error | 95% Confidence Interval | |
|  |  |  |  | Lower Bound | Upper Bound |
| 1.00 | 1 | 3.777 | .655 | 2.448 | 5.106 |
|  | 2 | 3.555 | .707 | 2.121 | 4.989 |
| 2.00 | 1 | 3.923 | .655 | 2.594 | 5.252 |
|  | 2 | 3.376 | .707 | 1.941 | 4.810 |

| **6. electrode * conflict** | | | | | |
| --- | --- | --- | --- | --- | --- |
| Measure: MEASURE_1 | | | | | |
| electrode | conflict | Mean | Std. Error | 95% Confidence Interval | |
|  |  |  |  | Lower Bound | Upper Bound |
| 1 | 1 | 1.390 | .639 | .095 | 2.686 |
|  | 2 | 1.200 | .637 | -.093 | 2.493 |
| 2 | 1 | 2.523 | .720 | 1.062 | 3.983 |
|  | 2 | 1.966 | .713 | .520 | 3.413 |
| 3 | 1 | 1.748 | .725 | .278 | 3.218 |
|  | 2 | 1.444 | .720 | -.016 | 2.904 |
| 4 | 1 | 3.245 | .440 | 2.353 | 4.136 |
|  | 2 | 2.838 | .438 | 1.950 | 3.726 |
| 5 | 1 | 4.916 | .496 | 3.911 | 5.921 |
|  | 2 | 4.251 | .539 | 3.158 | 5.345 |
| 6 | 1 | 4.227 | .543 | 3.125 | 5.328 |
|  | 2 | 3.924 | .608 | 2.690 | 5.157 |
| 7 | 1 | 5.928 | .478 | 4.959 | 6.898 |
|  | 2 | 5.639 | .553 | 4.517 | 6.760 |
| 8 | 1 | 6.824 | .532 | 5.744 | 7.903 |
|  | 2 | 6.459 | .610 | 5.221 | 7.697 |

| **7. power * electrode * conflict** | | | | | | |
| --- | --- | --- | --- | --- | --- | --- |
| Measure: MEASURE_1 | | | | | | |
| power | electrode | conflict | Mean | Std. Error | 95% Confidence Interval | |
|  |  |  |  |  | Lower Bound | Upper Bound |
| 1.00 | 1 | 1 | .886 | .903 | -.946 | 2.718 |
|  |  | 2 | 1.124 | .902 | -.705 | 2.952 |
|  | 2 | 1 | 2.706 | 1.018 | .641 | 4.772 |
|  |  | 2 | 2.203 | 1.008 | .158 | 4.248 |
|  | 3 | 1 | 1.760 | 1.025 | -.319 | 3.839 |
|  |  | 2 | 1.531 | 1.018 | -.533 | 3.595 |
|  | 4 | 1 | 2.962 | .622 | 1.702 | 4.223 |
|  |  | 2 | 2.828 | .619 | 1.572 | 4.084 |
|  | 5 | 1 | 5.120 | .701 | 3.698 | 6.541 |
|  |  | 2 | 4.501 | .763 | 2.954 | 6.047 |
|  | 6 | 1 | 4.240 | .768 | 2.683 | 5.797 |
|  |  | 2 | 3.966 | .860 | 2.221 | 5.710 |
|  | 7 | 1 | 5.670 | .676 | 4.299 | 7.041 |
|  |  | 2 | 5.606 | .782 | 4.020 | 7.192 |
|  | 8 | 1 | 6.873 | .753 | 5.346 | 8.400 |
|  |  | 2 | 6.679 | .863 | 4.928 | 8.430 |
| 2.00 | 1 | 1 | 1.894 | .903 | .062 | 3.726 |
|  |  | 2 | 1.276 | .902 | -.553 | 3.104 |
|  | 2 | 1 | 2.339 | 1.018 | .273 | 4.404 |
|  |  | 2 | 1.730 | 1.008 | -.316 | 3.775 |
|  | 3 | 1 | 1.735 | 1.025 | -.344 | 3.814 |
|  |  | 2 | 1.357 | 1.018 | -.707 | 3.421 |
|  | 4 | 1 | 3.527 | .622 | 2.267 | 4.788 |
|  |  | 2 | 2.848 | .619 | 1.592 | 4.104 |
|  | 5 | 1 | 4.712 | .701 | 3.291 | 6.134 |
|  |  | 2 | 4.002 | .763 | 2.455 | 5.548 |
|  | 6 | 1 | 4.213 | .768 | 2.656 | 5.770 |
|  |  | 2 | 3.882 | .860 | 2.137 | 5.626 |
|  | 7 | 1 | 6.187 | .676 | 4.816 | 7.558 |
|  |  | 2 | 5.671 | .782 | 4.086 | 7.257 |
|  | 8 | 1 | 6.774 | .753 | 5.247 | 8.301 |
|  |  | 2 | 6.240 | .863 | 4.489 | 7.990 |
